# Supplementary material for: Lessons from SARS-CoV-2 in India: A data-driven framework for pandemic resilience
Source: Sci Adv. 2022 Jun 17;8(24):eabp8621. doi: 10.1126/sciadv.abp8621 (PMC9205583; doi:10.1126/sciadv.abp8621)
Supplement: 20220617-1 [file sciadv.abp8621.v1.pdf]

CORONAVIRUS

Lessons from SARS-CoV-2 in India: A data-driven framework for pandemic resilience

Maxwell Salvatore[1,2,3]†, Soumik Purkayastha[1]†, Lakshmi Ganapathi[4], Rupam Bhattacharyya[1], Ritoban Kundu[1], Lauren Zimmermann[1,2], Debashree Ray[5,6], Aditi Hazra[7], Michael Kleinsasser[1], Sunil Solomon[8], Ramnath Subbaraman[9], Bhramar Mukherjee[1,2,3]*

Copyright © 2022 The Authors, some rights reserved; exclusive licensee American Association for the Advancement of Science. No claim to original U.S. Government Works. Distributed under a Creative Commons Attribution License 4.0 (CC BY).

India experienced a massive surge in SARS-CoV-2 infections and deaths during April to June 2021 despite having controlled the epidemic relatively well during 2020. Using counterfactual predictions from epidemiological disease transmission models, we produce evidence in support of how strengthening public health interventions early would have helped control transmission in the country and significantly reduced mortality during the second wave, even without harsh lockdowns. We argue that enhanced surveillance at district, state, and national levels and constant assessment of risk associated with increased transmission are critical for future pandemic responsiveness. Building on our retrospective analysis, we provide a tiered data-driven framework for timely escalation of future interventions as a tool for policy-makers.

INTRODUCTION

The first case of severe acute respiratory syndrome coronavirus 2 (SARS-CoV-2) in India [where 18% of the world's population lives (*1*)] was reported on 30 January 2020 (*2*). India was proactive in implementing a suite of timely public health interventions (PHIs) in the first wave of its epidemic. On 3 March 2020, with few confirmed coronavirus disease 2019 (COVID-19) cases and no reported deaths, India began strict border controls (*3*). Within 2 weeks of the World Health Organization (WHO) declaring COVID-19 a pandemic (*4*), India made a widely debated decision to implement a 21-day national lockdown starting 25 March 2020, with only 536 reported cases and 11 COVID-19–attributable deaths (*5*). The lockdown was extended to four distinct phases that lasted until 31 May 2020 (*6*–*8*). During this lockdown, India scaled up testing and treatment. Public acceptance of masks, avoidance of social gatherings, and adoption of other PHIs was high (*9*). A gradual relaxation of nationwide restrictions started in monthly phases from 1 June 2020 (*10*). Although daily case counts continued to increase during and after the lockdown until September 2020, the control measures were effective in decelerating the rate of transmission (*11*). However, the economic and social costs of the 2020 national lockdown were substantial (*12*–*15*).

After the first wave's peak in September 2020, incidence declined steadily to less than 10,000 daily new cases and 150 daily deaths in February 2021 (*16*); however, the third national serosurvey estimated a substantial infection underascertainment rate, suggesting that only 1 in 25 to 30 infections was detected (*17*). As the country further relaxed restrictions, COVID-appropriate behaviors diminished (*9*): Crowded public transportation systems restarted, and large gatherings including religious and social events, political rallies, and mass protests, all part of the cultural tapestry in India, took place without meaningful adherence to masks. Following multiple successful vaccine trials (*9*), India rolled out its COVID-19 vaccination campaign on 16 January 2021 (*18*); however, only 4% of the population had received at least one dose by 1 April 2021 (*16*).

After a decline for about 4 months, three states (Maharashtra, Punjab, and Chhattisgarh) noted an increase in cases in January 2021, with the 7-day trailing average national effective reproduction number, R_t, crossing the threshold of one on 19 February 2021. No nationwide PHI measures were reintroduced following the initial indications of a resurgence in transmission. The first strong nonlockdown PHI measures started on 28 March 2021 in Maharashtra, followed by a comprehensive lockdown in the state on 14 April 2021 (*19*), when India was already witnessing a staggering growth in infections. A massive humanitarian catastrophe unfolded that was termed as an "unprecedented public health crisis" (*20*). Health care infrastructure collapsed under surges in hospitalizations (*21*), while crematoriums were overflowing (*22*) with evidence suggesting that the actual death toll far exceeded official numbers (*23*–*25*). The lack of timely and stringent preventive PHI measures and the role of emerging variants in a largely unvaccinated population defined the conversations around India's second wave (*26*, *27*).

In other parts of the world, late 2020 saw resurgent transmission, with new SARS-CoV-2 variants being identified in the United Kingdom (Alpha/B.1.1.7), Brazil (Gamma/P.1), and South Africa (Beta/B.1.351) (*28*). In December 2020, the Ministry of Health and Family Welfare in India launched a surveillance initiative formally referred to as the Indian SARS-CoV-2 Genome Sequencing Consortia (INSACOG) (*29*) to track the virus' evolution and identify new variants of concern (VOCs). The global spread of other VOCs was mirrored by the identification of B.1.1.7, B.1.351, and P.1 in India (*29*). The B.1.617(.1/2/3) lineage was first detected in December 2020 in India and soon became a dominant lineage, particularly in Maharashtra (*30*). Between January and February 2021, the B.1.617 lineage, including Delta (B.1.617.2) and Kappa (B.1.617.1), was detected in about 60% of the 361 sequenced cases sampled in Maharashtra (*31*), and the B.1.617.2 sublineage

[1]Department of Biostatistics, University of Michigan, Ann Arbor, MI, USA. [2]Center for Precision Health Data Science, University of Michigan, Ann Arbor, MI, USA. [3]Department of Epidemiology, University of Michigan, Ann Arbor, MI, USA. [4]Division of Infectious Diseases, Boston Children's Hospital and Harvard Medical School, Boston, MA, USA. [5]Department of Epidemiology, Bloomberg School of Public Health, Johns Hopkins University, Baltimore, MD, USA. [6]Department of Biostatistics, Bloomberg School of Public Health, Johns Hopkins University, Baltimore, MD, USA. [7]Department of Medicine, Brigham and Women's Hospital and Harvard Medical School, Boston, MA, USA. [8]Department of Medicine, Johns Hopkins University School of Medicine, Baltimore, MD, USA. [9]Department of Public Health and Community Medicine and Center for Global Public Health, Tufts University School of Medicine, Boston, MA, USA.
*Corresponding author. Email: bhramar@umich.edu
†These authors contributed equally to this work.

was marked as a VOC in early May by WHO (*32*). Considerable regional heterogeneity was noted with respect to the dominant lineage in India (*33*–*35*). Given the increased transmissibility of some of these variants (*36*) and the devastating impact of India's second wave, understanding whether and how appropriately timed PHI may have averted the infections and mortality during the second wave is critical to quantify.

Here, we present a retrospective epidemiological analysis of the second COVID-19 wave in India. We aim to understand the timing, composition, and intensity of PHI that, if applied nationally, might have blunted the sharp rise of India's second wave. In addition, given the adverse social, economic, and health repercussions of India's 2020 national lockdown, we evaluate whether less restrictive interventions may have been equally effective if timed appropriately. On the basis of the quantitative results, we propose a tiered intervention framework aimed at curbing future COVID-19 waves arising from highly transmissible emerging variants that could have immune escape properties in populations where vaccination rates remain suboptimal.

The role of early PHIs

Broadly, the goals of PHI are to (i) "flatten the curve" during periods of high transmission to ensure that the health system and resources are not overwhelmed; (ii) prolong time periods with relatively low transmission when pandemic control measures such as testing, contact tracing, and treatment capacity can be increased; (iii) learn more about properties of emerging variants; and (iv) most importantly, buy time for increasing vaccination, which considerably mitigates the severe adverse health impacts of COVID-19. While the nationwide lockdown implemented in the first wave was successful in terms of flattening the curve in India, the second wave only saw statewide measures [see section S1 for a timeline of PHI in Maharashtra (table S1) and nationally in India (table S2)]. Could timely nationwide PHI have mitigated the second wave and reduced COVID-related mortality in India?

To estimate the impact of potential PHI on the number of reported cases and deaths during the second COVID-19 wave in India, we emulate a counterfactual prediction using an extended susceptible-antibody-infected-removed (eSAIR) model. All data that we use in the following sections are publicly available at covid19india.org (*16*) and covind19.org (*5*) and assume an Indian population of 1.34 billion (*1*). The eSAIR model expands on the extended susceptible-infected-removed (eSIR) model, first developed by Wang and colleagues (*37*) to study the COVID-19 outbreak in China and subsequently used in studies of COVID-19 elsewhere (*5*, *38*). The eSAIR model extends the eSIR model in three ways: (i) It incorporates information on population-level seroprevalence. (ii) It considers multiple virus strains with differing transmission rates over time, and (iii) it allows for the possibility of reinfection. A schematic summary of the compartments and associated transmission dynamics of the eSAIR model is presented in Fig. 1, with the system of differential equations and additional implementation details of the Markov chain Monte Carlo (MCMC) procedure provided in Materials and Methods and sections S2.1.1 and S2.1.2 (including a more detailed schematic in fig. S1).

To adapt this model specifically for India, we estimate the proportion of population having SARS-CoV-2 antibodies (in compartment A in Fig. 1) at any time t via a seroprevalence function $\alpha(t)$ using information from the four serial serosurveys conducted in India (fig. S2). For constructing a sequence of time-varying transmission

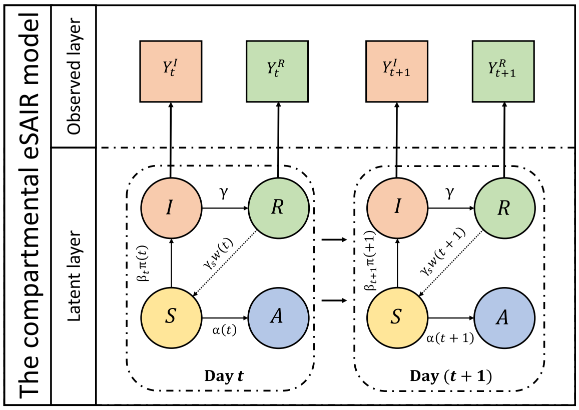

Fig. 1. A schematic representation of the compartments of the eSAIR model. This graphical depiction of the compartments of the model shows the flow between compartments and the key rates describing these transitions.

rates, β_t, we use available data on time-varying prevalence of multiple strains and their respective transmissibility relative to the ancestral strain based on existing data (fig. S3). The distribution of reinfection rate parameters $w(t)$ and γ_s is based on published literature. Last, we quantify the effect of an intervention on case counts through a plausible intervention/modifier schedule, $\pi(t)$, which changes the disease transmission rate from β_t (at a given time t) to $\beta_t\pi(t)$. References informing parameter choices and assumptions are presented in Materials and Methods and section S2.1.1.

Estimating the effect of PHIs in India

The intervention effect or $\pi(t)$ schedules that we use in our models are empirically derived from relative changes in the time-varying effective reproduction number R_t in response to observed interventions in India. To explain this process, in Fig. 2A, we show an annotated daily case plot for the state of Maharashtra (which has contributed the largest number of cases and deaths in India so far), showing key dates when major PHIs were introduced from 15 February to 31 July 2021. The exact PHIs that were implemented in Maharashtra during this period are presented in table S1, so that the changes in R_t can be mapped to observed actions. These reported daily case counts (Fig. 2A) are first used to calculate the estimated time-varying reproduction number R_t (Fig. 2B) (*39*, *40*). The raw $\pi(t)$ schedule is obtained by taking the ratio of R_t over the 7-day average R_t during the week before the intervention start date. The estimated $\pi(t)$ is a smoothed version of the raw ratio values (Fig. 2C). Intervention effects are estimated during prelockdown, lockdown, and subsequent unlock periods. This framework attributes the changes to the R_t trajectory as a potential consequence of the PHI, although there are other contemporaneous confounding processes, including increasing immunity (through natural infections and vaccination) and a change in the variant distribution [e.g., rise in proportion of cases caused by the Delta variant (*41*)]. We cannot make direct claims of causality here, and the actual causal effect of PHI may be attenuated compared to the modeled intervention effects. Therefore, as a sensitivity analysis, we consider four intervention schedules of various strengths, as shown in Fig. 2D, to assess the robustness of our results to the choice of $\pi(t)$.

To construct these four $\pi(t)$ schedules, we first make use of three actual PHI scenarios: (i) prelockdown interventions implemented

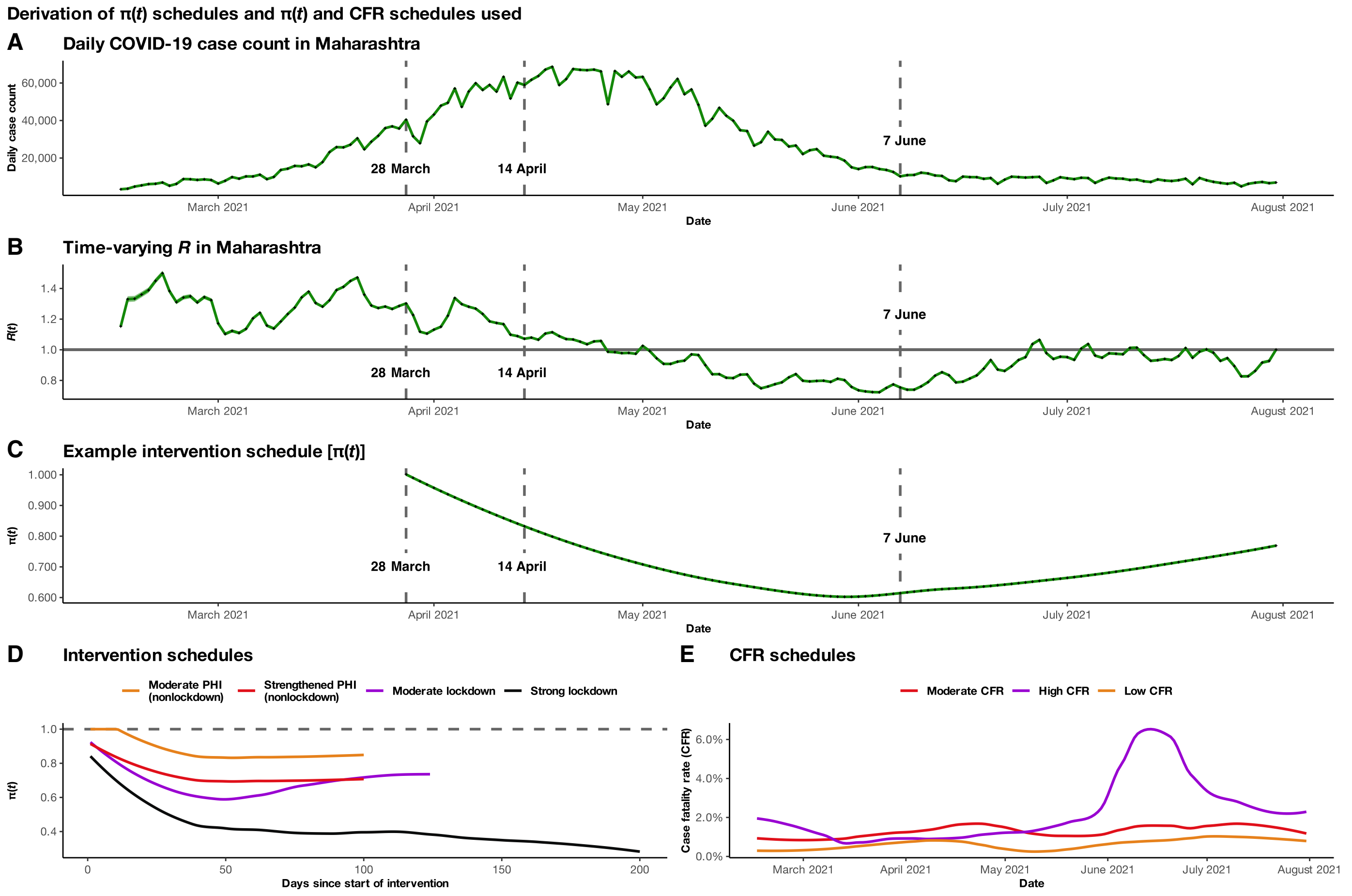

Fig. 2. A π(*t*) schedule derivation schematic corresponding to PHIs that were implemented in Maharashtra from March to July 2021. Three key intervention dates are marked: 28 March, representing the initiation of nonlockdown PHI; 14 April, representing the start of a lockdown; and 7 June, marking the beginning of relaxation of lockdown restrictions. The daily case counts in (**A**) are used to calculate the time-varying effective reproduction number R_t in (**B**) [using the estimate_R function from the EpiEstim package in R (*39*, *40*) with method = "parametric_si", mean_si = 7, and std_si = 4.5]. The LOESS-smoothed π(*t*) schedule is the relative change in R_t (relative to the previous 7-day average) in response to the institution of intervention measures beginning on 28 March 2021 (**C**). The intervention schedules π(*t*) for strong lockdown (black), moderate lockdown (purple), strengthened PHI (nonlockdown; red), and moderate PHI (nonlockdown; orange) are presented in (**D**). The strong lockdown effect represents a smoothed ratio of the estimate effective reproduction number, R_t, after the implementation of the nationwide lockdown in India, initiated on 25 March 2020. The moderate lockdown effect is derived from interventions in Maharashtra that began on 14 April 2021 (last observation carried forward). Strengthened PHI (nonlockdown) effect is derived from the prelockdown phase in Maharashtra from 28 March to 13 April 2021, while moderate PHI (nonlockdown) effect is derived from interventions in that same time period in Maharashtra but attenuated by 20%. The schedules for high (purple), moderate (red), and low (orange) case fatality rates (CFRs) using observed data from 15 February to 30 June 2021 are shown in (**E**). These are LOESS smoothed of the observed daily CFR (daily deaths over daily cases from 14 days prior) in Maharashtra, India, and Kerala, respectively.

in Maharashtra's second wave from 28 March to 13 April 2021, which we refer to as a strengthened PHI (nonlockdown) effect; (ii) the lockdown during Maharashtra's second wave from 14 April to 7 June 2021, which we refer to as a moderate lockdown effect; and (iii) the national lockdown during the first wave from 25 March to May 2020, which we refer to as a strong lockdown effect. The specific PHIs implemented in India or Maharashtra within these time windows that may have contributed to the observed intervention effects are described in table S3. We derived a fourth scenario, which we refer to as a moderate PHI (nonlockdown) effect, by reducing the estimated effect of the strengthened PHI scenario by 20%. This scenario is meant to represent the weaker effect of less intensive interventions that might have been socially and politically feasible to implement early in the second wave. A more detailed description of the modifying intervention effect schedules under each scenario is provided in section S2.1.1.

We selected different start dates for the initiation of these intervention schedules based on trailing 7-day average R_t (hereafter simply R_t unless otherwise specified) thresholds (fig. S4): (i) moderate PHI (nonlockdown), beginning when R_t first went above 1 (19 February); (ii) strengthened PHI (nonlockdown), beginning when R_t first crossed 1.2 (13 March); and (iii) a moderate lockdown, beginning when R_t first crossed 1.4 (19 March). We also consider scenarios where the institution of a moderate lockdown was delayed until 30 March and 15 April, which allows for the assessment of the impact of timing of these interventions on pandemic outcomes.

RESULTS

Intervention effect on cases

If moderate PHI (nonlockdown) had been implemented on 19 February 2021 (and kept in place through the prediction period), it would have contained case counts at a low level, although there is evidence of mild increase in case counts through the end of the prediction period (Fig. 3A). The model predicts that such an intervention would have prevented 17.0 million cases [95% credible interval (CI): [8.5, 18.7]], a 91.3% reduction (95% CI: [45.3%, 100.0%]) through 15 June (italicized in Table 1), meaning early, sustained nonlockdown interventions might have avoided a resurgence and the need for lockdown-level measures. Similarly, the strengthened PHI (nonlockdown) implemented on 13 March would have also successfully suppressed case counts through the prediction period [17.5 (95% CI: [13.0, 18.3]) million cases averted through 15 June 2021, representing a 95.5% (95% CI: [71.2%, 100.0%]) reduction; italicized in Table 1].

Implementing a lockdown during an optimal time window has short- and long-term benefits with respect to the degree of reduction in case counts (Fig. 3A). The predicted case counts would decline quickly if a moderate lockdown was instituted in mid- to late-March 2021, with daily case counts peaking around 25,000 and 52,000, respectively (instead of the observed peak of 414,280 daily cases in May). To put this in perspective, a moderate lockdown starting on 19 March will result in the prevention of 17.2 million reported cases (95% CI: [13.0, 18.1]), a 95.0% reduction (95% CI: [71.7%, 100.0%]; italicized in Table 1) through 15 June 2021. A lockdown with this effect would continue to have benefits through 15 June if it was implemented as late as 30 March [85.3% (95% CI: [50.5%, 100.0%]) of cases averted through 15 June; italicized in Table 1]. This suggests that the timing

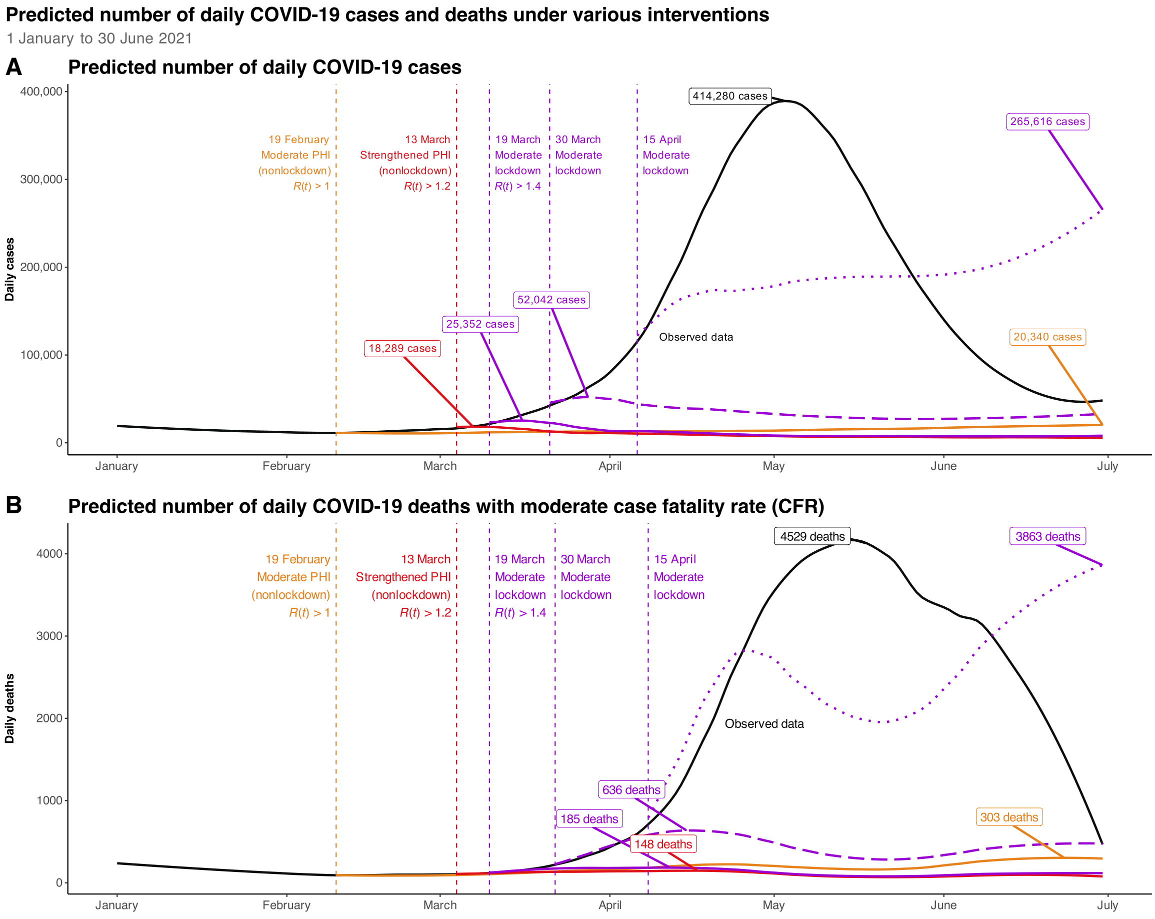

Fig. 3. Predicted COVID-19 case and death counts under various intervention scenarios in India from 1 January to 30 June 2021. Observed (black), predicted daily case counts (**A**), and predicted daily death counts assuming a moderate CFR (**B**) from 1 January to 30 June 2021 in India under intervention scenarios starting on different dates. Predictions under moderate PHI (nonlockdown; orange), strengthened PHI (nonlockdown; red), and moderate lockdown (purple) intervention effect schedules are described. Moderate PHI (nonlockdown) and strengthened PHI (nonlockdown) do not contain a lockdown but continue throughout the entire prediction period. Effects of interventions are drawn from relative reductions in the time-varying effective reproduction number (R_t) in Maharashtra from 14 April to 7 June 2021 (for moderate lockdown) and 28 March to 13 April 2021 (for strengthened PHI (nonlockdown). In addition, moderate PHI (nonlockdown) effect was estimated by reducing the effect of strengthened PHI (nonlockdown) effect by 20%. The intervention effect schedules are then LOESS-smoothed (span = 1) to account for day-to-day variations in R_t. Three intervention start dates are depicted: moderate PHI (nonlockdown; orange) measures on 19 February (when the trailing 7-day average R_t first crossed 1), strengthened PHI (nonlockdown; red) measures on 13 March (7-day $R_t > 1.2$), and moderate lockdown (solid purple) measures on 19 March (7-day $R_t > 1.4$). Delayed moderate lockdowns on 30 March (dashed purple) and 15 April (dotted purple) are also shown. The moderate CFR schedule is derived from the daily 14-day case lagged CFR in India (i.e., $CFR_t = \frac{deaths_t}{cases_{t-14}}$), which was then LOESS-smoothed using span = 0.3. The daily death estimates represent the estimated daily case count multiplied by the respective CFR schedule. Peak daily case and death counts under each intervention scenario are shown.

Table 1. Predicted total case counts, cases averted, and percentage reduction with corresponding 95% CI under different lockdown interventions (in millions). Each cell reports (i) the total number of observed cases since the start of lockdown in the first row, (ii) the total number of predicted cases since the start of lockdown in the second row (with 95% CI), (iii) the number of cases averted (relative to observed) since the start of lockdown in the third row (with 95% CI), and (iv) the relative reduction in cases (as a percent) under lockdown in the fourth row (with 95% CI) from the intervention start date to the evaluation date. Cells that are bolded represent a statistically significant reduction in the number of cases under intervention at the 95% CI level. Cells that are italicized are referenced in the text. Numbers are reported in millions.

Evaluation		Moderate PHI (nonlockdown) start date	Strengthened PHI (nonlockdown) start date	Moderate lockdown start date		
Date	Metrics	19 February	13 March	19 March	30 March	15 April
30 March 2021	*Observed*	1.2	0.8	0.6		
	Predicted	0.5 [0.0, 3.2]	0.2 [0.0, 1.9]	0.2 [0.0, 1.5]		
	Averted	0.7 [−2.0, 1.2]	0.6 [−1.1, 0.8]	0.4 [−1.0, 0.6]	–	–
	% Reduction	60.6% [−173.6%, 100.0%]	72.6% [−136.4%, 100.0%]	68.7% [−160.8%, 100.0%]		
15 April 2021	*Observed*	3.3	**2.9**	**2.7**	2.1	
	Predicted	0.7 [0.0, 4.3]	**0.4 [0.0, 2.7]**	**0.4 [0.0, 2.5]**	0.7 [0.0, 2.7]	
	Averted	2.6 [−0.9, 3.3]	**2.5 [0.2, 2.9]**	**2.3 [0.2, 2.7]**	1.4 [−0.5, 2.1]	–
	% Reduction	79.7% [−28.4%, 100.0%]	**86.9% [6.8%, 100.0%]**	**85.4% [7.0%, 100.0%]**	66.7% [−24.1%, 100.0%]	
30 April 2021	*Observed*	**8.2**	**7.8**	**7.6**	**7.0**	4.9
	Predicted	**0.9 [0.0, 5.4]**	**0.5 [0.0, 3.5]**	**0.5 [0.0, 3.3]**	**1.3 [0.0, 4.3]**	2.7 [0.2, 5.6]
	Averted	**7.3 [2.8, 8.2]**	**7.3 [4.3, 7.8]**	**7.1 [4.3, 7.6]**	**5.7 [2.7, 7.0]**	2.2 [−0.7, 4.7]
	% Reduction	**89.3% [34.0%, 100.0%]**	**93.5% [55.5%, 100.0%]**	**92.8% [56.8%, 100.0%]**	**82.0% [38.3%, 100.0%]**	45.3% [−15.1%, 96.3%]
15 May 2021	*Observed*	**13.7**	**13.3**	**13.1**	**12.5**	10.4
	Predicted	**1.1 [0.0, 6.7]**	**0.6 [0.0, 4.1]**	**0.7 [0.0, 3.9]**	**1.7 [0.0, 5.7]**	5.4 [1.0, 10.9]
	Averted	**12.6 [7.0, 13.7]**	**12.7 [9.2, 13.3]**	**12.5 [9.2, 13.1]**	**10.8 [6.8, 12.5]**	5.0 [−0.5, 9.4]
	% Reduction	**92.0% [50.8%, 100.0%]**	**95.4% [69.3%, 100.0%]**	**94.9% [70.1%, 100.0%]**	**86.3% [54.3%, 100.0%]**	47.6% [−4.5%, 90.0%]
30 May 2021	*Observed*	**17.1**	**16.7**	**16.5**	**15.9**	13.8
	Predicted	**1.3 [0.0, 8.2]**	**0.7 [0.0, 4.7]**	**0.8 [0.0, 4.5]**	**2.1 [0.0, 7.0]**	8.3 [2.0, 16.6]
	Averted	**15.7 [8.8, 17.1]**	**16.0 [12.0, 16.7]**	**15.7 [12.0, 16.5]**	**13.8 [8.9, 15.9]**	5.5 [−2.8, 11.8]
	% Reduction	**92.2% [51.7%, 100.0%]**	**95.7% [72.0%, 100.0%]**	**95.3% [72.8%, 100.0%]**	**86.6% [55.8%, 100.0%]**	39.8% [−20.5%, 85.6%]
15 June 2021	*Observed*	***18.7***	***18.3***	***18.1***	***17.5***	*15.3*
	Predicted	***1.6 [0.0, 10.2]***	***0.8 [0.0, 5.3]***	***0.9 [0.0, 5.1]***	***2.6 [0.0, 8.7]***	*11.5 [2.9, 23.9]*
	Averted	***17.0 [8.5, 18.7]***	***17.5 [13.0, 18.3]***	***17.2 [13.0, 18.1]***	***14.9 [8.8, 17.5]***	*3.9 [−8.5, 12.4]*
	% Reduction	***91.3% [45.3%, 100.0%]***	***95.5% [71.2%, 100.0%]***	***95.0% [71.7%, 100.0%]***	***85.3% [50.5%, 100.0%]***	*25.2% [−55.6%, 81.0%]*

of the intervention matters, and 19 to 30 March would have been an effective time window for intervening. A moderate lockdown beginning on 15 April would likely have been too late to lead to a reduction in case counts relative to what was observed [3.9 million cases (95% CI: [−8.5, 12.4]) averted through 15 June, a 25.2% (95% CI: [−55.6%, 81.0%]) relative reduction; italicized in Table 1]. One can note considerable uncertainty in all the accompanying CIs.

Intervention effect on deaths

To estimate the number of preventable deaths, we multiply the predicted number of cases under each intervention effect with the daily case fatality rates (CFRs) estimated under three different scenarios. Three daily CFR schedules, based on observed data from Kerala, India, and Maharashtra (Fig. 2E), are applied to daily predicted case counts. For simplicity, we refer to these as low-, moderate-, and high-CFR schedules, each representing the three CFR tertiles in India (table S4), respectively. Details are presented in section S2.1.1. Results here are presented for the moderate-CFR schedule. Numerical results for the low-CFR (table S5) and high-CFR (table S6)–based schedules and an accompanying figure [fig. S5 (A and B, respectively)] can be found in section S2.2.

A similar pattern to the case counts can be seen with respect to death counts. Early and sustained implementation of moderate PHI (nonlockdown) could have avoided 203.2 (95% CI: [93.9, 223.4])

thousand deaths [a 91.0% (95% CI: [42.0%, 100.0%]) reduction] from 19 February to 15 June 2021 (of the 223.4 thousand deaths observed during this period; italicized in Table 2), without having to institute a lockdown. Strengthened PHI (nonlockdown) beginning 13 March 2021 avoids 210.5 (95% CI: [151.7, 221.0]) thousand deaths through 15 June, representing a 95.3% (95% CI: [68.7%, 100.0%]; italicized in Table 2) reduction.

Clear benefits from lockdowns beginning in mid- and late-March are present under all CFR scenarios through mid-June (Fig. 3B). Under the moderate lockdown effect with moderate-CFR schedule, starting on 19 March, our estimates show that 208.2 thousand deaths [95% CI: 151.4, 220.0] could have been avoided by 15 June 2021 (italicized in Table 2), a reduction of 94.6% (95% CI: [68.8%, 100.0%]) from the 220 thousand deaths observed from 19 March to 15 June 2021. This benefit is reduced with a 30 March moderate lockdown, averting 182.0 (95% CI: [99.7, 217.1]) thousand deaths [an 83.8% (95% CI: [45.9%, 100.0%]) reduction; italicized in Table 2]. A moderate lockdown on 15 April would have avoided 51.6 (95% CI: −110.8, 166.2) thousand deaths (25.2% [95% CI: −54.0%, 81.0%]; italicized in Table 2), but this is not significant and suggests

Table 2. Predicted total death counts, deaths averted, and percentage reduction with corresponding 95% CI under different lockdown interventions and moderate CFR (in thousands). Each cell reports (i) the total number of observed deaths since the start of intervention in the first row, (ii) the total number of predicted deaths since the start of intervention in the second row (with 95% CI), (iii) the number of deaths averted (relative to observed) since the start of intervention in the third row (with 95% CI), and (iv) the relative reduction in cases (as a percent) under lockdown in the fourth row (with 95% CI) from the intervention start date through the evaluation date. Cells that are bolded represent a statistically significant reduction in the number of cases under intervention at the 95% CI level. Cells that are italicized are referenced in the text. Numbers are reported in thousands.

**Evaluation**		**Moderate PHI (nonlockdown) start date**	**Strengthened PHI (nonlockdown) start date**	**Moderate lockdown start date**		
Date	Metrics	19 February	13 March	19 March	30 March	15 April
30 March 2021	*Observed*	6.3	3.9	2.9		
	Predicted	4.5 [0.0, 31.2]	2.3 [0.0, 20.2]	2.1 [0.0, 17.8]		
	Averted	1.8 [−25.0, 6.3]	1.5 [−16.4, 3.9]	0.8 [−14.9, 2.9]	–	–
	% Reduction	28.5% [−399.4%, 100.0%]	39.4% [−424.3%, 100.0%]	27.2% [−511.1%, 100.0%]		
15 April 2021	*Observed*	18.1	15.7	14.7	11.8	
	Predicted	7.4 [0.0, 47.1]	4.6 [0.0, 33.6]	5.0 [0.0, 32.4]	9.7 [0.0, 36.2]	
	Averted	10.7 [−29.0, 18.1]	11.1 [−17.9, 15.7]	9.7 [−17.7, 14.7]	2.1 [−24.4, 11.8]	–
	% Reduction	59.3% [−160.6%, 100.0%]	70.5% [−114.0%, 100.0%]	66.0% [−120.1%, 100.0%]	17.8% [−205.8%, 100.0%]	
30 April 2021	*Observed*	55.6	**53.2**	**52.2**	49.3	37.5
	Predicted	10.7 [0.0, 68.1]	**6.7 [0.0, 47.3]**	**7.5 [0.0, 46.0]**	18.7 [0.0, 64.7]	43.4 [3.0, 91.3]
	Averted	44.9 [−12.5, 55.6]	**46.5 [5.9, 53.2]**	**44.8 [6.2, 52.2]**	30.7 [−15.4, 49.3]	−5.9 [−53.8, 34.5]
	% Reduction	80.8% [−22.4%, 100.0%]	**87.4% [11.1%, 100.0%]**	**85.7% [11.9%, 100.0%]**	62.2% [−31.2%, 100.0%]	−15.7% [−143.4%, 92.1%]
15 May 2021	*Observed*	**114.1**	**111.7**	**110.7**	**107.8**	96.0
	Predicted	**13.4 [0.0, 84.4]**	**8.0 [0.0, 54.4]**	**8.9 [0.0, 53.7]**	**24.3 [0.0, 81.8]**	77.8 [14.7, 154.7]
	Averted	**100.6 [29.7, 114.1]**	**103.7 [57.3, 111.7]**	**101.8 [57.1, 110.7]**	**83.5 [26.1, 107.8]**	18.2 [−58.8, 81.3]
	% Reduction	**88.2% [26.0%, 100.0%]**	**92.8% [51.3%, 100.0%]**	**91.9% [51.5%, 100.0%]**	**77.4% [24.2%, 100.0%]**	18.9% [−61.2%, 84.7%]
30 May 2021	*Observed*	**172.9**	**170.5**	**169.5**	**166.6**	154.8
	Predicted	**16.0 [0.0, 100.0]**	**9.1 [0.0, 60.2]**	**10.1 [0.0, 59.5]**	**28.7 [0.0, 94.6]**	108.0 [25.4, 213.2]
	Averted	**156.9 [72.9, 172.9]**	**161.4 [110.3, 170.5]**	**159.4 [110.0, 169.5]**	**137.9 [72.1, 166.6]**	46.8 [−58.4, 129.3]
	% Reduction	**90.8% [42.2%, 100.0%]**	**94.7% [64.7%, 100.0%]**	**94.0% [64.9%, 100.0%]**	**82.8% [43.2%, 100.0%]**	30.2% [−37.8%, 83.6%]
15 June 2021	*Observed*	***223.4***	***221.0***	***220.0***	***217.1***	*205.3*
	Predicted	***20.1 [0.0, 129.4]***	***10.5 [0.0, 69.3]***	***11.8 [0.0, 68.6]***	***35.1 [0.0, 117.4]***	*153.6 [39.1, 316.1]*
	Averted	***203.2 [93.9, 223.4]***	***210.5 [151.7, 221.0]***	***208.2 [151.4, 220.0]***	***182.0 [99.7, 217.1]***	*51.6 [−110.8, 166.2]*
	% Reduction	***91.0% [42.0%, 100.0%]***	***95.3% [68.7%, 100.0%]***	***94.6% [68.8%, 100.0%]***	***83.8% [45.9%, 100.0%]***	*25.2% [−54.0%, 81.0%]*

that a moderate lockdown beginning on 15 April would have been too late to save lives.

Case (table S7) and death (table S8) analyses conducted using an intervention schedule corresponding to the 2020 national lockdown in India (i.e., "strong lockdown") are presented in section S2.3. Results from a model incorporating waning immunity are presented in section S2.4 (fig. S6). Because the results using a model with and without waning immunity are nearly indistinguishable, we present the results without waning immunity here for simplicity.

Sensitivity analyses

We summarize our sensitivity analysis on the effects of "unlocking" and of varying lengths of lockdown effects in section S2.5. While the assumed moderate and strengthened PHI (nonlockdown) scenarios continue through the end of the prediction period, 30 June 2021, the moderate lockdown is lifted 55 days after the start date (as was observed in Maharashtra from 14 April to 7 June 2021). The unlocking analysis considers unlocking the moderate lockdown after 55 days on the same start dates as above but extends the prediction horizon through the end of July 2021, where we see continued control if it was instituted early (fig. S7). The length of lockdown (considering 4-, 6-, and 8-week moderate lockdown scenarios with 13 March, 19 March, and 15 April start dates) appears less important than the timing of the start of the lockdown (fig. S8). Meaningful reductions in cases are seen when extending a lockdown from 4 to 6 weeks but become less apparent after extending from 6 to 8 weeks.

Last, to assess whether our estimates depend on the type of transmission model used, we conducted the same analyses presented here using a version of the susceptible-exposed-infected-recovered (SEIR) model with intervention effects (*42*), which allows for time-varying transmission rates β_t and accounts for asymptomatic/untested individuals (schematic presented in fig. S9). In general, the intervention effects on cases (fig. S10) are attenuated relative to the eSAIR-based results. For example, for a moderate lockdown beginning on 19 March using the SEIR model, we estimate that 13.1 (95% CI: [13.0, 13.2]) million cases would be averted through 15 June 2021 [a 79.2% (95% CI: [78.5%, 79.7%]) reduction; table S9]. While the point estimate for cases averted is attenuated relative to the eSAIR result (95.0%), the SEIR estimate and its 95% CI are contained within the eSAIR CI (95% CI: [71.7%, 100.0%]). The two models broadly agree regarding the effect of early interventions, but their intervals become non-overlapping for interventions starting on later dates. For example, with the moderate lockdown on 30 March, through 15 June, 85.3% (95% CI: [50.5%, 100.0%]) of cases are averted according to eSAIR versus 46.0% (95% CI: [44.7%, 46.7%]) of cases that are averted according to SEIR model. The SEIR model finds the lockdowns on 30 March or 15 April to have less benefit than the corresponding eSAIR results. The SEIR model has a more deterministic structure leading to narrow confidence intervals that may not be reflective of the true uncertainty in the predictions.

One feature of the SEIR model that we used is that it can directly estimate deaths as a part of the transmission dynamics (fig. S11). For a moderate lockdown beginning on 19 March, the model estimates that 183.9 (95% CI: [182.8, 184.9]) thousand deaths would be avoided through 15 June 2021 [an 83.6% (95% CI: [83.1%, 84.0%]) reduction; table S10]. We have also considered CFR-based death estimates using the same strategy that we used to estimate eSAIR-based death projections (i.e., SEIR-predicted daily cases multiplied by a daily CFR schedule; fig. S12). These CFR-based estimates (high-, moderate-, and low-CFR–based results in tables S11 to S13, respectively) for a moderate lockdown starting on 19 March result in a smaller reduction in deaths [73.6% (95% CI: [72.7%, 74.3%]); table S12] compared to the direct model-based estimates.

The SEIR-based length of lockdown analysis (fig. S13) supports the conclusion that the length of lockdown is less important than the timing of the start of lockdown. The SEIR results qualitatively support our conclusions from the eSAIR model and are presented in section S3.

Summary takeaways

Had moderate PHI (nonlockdown) action taken place in February 2021 at the first signs of an uptick (i.e., trailing 7-day average $R_t > 1$) in case counts, 91.3% (95% CI: [45.3%, 100.0%]) of cases and 91.0% (95% CI: [42.0%, 100.0%]) of deaths (of the 18.7 million reported cases and 223.4 thousand reported deaths from 19 February to 15 June 2021) could have been prevented. A moderate lockdown beginning on 19 March 2021 could have prevented 95.0% (95% CI: [71.7%, 100.0%]) of cases and 94.6% (95% CI: [68.8%, 100.0%]) of deaths (of the 18.1 million reported cases and 220.0 thousand reported deaths from 19 March to 15 June 2021). We highlight the considerable uncertainty in the model predictions but note that even if we consider the lower limit of the CIs, more than 42% of reported cases and deaths could have been prevented.

Reduction in undetected cases and deaths

If one considers an underreporting factor of 25 to 30 for cases based on the fourth national serosurvey (*43*) and an underreporting factor of 7 to 8 for deaths based on excess death calculations (*44*), then the estimated reductions in absolute number of infections and deaths are even more staggering. The estimates for underreporting factors on 19 March 2021 for cases and deaths by the SEIR model during the trailing 100-day analytic period are 42.7 (95% CI: [41.6, 43.9]) and 8.34 (95% CI: [8.2, 8.5]), respectively. A SEIR model–based estimate of the reduction in total number of infections and deaths with a 19 March moderate lockdown intervention versus no intervention leads to an estimated 69% reduction in total infections and 64% reduction in total deaths. Since these numbers cannot be compared with observed data, we refrain from expanding upon these latent metrics.

Impact of PHI on hospital and intensive care unit bed capacity

Because a goal of PHI is to keep the number of COVID-19 cases within the capacity of the health system, we conducted an analysis (section S4) to understand whether PHI could have prevented the health system capacity from being overwhelmed in India's second COVID-19 wave. According to recent estimates, there are approximately 1.9 million public and private hospital beds and 95 thousand intensive care unit (ICU) beds in India (*45*). Using data from covid-19india.org (*16*), we calculate the observed daily active cases by taking the difference in cumulative confirmed cases from cumulative recovered cases in India. We further estimate daily active cases under early interventions by using the eSAIR model. Assuming that approximately 7.8% of COVID-19–positive cases will be hospitalized (*46*) and, of those, roughly 11% will require ICU care (*47*), we show that early nonlockdown and early lockdown interventions would prevent a surge in excess of India's current hospital and ICU bed capacity (per 10,000 population; fig. S14). However, if

implementation of moderate lockdown was delayed until 15 April 2021, hospital bed and ICU capacity would have been greatly exceeded, as indicated by the shaded red area under the dotted purple line in fig. S14 (supported by the SEIR-based analysis in fig. S15). A lockdown on 30 March would also have stretched capacity limits.

Building a data-driven framework for a pandemic-resilient future

Timing and choice of interventions

We used our modeling analyses to develop a data-driven framework to inform future approaches to deploying PHI for pandemic control in India that may be relevant for low- and middle-income countries (LMICs; Table 3). This framework is informed by the ones from WHO and other countries, particularly Ontario, Canada (*48*–*51*); however, we contextualize our recommendations for India. Details of framework development are described in section S5. Our framework includes (i) indicators to guide PHI escalations, (ii) health system preparations in anticipation of the next PHI tier, (iii) communication to the public for each tier, (iv) PHI that might be implemented in each tier, and (v) social protections that should be implemented to mitigate the adverse social and economic impacts of PHI.

The PHIs recommended within each tier broadly align with three intervention effects in our modeling analyses: moderate PHI (nonlockdown) (orange), strengthened PHI (nonlockdown) (red), and moderate lockdown (purple) map to tiers 2, 3, and 4, respectively (Table 3). We additionally demonstrated how real-life PHI implemented in Maharashtra informed the framework recommendations using the strengthened PHI (nonlockdown) scenario as an example (tables S3 and S14). Indicator thresholds that should trigger escalation to the next PHI tier are informed by epidemiological metrics (e.g., R_t, test-positive rate) observed on dates when we implemented intervention effects in our modeling analyses. Intervention effects implemented on these dates were effective in mitigating COVID-19 cases and deaths and therefore might represent critical time points for intervention that could mitigate future waves. Unlike other PHI frameworks, our framework recommends actions to anticipate potential escalation to the next PHI tier and addresses the importance of social protections, informed by schemes available in India.

More than 2 years into the pandemic, with about 60% of India's population fully vaccinated as of April 2022 (*52*), this framework remains highly relevant given the uncertainty regarding future SARS-CoV-2 variants that may emerge. The global community has already had to contend with VOCs with increased transmissibility (e.g., Alpha, Delta, and Omicron), increased clinical severity [e.g., Delta (*53*)], and escape from both vaccine-acquired and naturally acquired immunity [e.g., Omicron (*54*)]. The unpredictability of future variants is increased by the fact that the Omicron VOC appears to have evolved along a different evolutionary branch than previously prevalent VOCs, such as Delta (*55*). Hence, a reasonable possibility remains of a VOC emerging that has immune escape, increased transmissibility, and greater clinical severity. If such a VOC emerges, then LMICs need frameworks such as the one we have outlined to guide the escalation of PHI and social protections in a manner that could reduce mortality and prevent the health system from being overwhelmed while mitigating the adverse effects of PHI.

Need for better data and smart surveillance

Our findings highlight a need for enhanced surveillance efforts in the future to guide public health measures in India. Early detection of new VOCs will be critical for the foreseeable future. The use of emerging technologies, such as miSHERLOCK (*56*), that have point-of-care and direct-to-consumer rapid antigen tests that have variant detection assays can also assist in the detection of emerging variants. SARS-CoV-2 viral RNA load, including specific variants, can be detected in sewage wastewater (*57*). A wastewater-based epidemiology warning system would be beneficial for early detection of surges and new variants (*58*).

Modeling epidemiologic characteristics of the VOCs has been challenging in India because of limited sequencing data available nationally. As of 12 April 2022, INSACOG has sequenced 129,141 SARS-CoV-2 genomes (*59*). Two critical gaps in genomic sequencing in India are a lack of representative sampling of urban and rural areas and stratification by epidemiological metadata such as age, gender, comorbidities (such as diabetes or immunosuppressing illnesses), and vaccination status and date.

In addition to these genomic surveillance recommendations, there is a need for improved surveillance data to assist in characterizing outbreaks in real time and informing timely decision-making. We summarize key data and information needs in table S15.

DISCUSSION

We saw that despite developments in COVID-19 treatments, care, and vaccines and a slightly lower infection fatality rate in the second wave (*60*), a staggering number of deaths happened during this period because of uncontrolled transmission of the virus. India's second wave represents a case study in uncontrolled transmission in a largely unvaccinated population. Our analysis shows that a large fraction of cases and deaths could potentially be averted with early nonlockdown interventions, thereby avoiding the need for lockdowns altogether.

There are several new and important findings in this work. We estimated PHI effects from analyzing empirical data. In addition, we translated the modeling results into a practical, tiered PHI framework that considers social protections already available in India. Our finding that nonlockdown PHIs are effective and sufficient is important because lockdowns are associated with potential health, economic, and social costs (*61*). During India's nationwide lockdown in 2020, most forms of public transportation were shut down, inadvertently resulting in substantial drops in care seeking for tuberculosis treatment (*14*), HIV testing (*13*), and child vaccination (*15*). Vulnerable populations such as migrants were disproportionately affected by unemployment and food insecurity in major cities, leading to millions walking long distances, back to rural areas.

Should lockdowns need to be instituted, the similar COVID-19 outcomes achieved in our model, regardless of whether a strong lockdown (such as the 2020 national lockdown) or a moderate lockdown (such as the 2021 lockdown in Maharashtra) was implemented nationwide in March 2021, indicate that less stringent forms of lockdown can be effective if implemented soon after cases start to rise. Our findings also suggest that if lockdowns are instituted earlier, then they could potentially be implemented for a shorter period, thereby limiting the duration of adverse health, economic, and social impact.

Although lockdowns have potential harms, these must be weighed against the health, economic, and social harms caused by uncontrolled transmission (*62*). For example, while care seeking may be challenging during lockdowns, during periods of uncontrolled transmission, accessing care may be more challenging or even impossible because of fear of getting infected or because the health system is overwhelmed.

Table 3. A tiered COVID-19 response framework for PHIs in India, with relevance to other low- and lower-middle–income countries with suboptimal population immunity to COVID-19. This framework is adapted from multiple frameworks, including the considerations for implementing and adjusting public health and social measures in the context of COVID-19 by WHO (*48*), Keeping Ontario Safe and Open COVID-19 response framework (*49*), Scotland's route map in and out of the crisis (*50*), and the Singapore Ministry of Health Pandemic Readiness and Response Plans for Influenza and Other Acute Respiratory Diseases (*51*). The recommendations at each of the tiers are meant to highlight general principles and provide illustrative examples of how PHIs and social protections could be concurrently escalated using surveillance indicators. PCR, polymerase chain reaction; PDS, public distribution system.

	Tier 1 (green): Prevent (standard/baseline measures)	Tier 2 (orange): Protect (moderate measures)	Tier 3 (red): Restrict (strengthened measures)	Tier 4 (purple): Humane shelter at home (maximum measures)
**Corresponding intervention effect in model**	None (represents optimal baseline behavior)	Moderate PHI (nonlockdown) effect	Strengthened PHI (nonlockdown) effect	Moderate lockdown effect
**Approximate dates informing indicator thresholds**	December 2020 to January 2021 (before the second wave)	19 February 2021 (start of the second wave)	13 March 2021	19 March 2021
**Indicators**	**Epidemiology**	**Epidemiology**	**Epidemiology**	**Epidemiology**
	7-day average R_t is decreasing or stable and <1.0 nationally and in large gateway states like Maharashtra	7-day average R_t is increasing for 7 days and is above 1.0 nationally or in large gateway states like Maharashtra	7-day average R_t is above 1.2 nationally or in multiple states	7-day average R_t is above 1.4 nationally or in multiple states
	Test positivity, <2%	Test positivity, 2 to 5%	Test positivity, 5 to 10%	Test positivity, 10%
	No outbreak trends	Increasing outbreaks in multiple settings and cluster infections indicating emergence of new virus strains	Increasing number of larger outbreaks, new variants circulating, cluster infections increasing	Increasing case incidence or test positivity
	Community transmission/ unlinked cases stable	Community transmission/ unlinked cases increasing	Community transmission/ unlinked cases increasing	Genomic surveillance indicating a new strain becoming dominating rapidly
	**Health system capacity**	**Health system capacity**	**Health system capacity**	**Health system capacity**
	Hospital capacity adequate	Hospital capacity adequate	Hospital occupancy increasing	Hospital capacity at risk of being overwhelmed
	Adequate case and contact follow-up within 24 hours	Adequate case and contact follow-up within 24 hours	Public health unit capacity for case and contact tracing at risk	Public health unit capacity for case and contact tracing overwhelmed
**Communication to public**		**Advice from previous level**	**Advice from previous levels**	**Advice from previous levels**
	Physical distancing when possible	Social, political, and religious gatherings should be limited to small groups	Families should not visit any other household or allow visitors into their homes	Trips outside of the home to other indoor settings should only be for essential reasons (groceries, pharmacy, health care, and assisting vulnerable individuals)
	Wear masks in all indoor settings, public transportation, and selected outdoor settings where physical distancing cannot be maintained	Avoid travel within and between states unless for work or essential purposes	Avoid social, political, and religious gatherings	Noncongregate physical activity and exercise outdoors encouraged
	Seek testing if symptomatic or exposed, even if asymptomatic	Noncongregate physical activity and exercise outdoors encouraged	Work remotely if possible	
	Get vaccinated		Noncongregate physical activity and exercise outdoors encouraged	

continued on next page

		Restrictions from previous level	*Restrictions from previous level*	*Restrictions from previous level*
**Public health interventions**	**Higher-risk settings**	**Higher risk settings**	**Higher risk settings**	**Higher risk settings**
	Restaurants, bars, coffee/tea shops, and gyms	*Restaurants, bars, coffee/tea shops, and gyms*	*Restaurants, bars, coffee/tea shops, and gyms*	*Restaurants, bars, coffee/tea shops, and gyms*
	Physical distancing and masks in indoor settings except when eating, drinking, or exercising	Additional capacity limits in indoor settings	Closure of indoor dining, but food pickup and delivery are allowed	Closure of indoor dining, but food pickup and delivery are allowed
	Cinemas and performance venues	*Cinemas and performance venues*	*Cinemas and performance venues*	*Cinemas and performance venues*
	Masks in indoor settings and reduced capacity to maintain physical distancing	Additional capacity limits in indoor settings	Closure of indoor venues	Closure of indoor venues
	Weddings and social, political, and religious gatherings	*Weddings and social, political, and religious gatherings*	*Weddings and social, political, and religious gatherings*	*Weddings and social, political, and religious gatherings*
	Masks in indoor settings and restrictions on mass gatherings	Additional capacity limits in indoor settings	Stringent indoor capacity limits, e.g., not more than 5 to 10 people	Cancellation of all indoor events
		Outdoor establishments would be exempted from capacity limits, with the exception of mass gatherings	Outdoor establishments or gatherings would also have capacity limits, although less stringent	Outdoor gatherings should be limited to individuals in the same family unit
	**Lower-risk settings**	**Lower-risk settings**	**Lower-risk settings**	**Lower-risk settings**
	Workplaces	*Workplaces*	*Workplaces*	*Workplaces*
	Masks in indoor settings and avoidance of communal dining, unless outdoors	No additional restrictions	Institution of indoor capacity limits	Closure of nonessential workplaces
	Retail stores	*Retail stores*	*Retail stores*	*Retail stores*
	Masks in indoor settings	No additional restrictions	Institution of indoor capacity limits	Closure of nonessential stores
	Transportation (trains, buses, and autorickshaws)	*Transportation (trains, buses, and autorickshaws)*	*Transportation (trains, buses, and autorickshaws)*	*Transportation (trains, buses, and autorickshaws)*
	Masks in all transportation	No additional restrictions	Institution of capacity limits	Capacity limits continue, but public and private transportation continue to operate to facilitate access to essential services
	Household	*Household*	*Household*	*Household*
	Domestic workers may continue working for all purposes. Employers must provide access to masks and other personal protective equipment	No additional restrictions	Reduced frequency of visits for non–live-in domestic workers for nonessential tasks. Employers encouraged to provide full salary support to affected domestic workers	Domestic workers limited to essential caregiving roles (e.g., health assistants, home nurses, and elder care)<br>Live-in domestic workers are allowed, as they are considered part of the family unit

continued on next page

		Preparations from previous level	***Preparations from previous level***	
	**Surveillance**	**Surveillance**	**Surveillance**	
	Ongoing molecular surveillance to identify novel variants; Develop protocols for wide-scale community-based antigen or PCR testing	Initiate active surveillance strategies using representative sampling and door-to-door testing	Implement wide-scale community-based antigen or PCR testing, particularly in location with identified clusters	
	**Care delivery**	**Care delivery**	**Care delivery**	
**Public health preparations for the next tier**	Update COVID-19 treatment and prevention protocols based on the latest evidence	Establish call centers and Web-based platforms to assist with accessing COVID-19 testing and treatment services	Mobilize oxygen, ventilators, and other supplies to lower-tier facilities in anticipation of higher-tiered facilities being overwhelmed	***Ongoing implementation of surveillance and care delivery strategies from prior levels***
		Intensified field-based COVID-19 testing, contact tracing, isolation, and quarantine	Implement home-based vaccine delivery to avert drops in vaccine uptake	
	Develop protocols for virtual-, home-, or community-based care for other medical conditions (e.g., tuberculosis, HIV, and diabetes)	Establish networks of lower-tier health facilities that can be repurposed to provide COVID-19 care in case existing hospitals get overwhelmed; Expand the cadre of health care workers with training in COVID-19 management	Implement virtual-, home-, or community-based care for other medical conditions (e.g., tuberculosis, HIV, and diabetes)	
**Social protections**	Central and state governments should strengthen existing safety net programs in anticipation of challenges that could be faced across society. Such programs may include the PDS, the Mahatma Gandhi Rural Employment Guarantee Scheme, pension schemes, and public sector health insurance programs.		Elimination of restrictions to accessing the PDS to ensure universal accessibility for vulnerable populations, such as migrant workers	Elimination of restrictions to accessing the PDS to ensure universal accessibility for vulnerable populations, such as migrant workers
			Enhanced diversity of food distributed through the PDS	Enhanced diversity of food distributed through the PDS
			Targeted financial relief measures to affected businesses	Cash transfers to large sections of the population, particularly vulnerable populations; Wide-scale financial relief package to support the economy

As outlined in our framework, the adverse impacts of PHI can be mitigated by reimagining "lockdowns" as "humane shelter at home," a term that we have previously suggested to emphasize the critical importance of concurrent social protections (*61*). Health care can remain accessible by ensuring that public transportation is allowed to continue, with masking and capacity limits in place. Continuity in health services could be ensured using unconventional approaches, such as home delivery of medications for chronic diseases. Food insecurity could be ameliorated by improving access to India's public distribution system (PDS). As PHIs are escalated, the PDS could be made universally accessible by dropping requirements to present a ration card to access food.

PHIs are not a permanent solution for pandemic control. Instead, the PHIs modeled here are aimed at slowing the growth of cases to prevent the health system from being overwhelmed while buying time to achieve other goals including accelerating vaccine and booster rollout and further expanding testing, contact tracing, and treatment capacity to maximize the interval until escalation of PHI is needed again.

Considering these goals, we suggest tiered approaches to lifting restrictions to blunt rapid rises in case counts that could occur with the sudden removal of all restrictions. As the 2020 nationwide lockdown was relaxed, India used one such tiered approach (*63*); tiered relaxation of PHI could be guided by achieving sustained low values of key indicators (e.g., R_t, test-positive rate), as well as vaccination coverage rates.

Our quantitative analyses are based on intervention effect schedules derived from observed data in Maharashtra during the second

wave in 2021. However, the estimates and their subsequent conclusions are qualitatively consistent with the literature (table S16). Empirical analyses into the relative effects of nonpharmaceutical interventions (NPIs) on R_t in 190 countries have found meaningful reductions [e.g., from −9.26% [95% CI: −11.46%, −7.01%] due to travel restriction to as much as −42.94% [95% CI: −44.24%, −41.60%] due to social distancing (*64*)]. Here, we estimate maximum relative reductions in R_t ranging from 17% in the moderate PHI scenario to 41% in the moderate lockdown scenario. Pei and colleagues (*65*) conducted a counterfactual analysis that an estimated 91.0% (95% CI: 87.1%, 94.0%) of cases and 90.8% (95% CI: 86.0%, 94.5%) of deaths could have been avoided in the United States if control measures had been adopted 2 weeks earlier. Our findings are also consistent with a meta-analysis by Mendez-Brito and colleagues (*66*), which found that "early implementation was associated with a higher effectiveness in reducing COVID-19 cases and deaths, while general stringency of the NPIs was not."

There are several limitations in our work. First, underreporting of cases and deaths attributed to COVID-19 is not aptly accounted for across these results. While we characterize effects of PHI on reported cases, this only captures a small fraction of infections. We do attempt to capture covert infections not only through the eSAIR model using seroprevalence surveys in India (*17*, *43*) but also through latent E compartment in the SEIR model. Recent excess death studies indicate an underreporting factor of 7 to 8 for deaths (*24*, *25*, *44*). Excess mortality is a metric defined as the net difference between the observed all-cause mortality during this time and the all-cause mortality predicted on the basis of historical trends (*25*). The metric captures overall pandemic-related deaths (including those not directly attributable to COVID-19) that may fall in four large buckets: (i) reported COVID-19 deaths, (ii) unreported COVID-19–related deaths, (iii) pandemic-related (non–COVID-19) deaths, and (iv) deaths due to long-term COVID-19 effects (fig. S16). PHI aimed at reducing COVID-19 transmission may reduce (i), (ii), and (iv), but their effects on (iii) are not obvious. For example, delays in and barriers to health care access, loneliness, and substance abuse may increase (iii), but reduction in mobility and increased hygiene practices can reduce road accidents (accounts for 2.5% of deaths in India) and diarrheal (accounts for 7.5% of deaths in India) and other infectious respiratory diseases (accounts for 9.1% of deaths in India) (*67*). Future work disentangling the totality of effects of COVID-19 interventions on non–COVID-19–related deaths is critical.

Second, disaggregated COVID-19 case and death data are not available for India, prohibiting an age-stratified fatality comparison. Third, our models do not incorporate vaccine rollout. During the second wave analysis period, about 4% of India was fully vaccinated, and 15% received at least one dose [based on vaccine data available from covid19india.org (*16*) through 15 June 2021]. Since age and occupation were criteria for vaccine eligibility, accounting for vaccine distribution during this period also requires age-stratified data. Fourth, our assessment period for the effect of intervention contains the period of 30 March through 15 June 2021, where many states instituted partial lockdowns. Thus, the idealized hypothetical lockdown effects are being compared with observed data, capturing a mix of scattered mitigation strategies. The comparison up to 15 April 2021 is clearer to interpret because no significant interventions had yet taken place. Fifth, while we have attempted to capture increased transmissibility as a result of the increased proportion of cases due to the Delta variant (*41*) through the time-varying transmission parameter β_t (fig. S3), our estimates of the variant distribution are based on sequencing data that may not be nationally representative. This analysis would be best served by a true multistrain model (*26*). More broadly, our model makes the important simplifying assumption of homogeneous mixing between populations and various age groups. Significant heterogeneities with respect to the geographical differences (*68*) and transmission patterns (*69*) exist that are ignored.

Last, a potential limitation of our tiered framework is that, since it is informed by data from India's second wave, it may become less relevant with increasing population immunity from vaccines and natural infections. Nevertheless, the general principles in this tiered framework may be applicable to future outbreaks. The tiered framework offers flexibility for modifying or prioritizing the specific epidemiological indicators that should trigger PHI escalation. For example, in a future scenario with a relatively highly vaccinated population and continued vaccine efficacy, increases in R_t or test-positive rate alone may not merit escalation of PHI in the absence of rising hospitalizations. Conversely, a new variant that can evade vaccine-acquired or naturally acquired immunity or is clinically more lethal might merit escalation of PHI at lower indicator thresholds or by monitoring of the rate of cluster infections and hospitalizations. Our tiered framework has immediate relevance to many low-income countries, where vaccination rates remain low at about 14.8% (as of 4 April 2022) (*70*) and where integration of social protections for vulnerable populations will be crucial with any escalation of PHI.

Rapid expansion of vaccination efforts as an effective, evidence-based intervention to reduce COVID-19 mortality is the way moving forward for India, and the country has been highly successful with its vaccine drive after the second wave (*71*). However, even in countries with relatively high vaccination coverage such as the United States and United Kingdom and in the European Union, we are seeing new surges in 2022 (*72*). In addition, new VOCs are likely to emerge in countries with lower vaccination rates (*73*). Thus, India and other LMICs must be prepared to enact PHI quickly and humanely for the foreseeable future. By limiting transmission, such measures are crucial for curbing infections, deaths, and subsequent viral mutation; our analysis demonstrates that the earlier an intervention takes place, the better; timing matters.

We hope that the lessons from this pandemic lead to a bolstering of public health infrastructure including the rapid collection and release of comprehensive data and compel policy-makers to act more proactively and confidently, thereby preparing India to respond to future waves and crises more effectively. We also believe that our analyses and COVID-19 response framework have important implications for outbreak and pandemic control globally, especially in many low-income countries where vaccine rollout remains slow and much of the population remains at risk for future waves of COVID-19.

MATERIALS AND METHODS

We use data on reported cases and COVID-19–attributed reported deaths through 31 July 2021, for our descriptive analysis, and 30 June 2021, for predictive modeling from covid19india.org (*16*). Population data for India were obtained from the Unique Identification Authority of India (Aadhar) (*74*). All scripts used to perform the analysis and the results obtained can be found online at www.doi.org/10.5281/zenodo.6514992 (*75*).

We implement an extended version of the traditional susceptible-antibody-infected-recovered model, called an eSAIR model (*76*).

This model relies on an underlying latent Markov SIR model, where the respective probabilities of being susceptible, infected, and removed at a given time t are given by θ_t^S, θ_t^I, and θ_t^R, respectively. To account for population-level seroprevalence, the eSAIR model further introduces a compartment to accommodate those with antibodies at a given time t, with θ_t^A being the probability of being in said antibody (A) compartment at time t. The modified eSAIR framework is depicted in Fig. 1. The eSAIR framework assumes that the true underlying probabilities of the four compartments follow a latent Markov transition process and that we only observe a fraction of the true infected and removed cases. We assume that the observed proportions of infected and removed cases on day t are denoted by Y_t^I and Y_t^R, respectively, and further note that the true underlying probabilities of the S, A, I, and R compartments always add up to unity, i.e., $\theta_t^S + \theta_t^A + \theta_t^I + \theta_t^R = 1$ for all t. This model can then be described by the following system of differential equations

$$\boldsymbol{f}(\boldsymbol{\theta}_t, \boldsymbol{\beta}_t, \boldsymbol{\pi}(t), \boldsymbol{\alpha}(t), \gamma, \gamma_s):$$

$$\frac{d\theta_t^S}{dt} = -\beta_t \pi(t)\,\theta_t^S \theta_t^I - \alpha(t)\,\theta_t^s + w(t)\,\gamma_s \theta_t^R$$

$$\frac{d\theta_t^I}{dt} = \beta_t \pi(t)\,\theta_t^S \theta_t^I - \gamma\theta_t^I$$

$$\frac{d\theta_t^R}{dt} = -w(t)\,\gamma_s \theta_t^R + \gamma\theta_t^I$$

$$\frac{d\theta_t^A}{dt} = \alpha(t)\,\theta_t^s$$

where $\theta_t = (\theta_t^S, \theta_t^A, \theta_t^I, \theta_t^R)$ is the vector of the underlying population probabilities of the four compartments. Here, $\beta_t > 0$ denotes the time-varying disease transmission rate, $\gamma > 0$ denotes the removal rate, $\gamma_s > 0$ denotes the reinfection rate, $\alpha(t)$ denotes the seroprevalence at time t, and $w(t)$ acts as a time-varying waning immunity modifier of γ_s. Details concerning the specification of these functions are presented at the end of this section and in section S2.1.1.

The basic reproduction number $R_0 ≔ \beta_0/\gamma$ indicates the expected number of cases generated by one infected case in the absence of any intervention and assuming that the whole population is susceptible. At this stage, for the observed infected and removed proportions, we assume a Beta-Dirichlet state-space model, independent conditionally of the underlying process

$$Y_t^I \mid \theta_t, \tau \sim \text{Beta}(\lambda^I \theta_t^I, \lambda^I(1 - \theta_t^I))$$

$$Y_t^R \mid \theta_t, \tau \sim \text{Beta}(\lambda^R \theta_t^R, \lambda^R(1 - \theta_t^R))$$

Furthermore, the Markov process on the latent proportions is built as

$$\theta_t \mid \theta_{t-1}, \tau \sim \text{Dirichlet}(\kappa f(\theta_{t-1}, \beta_{t-1}, \gamma))$$

where the mean of θ_t is modeled as an unknown function of the probability vector from the previous time point, along with the transition parameters; $\tau = (\beta_t, \gamma, \gamma_s, \theta_0^T, \lambda, \kappa)$ denotes the whole set of parameters where λ^I, λ^R, and κ are parameters controlling the variability of the observation and latent process, respectively. The prior choices for all parameters and initial values are presented in fig. S1. The posterior draws from this hierarchical Bayesian formulation are generated via an appropriately designed MCMC sampling scheme described in section S2.1.1. One major advantage of the Bayesian implementation is that uncertainty associated with all parameters and functions of parameters can be quantified by using exact posterior draws without relying on large-scale approximation or delta theorem. The CIs for the prevalence and incidence are computed using the posterior distribution of the latent proportions given the observed infected and removed compartment prevalence. Similar techniques apply to the compartment proportions and transmission parameters such as β_t, γ, and γ_s. We also evaluate the intervention scenarios by constructing metrics such as the number of averted cases/deaths and percentage reduction. CIs for these metrics can also be derived. The details of these calculations are provided in section S2.1.2.

Specifying the A compartment

Movement from the S compartment to the A compartment is controlled by $\alpha(t)$, which is determined by the population-level seroprevalence at time t. Considering national serosurveys conducted serially by the Government of India, we fit a piecewise linear curve to the four seroprevalence estimates from June 2020 to July 2021 (see fig. S2) and find the estimated seroprevalence at time t.More details can be found in section S2.1.1.

Choice of time-varying transmission parameter βt

To account for the presence of different strains with varying transmission rates, we propose a time-varying overall transmission rate by considering the top three dominant strains, namely the ancestral strain, with a transmission rate of $\beta^{(1)}$; the Alpha variant, with a 50% increase in transmissibility given by $\beta^{(2)} = 1.5 \times \beta^{(1)}$ (*77*); and the Delta variant, with a 150% increase in transmissibility, given by $\beta^{(3)} = 2.5 \times \beta^{(1)}$ (*41*). On the basis of INSACOG data (*78*), let the relative prevalence of the ancestral, Alpha, and Delta strains at time t be denoted by $p_1(t)$, $p_2(t)$, and $p_3(t)$, respectively. We combine these quantities together to construct the overall time-varying transmission rate $\beta^*(t) = \sum_{i=1}^{3} p_i(t)\,\beta^{(i)}$ and then apply a LOESS smoother with span of 0.25 to construct a smoothed version of $\beta^*(t)$,namely, β_t, which we use in our analyses (fig. S3). To allow for posterior inference on β_t, instead of supplying a deterministic β_t series to the model, we consider the decomposition $\beta_t = \beta^{(1)} \times s(t)$ and note that the series thus constructed has only one unknown parameter, which may be estimated by the MCMC, the assumed transmission rate of the ancestral variant, $\beta^{(1)}$. The multiplier series $s(t)$ is nonstochastic and is supplied to the model. Using the posterior draws of $\beta^{(1)}$, we can construct a posterior mean and 95% CIs of $\beta^{(1)}$ and, consequently, of time-varying β_t. The initial value for this parameter is derived as the product of the initial basic reproduction number R_0 and the initial rate of removal γ_0, both of which have log-normal priors imposed on them.

Modeling reinfection through waning immunity

Our modification of the eSAIR model further incorporates the possibility of reinfection and waning immunity over time from the R to S compartment by means of a time-varying transmission parameter given by $\gamma_s w(t)$. This reinfection could be from the ancestral strain or the newly emerging variants of interest or VOCs, such as Alpha and Delta. For $w(t)$, we consider several special cases $w(t) = 0\ \forall\ t$ (no waning immunity, reverting to the original eSAIR model), a discrete time-varying vector of proportions, or a continuous function. More details about our choice of $w(t)$ and γ_s based on existing literature are provided in section S2.1.1. In particular, a population-level observational study in Denmark estimated 20.7% of seropositive

individuals to be unprotected against reinfection at 6 months of follow-up and 22.3% for the same estimate at ≥7 months of follow-up (*79*).

Choice of intervention schedule $\pi(t)$

As described in the here, the model incorporates the effect of an intervention on case counts through a modifier schedule $\pi(t)$, which modifies the time-varying disease transmission rate from β_t to $\beta_t\pi(t)$.

SEIR model used in sensitivity analyses

The modified SEIR model used in our sensitivity analyses (section S3) is based on a SEIR model with additional compartments, developed to study the COVID-19 outbreak in India (*42*, *80*). The modifications are described in section S3 and summarized in fig. S9. The modified SEIR model code used in our sensitivity analyses is included in our code repository at www.doi.org/10.5281/zenodo.6514992 (*75*).

SUPPLEMENTARY MATERIALS

Supplementary material for this article is available at https://science.org/doi/10.1126/sciadv.abp8621

REFERENCES AND NOTES

1. Population, total - India: https://data.worldbank.org/indicator/SP.POP.TOTL?locations=IN.
2. M. A. Andrews, B. Areekal, K. R. Rajesh, J. Krishnan, R. Suryakala, B. Krishnan, C. P. Muraly, P. V. Santhosh, First confirmed case of COVID-19 infection in India: A case report. *Indian J. Med. Res.* **151**, 490–492 (2020).
3. COVID-19 India Timeline, *The Wire*; https://thewire.in/covid-19-india-timeline.
4. WHO Director-General's opening remarks at the media briefing on COVID-19 - 11 March 2020: www.who.int/director-general/speeches/detail/who-director-general-s-opening-remarks-at-the-media-briefing-on-covid-19---11-march-2020.
5. D. Ray, M. Salvatore, R. Bhattacharyya, L. Wang, J. Du, S. Mohammed, S. Purkayastha, A. Halder, A. Rix, D. Barker, M. Kleinsasser, Y. Zhou, D. Bose, P. Song, M. Banerjee, V. Baladandayuthapani, P. Ghosh, B. Mukherjee, Predictions, role of interventions and effects of a historic national lockdown in India's response to the the COVID-19 pandemic: Data science call to arms. *Harv. Data Sci. Rev.* 10.1162/99608f92.60e08ed5 (2020).
6. T. N. Service, Centre extends nationwide lockdown till May 31, new guidelines issued, *Trib. News Serv.*; www.tribuneindia.com/news/nation/centre-extends-nationwide-lockdown-till-may-31-new-guidelines-issued-86042.
7. J. Gettleman, K. Schultz, Modi Orders 3-Week Total Lockdown for All 1.3 Billion Indians, *N. Y. Times*, 2020: www.nytimes.com/2020/03/24/world/asia/india-coronavirus-lockdown.html.
8. U. Bhaskar, India to remain closed till 3 May, economy to open up gradually in lockdown 2.0, *mint*, 2020: www.livemint.com/news/india/pm-modi-announces-extension-of-lockdown-till-3-may-11586839412073.html.
9. G. R. Babu, D. Ray, R. Bhaduri, A. Halder, R. Kundu, G. I. Menon, B. Mukherjee, COVID-19 pandemic in India: Through the lens of modeling. *Glob. Health Sci. Pract.* **9**, 220–228 (2021).
10. "Unlock1": Malls, Restaurants, Places Of Worship To Reopen June 8, *NDTV.com*; www.ndtv.com/india-news/lockdown-extended-till-june-30-malls-restaurants-can-reopen-from-june-8-except-in-containment-zones-2237910.
11. M. Salvatore, D. Basu, D. Ray, M. Kleinsasser, S. Purkayastha, R. Bhattacharyya, B. Mukherjee, Comprehensive public health evaluation of lockdown as a non-pharmaceutical intervention on COVID-19 spread in India: National trends masking state-level variations. *BMJ Open* **10**, e041778 (2020).
12. J. Jesline, J. Romate, E. Rajkumar, A. J. George, The plight of migrants during COVID-19 and the impact of circular migration in India: A systematic review. *Humanit. Soc. Sci. Commun.* **8**, 1–12 (2021).
13. A. K. Enugu, J. Thakkar, S. Ghosh, R. Pollard, A. M. McFall, C. K. Vasudevan, E. Thamburaj, A. Singh, S. H. Mehta, S. S. Solomon, COVID-19 impact on index testing services in 5 high hiv prevalence Indian districts, *CROI Conf.*; www.croiconference.org/abstract/covid-19-impact-on-index-testing-services-in-5-high-hiv-prevalence-indian-districts/.
14. R. Shrinivasan, S. Rane, M. Pai, India's syndemic of tuberculosis and COVID-19. *BMJ Glob. Health* **5**, e003979 (2020).
15. UNICEF, COVID-19 pandemic leads to major backsliding on childhood vaccinations, new WHO, UNICEF data shows; www.unicef.org/press-releases/covid-19-pandemic-leads-major-backsliding-childhood-vaccinations-new-who-unicef-data.
16. COVID-19 India, COVID-19 Tracker Updates for India for State-wise and District-wise data, 2020; www.covid19india.org/.
17. M. V. Murhekar, T. Bhatnagar, J. W. V. Thangaraj, V. Saravanakumar, M. S. Kumar, S. Selvaraju, K. Rade, C. P. G. Kumar, R. Sabarinathan, A. Turuk, S. Asthana, R. Balachandar, S. D. Bangar, A. K. Bansal, V. Chopra, D. Das, A. K. Deb, K. R. Devi, V. Dhikav, G. R. Dwivedi, S. M. S. Khan, M. S. Kumar, A. Laxmaiah, M. Madhukar, A. Mahapatra, C. Rangaraju, J. Turuk, R. Yadav, R. Andhalkar, K. Arunraj, D. K. Bharadwaj, P. Bharti, D. Bhattacharya, J. Bhat, A. S. Chahal, D. Chakraborty, A. Chaudhury, H. Deval, S. Dhatrak, R. Dayal, D. Elantamilan, P. Giridharan, I. Haq, R. K. Hudda, B. Jagjeevan, A. Kalliath, S. Kanungo, N. N. Krishnan, J. S. Kshatri, A. Kumar, N. Kumar, V. G. V. Kumar, G. G. J. N. Lakshmi, G. Mehta, N. K. Mishra, A. Mitra, K. Nagbhushanam, A. Nimmathota, A. R. Nirmala, A. K. Pandey, G. V. Prasad, M. A. Qurieshi, S. D. Reddy, A. Robinson, S. Sahay, R. Saxena, K. Sekar, V. K. Shukla, H. B. Singh, P. K. Singh, P. Singh, R. Singh, N. Srinivasan, D. S. Varma, A. Viramgami, V. C. Wilson, S. Yadav, S. Yadav, K. Zaman, A. Chakrabarti, A. Das, R. S. Dhaliwal, S. Dutta, R. Kant, A. M. Khan, K. Narain, S. Narasimhaiah, C. Padmapriyadarshini, K. Pandey, S. Pati, S. Patil, H. Rajkumar, T. Ramarao, Y. K. Sharma, S. Singh, S. Panda, D. C. S. Reddy, B. Bhargava; ICMR Serosurveillance Group, SARS-CoV-2 seroprevalence among the general population and healthcare workers in India, December 2020-January 2021. *Int. J. Infect. Dis. IJID Off. Publ. Int. Soc. Infect. Dis.* **108**, 145–155 (2021).
18. Coronavirus | World's largest vaccination programme begins in India on January 16, *The Hindu* (2021); www.thehindu.com/news/national/coronavirus-worlds-largest-vaccination-programme-begins-in-india-on-january-16/article33582069.ece.
19. A. Deshpande, Maharashtra imposes 'mini lockdown' amid rising coronavirus cases, *The Hindu* (2021); www.thehindu.com/news/national/coronavirus-curfew-in-maharashtra-for-15-days-from-april-14-says-uddhav-thackeray/article34313068.ece.
20. K. Kuppalli, P. Gala, K. Cherabuddi, S. P. Kalantri, M. Mohanan, B. Mukherjee, L. Pinto, M. Prakash, C. S. Pramesh, S. Rathi, N. P. Pai, G. Yamey, M. Pai, India's COVID-19 crisis: A call for international action. *The Lancet* **397**, 2132–2135 (2021).
21. The RECOVERY, Dexamethasone in hospitalized patients with Covid-19. *N. Engl. J. Med.* **384**, 693–704 (2021).
22. V. Pandey, S. Nazmi, Covid-19 in India: Why second coronavirus wave is devastating, *BBC News*, 2021; www.bbc.com/news/world-asia-india-56811315.
23. L. Zimmermann, S. Bhattacharya, S. Purkayastha, R. Kundu, R. Bhaduri, P. Ghosh, B. Mukherjee, SARS-CoV-2 infection fatality rates in India: Systematic review, meta-analysis and model-based estimation. *Stud. Microecon.* 23210222211054324 (2021).
24. H. Wang, K. R. Paulson, S. A. Pease, S. Watson, H. Comfort, P. Zheng, A. Y. Aravkin, C. Bisignano, R. M. Barber, T. Alam, J. E. Fuller, E. A. May, D. P. Jones, M. E. Frisch, C. Abbafati, C. Adolph, A. Allorant, J. O. Amlag, B. Bang-Jensen, G. J. Bertolacci, S. S. Bloom, A. Carter, E. Castro, S. Chakrabarti, J. Chattopadhyay, R. M. Cogen, J. K. Collins, K. Cooperrider, X. Dai, W. J. Dangel, F. Daoud, C. Dapper, A. Deen, B. B. Duncan, M. Erickson, S. B. Ewald, T. Fedosseeva, A. J. Ferrari, J. J. Frostad, N. Fullman, J. Gallagher, A. Gamkrelidze, G. Guo, J. He, M. Helak, N. J. Henry, E. N. Hulland, B. M. Huntley, M. Kereselidze, A. Lazzar-Atwood, K. E. LeGrand, A. Lindstrom, E. Linebarger, P. A. Lotufo, R. Lozano, B. Magistro, D. C. Malta, J. Månsson, A. M. M. Herrera, F. Marinho, A. H. Mirkuzie, A. T. Misganaw, L. Monasta, P. Naik, S. Nomura, E. G. O'Brien, J. K. O'Halloran, L. T. Olana, S. M. Ostroff, L. Penberthy, R. C. Reiner Jr., G. Reinke, A. L. P. Ribeiro, D. F. Santomauro, M. I. Schmidt, D. H. Shaw, B. S. Sheena, A. Sholokhov, N. Skhvitaridze, R. J. D. Sorensen, E. E. Spurlock, R. Syailendrawati, R. Topor-Madry, C. E. Troeger, R. Walcott, A. Walker, C. S. Wiysonge, N. A. Worku, B. Zigler, D. M. Pigott, M. Naghavi, A. H. Mokdad, S. S. Lim, S. I. Hay, E. Gakidou, C. J. L. Murray, Estimating excess mortality due to the COVID-19 pandemic: A systematic analysis of COVID-19-related mortality, 2020–21. *Lancet* S0140673621027963 (2022).
25. P. Jha, Y. Deshmukh, C. Tumbe, W. Suraweera, A. Bhowmick, S. Sharma, P. Novosad, S. H. Fu, L. Newcombe, H. Gelband, P. Brown, COVID mortality in India: National survey data and health facility deaths. *Science* **375**, 667–671 (2022).
26. M. S. Dhar, R. Marwal, V. Radhakrishnan, K. Ponnusamy, B. Jolly, R. C. Bhoyar, S. Fatihi, M. Datta, P. Singh, U. Sharma, R. Ujjainia, S. Naushin, N. Bhateja, M. K. Divakar, V. Sardana, M. K. Singh, M. Imran, V. Senthivel, R. Maurya, N. Jha, P. Mehta, M. Rophina, V. Arvinden, U. Chaudhary, L. Thukral, R. Pandey, D. Dash, M. Faruq, H. Lall, H. Gogia, P. Madan, S. Kulkarni, H. Chauhan, S. Sengupta, S. Kabra; The Indian SARS-CoV-2 Genomics Consortium (INSACOG), S. K. Singh, A. Agrawal, P. Rakshit, "Genomic characterization and epidemiology of an emerging SARS-CoV-2 variant in Delhi, India" (medRxiv, 2021).
27. R. Sarkar, R. Saha, P. Mallick, R. Sharma, A. Kaur, S. Dutta, M. Chawla-Sarkar, "Emergence of a new SARS-CoV-2 variant from GR clade with a novel S glycoprotein mutation V1230L in West Bengal, India" (medRxiv, 2021).
28. World Health Organization, Tracking SARS-CoV-2 variants; www.who.int/en/activities/tracking-SARS-CoV-2-variants/.
29. Genome sequencing by INSACOG shows variants of concern and a Novel variant in India; pib.gov.in/Pressreleaseshare.aspx?PRID=1707177.
30. S. Cherian, V. Potdar, S. Jadhav, P. Yadav, N. Gupta, M. Das, P. Rakshit, S. Singh, P. Abraham, S. Panda, N. Team, SARS-CoV-2 Spike mutations, L452R, T478K, E484Q and P681R, in the second wave of COVID-19 in Maharashtra, India. *Microorganisms.* **9**, 1542 (2021).

31. Maharashtra: Double mutant found in 61% samples tested, *Indian Express*, 2021; https://indianexpress.com/article/cities/mumbai/maharashtra-double-mutant-found-in-61-samples-tested-7272524/.
32. World Health Organization, Weekly epidemiological update on COVID-19 - 11 May 2021 (2021); www.who.int/publications/m/item/weekly-epidemiological-update-on-covid-19---11-may-2021.
33. C. Pattabiraman, P. Prasad, A. K. George, D. Sreenivas, R. Rasheed, N. V. K. Reddy, A. Desai, R. Vasanthapuram, Importation, circulation, and emergence of variants of SARS-CoV-2 in the South Indian state of Karnataka. *Wellcome Open Res.* **6**, 110 (2021).
34. J. Koshy, Coronavirus | New virus lineage found in West Bengal, *The Hindu* (2021); www.thehindu.com/sci-tech/health/new-coronavirus-variant-found-in-west-bengal/article34373083.ece.
35. 81 per cent of Punjab COVID-19 samples sent for genome sequencing show new UK variant, *New Indian Express*, 2021; www.newindianexpress.com/nation/2021/mar/23/81-per-cent-of-punjab-covid-19-samples-sent-for-genome-sequencing-show-new-uk-variant-2280394.html.
36. F. Campbell, B. Archer, H. Laurenson-Schafer, Y. Jinnai, F. Konings, N. Batra, B. Pavlin, K. Vandemaele, M. D. Van Kerkhove, T. Jombart, O. Morgan, O. le Polain de Waroux, Increased transmissibility and global spread of SARS-CoV-2 variants of concern as at June 2021. *Eurosurveill.* **26**, 2100509 (2021).
37. L. Wang, Y. Zhou, J. He, B. Zhu, F. Wang, L. Tang, M. Kleinsasser, D. Barker, M. C. Eisenberg, P. X. K. Song, An epidemiological forecast model and software assessing interventions on the COVID-19 epidemic in China. *J. Data Sci.* **18**, 409–432 (2021).
38. J. Wangping, H. Ke, S. Yang, C. Wenzhe, W. Shengshu, Y. Shanshan, W. Jianwei, K. Fuyin, T. Penggang, L. Jing, L. Miao, H. Yao, Extended SIR prediction of the epidemics trend of covid-19 in italy and compared with Hunan, China. *Front. Med.* **7**, 169 (2020).
39. A. Cori, S. Cauchemez, N. M. Ferguson, C. Fraser, E. Dahlqwist, P. A. Demarsh, T. Jombart, Z. N. Kamvar, J. Lessler, S. Li, J. A. Polonsky, J. Stockwin, R. Thompson, R. van Gaalen, *EpiEstim: Estimate Time Varying Reproduction Numbers from Epidemic Curves* (2021); https://CRAN.R-project.org/package=EpiEstim.
40. A. Cori, N. M. Ferguson, C. Fraser, S. Cauchemez, A new framework and software to estimate time-varying reproduction numbers during epidemics. *Am. J. Epidemiol.* **178**, 1505–1512 (2013).
41. Y. Liu, J. Rocklöv, The reproductive number of the Delta variant of SARS-CoV-2 is far higher compared to the ancestral SARS-CoV-2 virus. *J. Travel Med.* **28**, taab124 (2021).
42. R. Bhaduri, R. Kundu, S. Purkayastha, M. Kleinsasser, L. J. Beesley, B. Mukherjee, J. Datta, Extending the susceptible-exposed-infected-removed (SEIR) model to handle the false negative rate and symptom-based administration of COVID-19 diagnostic tests: SEIR-fansy. *Stat. Med.* **41**, 2317–2337 (2022).
43. A. Ghosh, 4th sero survey finds 2 of 3 Indians with Covid antibodies, but still avoid crowds, ICMR warns, *ThePrint* (2021); https://theprint.in/health/4th-sero-survey-finds-2-of-3-indians-with-covid-antibodies-but-still-avoid-crowds-icmr-warns/699600/.
44. Y. Deshmukh, W. Suraweera, C. Tumbe, A. Bhowmick, S. Sharma, P. Novosad, S. H. Fu, L. Newcombe, H. Gelband, P. Brown, P. Jha, "Excess mortality in India from June 2020 to June 2021 during the COVID pandemic: Death registration, health facility deaths, and survey data" (medRxiv, 2021).
45. G. Kapoor, A. Sriram, J. Joshi, A. Nandi, R. Laxminarayan, COVID-19 in India: State-Wise Estimates of Current Hospital Beds, ICU Beds, and Ventilators, *CDDEP*, 2020; https://cddep.org/publications/covid-19-in-india-state-wise-estimates-of-current-hospital-beds-icu-beds-and-ventilators/.
46. K. Tseng, I. Frost, G. Kapoor, A. Sriram, A. Nandi, R. Laxminarayan, COVID-19 in India: State-level estimates of hospitalization needs, *CDDEP*; https://cddep.org/publications/covid-19-in-india-state-level-estimates-of-hospitalization-needs/.
47. A. Sibal, K. H. Prasad, S. Reddy, P. M. Doraiswamy, Apollo Hospitals and Project Kavach: Insights from How India's Largest Private Health System Is Handling Covid-19, *NEJM Catalyst*, 2021; https://catalyst.nejm.org/doi/full/10.1056/CAT.20.0677.
48. World Health Organization, Considerations for implementing and adjusting public health and social measures in the context of COVID-19 (2021); www.who.int/publications-detail-redirect/considerations-in-adjusting-public-health-and-social-measures-in-the-context-of-covid-19-interim-guidance.
49. Ontario Ministry of Health, "COVID-19 Response Framework: Keeping Ontario Safe and Open—Lockdown Measures" (2020), p. 35.
50. Scottish Government, Coronavirus (COVID-19): Scotland's route map through and out of the crisis (2020); www.gov.scot/publications/coronavirus-covid-19-framework-decision-making-scotlands-route-map-through-out-crisis/.
51. Singapore Ministry of Health, "MOH Pandemic Readiness and Response Plan for Influenza and Other Acute Respiratory Diseases" (2014); www.moh.gov.sg/docs/librariesprovider5/diseases-updates/interim-pandemic-plan-public-ver-_april-2014.pdf.
52. Johns Hopkins Coronavirus Resource Center, India - COVID-19 Overview; https://coronavirus.jhu.edu/region/india.
53. K. A. Twohig, T. Nyberg, A. Zaidi, S. Thelwall, M. A. Sinnathamby, S. Aliabadi, S. R. Seaman, R. J. Harris, R. Hope, J. Lopez-Bernal, E. Gallagher, A. Charlett, D. De Angelis, A. M. Presanis, G. Dabrera; COVID-19 Genomics UK (COG-UK) consortium, Hospital admission and emergency care attendance risk for SARS-CoV-2 delta (B.1.617.2) compared with alpha (B.1.1.7) variants of concern: A cohort study. *Lancet Infect. Dis.* **22**, 35–42 (2022).
54. R. Viana, S. Moyo, D. G. Amoako, H. Tegally, C. Scheepers, C. L. Althaus, U. J. Anyaneji, P. A. Bester, M. F. Boni, M. Chand, W. T. Choga, R. Colquhoun, M. Davids, K. Deforche, D. Doolabh, L. du Plessis, S. Engelbrecht, J. Everatt, J. Giandhari, M. Giovanetti, D. Hardie, V. Hill, N.-Y. Hsiao, A. Iranzadeh, A. Ismail, C. Joseph, R. Joseph, L. Koopile, S. L. K. Pond, M. U. G. Kraemer, L. Kuate-Lere, O. Laguda-Akingba, O. Lesetedi-Mafoko, R. J. Lessells, S. Lockman, A. G. Lucaci, A. Maharaj, B. Mahlangu, T. Maponga, K. Mahlakwane, Z. Makatini, G. Marais, D. Maruapula, K. Masupu, M. Matshaba, S. Mayaphi, N. Mbhele, M. B. Mbulawa, A. Mendes, K. Mlisana, A. Mnguni, T. Mohale, M. Moir, K. Moruisi, M. Mosepele, G. Motsatsi, M. S. Motswaledi, T. Mphoyakgosi, N. Msomi, P. N. Mwangi, Y. Naidoo, N. Ntuli, M. Nyaga, L. Olubayo, S. Pillay, B. Radibe, Y. Ramphal, U. Ramphal, J. E. San, L. Scott, R. Shapiro, L. Singh, P. Smith-Lawrence, W. Stevens, A. Strydom, K. Subramoney, N. Tebeila, D. Tshiabuila, J. Tsui, S. van Wyk, S. Weaver, C. K. Wibmer, E. Wilkinson, N. Wolter, A. E. Zarebski, B. Zuze, D. Goedhals, W. Preiser, F. Treurnicht, M. Venter, C. Williamson, O. G. Pybus, J. Bhiman, A. Glass, D. P. Martin, A. Rambaut, S. Gaseitsiwe, A. von Gottberg, T. de Oliveira, Rapid epidemic expansion of the SARS-CoV-2 omicron variant in southern Africa. *Nature* **603**, 679–686 (2022).
55. W. Ma, J. Yang, H. Fu, C. Su, C. Yu, Q. Wang, A. T. R. de Vasconcelos, G. A. Bazykin, Y. Bao, M. Li, Genomic perspectives on the emerging SARS-CoV-2 omicron variant. *Genom. Proteom. Bioinform.* , S1672-0229(22)00002-X (2022).
56. H. de Puig, R. A. Lee, D. Najjar, X. Tan, L. R. Soekensen, N. M. Angenent-Mari, N. M. Donghia, N. E. Weckman, A. Ory, C. F. Ng, P. Q. Nguyen, A. S. Mao, T. C. Ferrante, G. Lansberry, H. Sallum, J. Niemi, J. J. Collins, Minimally instrumented SHERLOCK (miSHERLOCK) for CRISPR-based point-of-care diagnosis of SARS-CoV-2 and emerging variants. *Sci. Adv.* **7**, eabh2944 (2021).
57. M. Hemalatha, U. Kiran, S. K. Kuncha, H. Kopperi, C. G. Gokulan, S. V. Mohan, R. K. Mishra, Surveillance of SARS-CoV-2 spread using wastewater-based epidemiology: Comprehensive study. *Sci. Total Environ.* **768**, 144704 (2021).
58. S. Priyadarshini, India's sewage surveillance for SARS-CoV-2 going down the drain. *Nature* (2021).
59. Indian Biological Data Centre, INSACOG Covid-19 Data. Indian Nucleotide Data Arch. – Control, *Access INDA-CA*; http://inda.rcb.ac.in:8080/insacog/indexpage.
60. S. Purkayastha, R. Kundu, R. Bhaduri, D. Barker, M. Kleinsasser, D. Ray, B. Mukherjee, Estimating the wave 1 and wave 2 infection fatality rates from SARS-CoV-2 in India. *BMC. Res. Notes* **14**, 262 (2021).
61. R. Subbaraman, L. Ganapathi, B. Mukherjee, D. E. Bloom, S. S. Solomon, Humane shelter at home: A call to reimagine a core pandemic intervention. *BMJ Glob. Health* **6**, e006614 (2021).
62. G. Meyerowitz-Katz, S. Bhatt, O. Ratmann, J. M. Brauner, S. Flaxman, S. Mishra, M. Sharma, S. Mindermann, V. Bradley, M. Vollmer, L. Merone, G. Yamey, Is the cure really worse than the disease? The health impacts of lockdowns during COVID-19. *BMJ Glob. Health* **6**, e006653 (2021).
63. India Today Web Desk New Delhi, Full list of Red, Yellow, Green Zone districts for Lockdown 3.0, *India Today*, 2020; www.indiatoday.in/india/story/red-orange-green-zones-full-current-update-list-districts-states-india-coronavirus-1673358-2020-05-01.
64. Y. Bo, C. Guo, C. Lin, Y. Zeng, H. B. Li, Y. Zhang, M. S. Hossain, J. W. M. Chan, D. W. Yeung, K. O. Kwok, S. Y. S. Wong, A. K. H. Lau, X. Q. Lao, Effectiveness of non-pharmaceutical interventions on COVID-19 transmission in 190 countries from 23 January to 13 April 2020. *Int. J. Infect. Dis.* **102**, 247–253 (2021).
65. S. Pei, S. Kandula, J. Shaman, Differential effects of intervention timing on COVID-19 spread in the United States. *Sci. Adv.* **6**, eabd6370 (2020).
66. A. Mendez-Brito, C. El Bcheraoui, F. Pozo-Martin, Systematic review of empirical studies comparing the effectiveness of non-pharmaceutical interventions against COVID-19. *J. Infect.* **83**, 281–293 (2021).
67. Institute for Health Metrics and Evaluation, Causes of Death in India, 2009–2019, *GBD Comp.*; http://vizhub.healthdata.org/gbd-compare.
68. A. I. Middya, S. Roy, Geographically varying relationships of COVID-19 mortality with different factors in India. *Sci. Rep.* **11**, 7890 (2021).
69. N. Arinaminpathy, J. Das, T. H. McCormick, P. Mukhopadhyay, N. Sircar, Quantifying heterogeneity in SARS-CoV-2 transmission during the lockdown in India. *Epidemics* **36**, 100477 (2021).
70. H. Ritchie, E. Mathieu, L. Rodés-Guirao, C. Appel, C. Giattino, E. Ortiz-Ospina, J. Hasell, B. Macdonald, D. Beltekian, M. Roser, Coronavirus (COVID-19) Vaccinations (Our World in Data, 2020); https://ourworldindata.org/covid-vaccinations.
71. S. K C, Covid-19 vaccination drive: Over 10 million administered doses on Tuesday, 500 million given first dose so far, *Hindustan Times*, 2021; www.hindustantimes.com/india-news/covid19-vaccination-drive-over-10-million-administered-doses-on-tuesday-500-million-given-first-dose-so-far-101630417853792.html.

72. Microsoft, Microsoft Bing COVID-19 Tracker; www.bing.com/covid?ref=share.
73. S. S. A. Karim, Q. A. Karim, Omicron SARS-CoV-2 variant: A new chapter in the COVID-19 pandemic. *Lancet* **398**, 2126–2128 (2021).
74. Unique Identification Authority of India, "State/UT wise Aadhar Saturation" (2020); https://uidai.gov.in/images/state-wise-aadhaar-saturation.pdf.
75. M. Salvatore, S. Purkayastha, R. Kundu, *umich-cphds/covid_india_wave2:* (Zenodo, 2022); https://zenodo.org/record/6514992.
76. Y. Zhou, L. Wang, L. Zhang, L. Shi, K. Yang, J. He, B. Zhao, W. Overton, S. Purkayastha, P. Song, A spatiotemporal epidemiological prediction model to inform county-level COVID-19 risk in the United States. *Harv. Data Sci. Rev.* 10.1162/99608f92.79e1f45e (2020).
77. N. G. Davies, S. Abbott, R. C. Barnard, C. I. Jarvis, A. J. Kucharski, J. D. Munday, C. A. B. Pearson, T. W. Russell, D. C. Tully, A. D. Washburne, T. Wenseleers, A. Gimma, W. Waites, K. L. M. Wong, K. van Zandvoort, J. D. Silverman; CMMID COVID-19 Working Group, COVID-19 Genomics UK (COG-UK) Consortium, K. Diaz-Ordaz, R. Keogh, R. M. Eggo, S. Funk, M. Jit, K. E. Atkins, W. J. Edmunds, Estimated transmissibility and impact of SARS-CoV-2 lineage B.1.1.7 in England. *Science* **372**, eabg3055 (2021).
78. CSIR Institute of Genomics & Integrative Biology, COVID-19 Genomic Surveillance; https://clingen.igib.res.in/covid19genomes/.
79. C. H. Hansen, D. Michlmayr, S. M. Gubbels, K. Mølbak, S. Ethelberg, Assessment of protection against reinfection with SARS-CoV-2 among 4 million PCR-tested individuals in Denmark in 2020: A population-level observational study. *The Lancet* **397**, 1204–1212 (2021).
80. *R package SEIRfansy* (Department of Biostatistics at the University of Michigan, 2021); https://github.com/umich-biostatistics/SEIRfansy.
81. M. Robinson, N. I. Stilianakis, A model for the emergence of drug resistance in the presence of asymptomatic infections. *Math. Biosci.* **243**, 163–177 (2013).
82. T. Mkhatshwa, A. Mummert, Modeling super-spreading events for infectious diseases: Case study SARS. arXiv:1007.0908 [q-bio.PE] (2010); http://arxiv.org/abs/1007.0908.
83. N. Chen, M. Zhou, X. Dong, J. Qu, F. Gong, Y. Han, Y. Qiu, J. Wang, Y. Liu, Y. Wei, J. Xia, T. Yu, X. Zhang, L. Zhang, Epidemiological and clinical characteristics of 99 cases of 2019 novel coronavirus pneumonia in Wuhan, China: A descriptive study. *Lancet* **395**, 507–513 (2020).
84. N. R. Faria, T. A. Mellan, C. Whittaker, I. M. Claro, D. d. S. Jesus, S. Mishra, M. A. E. Crispim, F. C. S. Sales, I. Hawryluk, J. T. McCrone, R. J. G. Hulswit, L. A. M. Franco, M. S. Ramundo, J. G. de Jesus, P. S. Andrade, T. M. Coletti, G. M. Ferreira, C. A. M. Silva, E. R. Manuli, R. H. M. Pereira, P. S. Peixoto, M. U. G. Kraemer, N. Gaburo, C. d. C. Camilo, H. Hoeltgebaum, W. M. Souza, E. C. Rocha, L. M. de Souza, M. C. de Pinho, L. J. T. Araujo, F. S. V. Malta, A. B. de Lima, J. d. P. Silva, D. A. G. Zauli, A. C. d. S. Ferreira, R. P. Schnekenberg, D. J. Laydon, P. G. T. Walker, H. M. Schlüter, A. L. P. dos Santos, M. S. Vidal, V. S. D. Caro, R. M. F. Filho, H. M. dos Santos, R. S. Aguiar, J. L. Proença-Modena, B. Nelson, J. A. Hay, M. Monod, X. Miscouridou, H. Coupland, R. Sonabend, M. Vollmer, A. Gandy, C. A. Prete, V. H. Nascimento, M. A. Suchard, T. A. Bowden, S. L. K. Pond, C.-H. Wu, O. Ratmann, N. M. Ferguson, C. Dye, N. J. Loman, P. Lemey, A. Rambaut, N. A. Fraiji, M. d. P. S. S. Carvalho, O. G. Pybus, S. Flaxman, S. Bhatt, E. C. Sabino, Genomics and epidemiology of the P.1 SARS-CoV-2 lineage in Manaus, Brazil. *Science* **372**, 815–821 (2021).
85. S. Das, Prediction of COVID-19 disease progression in India: Under the effect of national lockdown. arXiv:2004.03147 [q-bio.PE] (2020); http://arxiv.org/abs/2004.03147.
86. S. Deb, M. Majumdar, A time series method to analyze incidence pattern and estimate reproduction number of COVID-19. arXiv:2003.10655 [stat.AP] (2020); http://arxiv.org/abs/2003.10655.
87. R. Ranjan, "Predictions for COVID-19 outbreak in India using epidemiological models" (preprint, Epidemiology, 2020).
88. T. Sardar, S. S. Nadim, S. Rana, J. Chattopadhyay, Assessment of lockdown effect in some states and overall India: A predictive mathematical study on COVID-19 outbreak. *Chaos Solitons Fractals* **139**, 110078 (2020).
89. R. Singh, R. Adhikari, Age-structured impact of social distancing on the COVID-19 epidemic in India. arXiv:2003.12055 [q-bio.PE] (2020); http://arxiv.org/abs/2003.12055.
90. Q. Li, X. Guan, P. Wu, X. Wang, L. Zhou, Y. Tong, R. Ren, K. S. M. Leung, E. H. Y. Lau, J. Y. Wong, X. Xing, N. Xiang, Y. Wu, C. Li, Q. Chen, D. Li, T. Liu, J. Zhao, M. Liu, W. Tu, C. Chen, L. Jin, R. Yang, Q. Wang, S. Zhou, R. Wang, H. Liu, Y. Luo, Y. Liu, G. Shao, H. Li, Z. Tao, Y. Yang, Z. Deng, B. Liu, Z. Ma, Y. Zhang, G. Shi, T. T. Y. Lam, J. T. Wu, G. F. Gao, B. J. Cowling, B. Yang, G. M. Leung, Z. Feng, Early transmission dynamics in Wuhan, China, of novel coronavirus–infected pneumonia. *N. Engl. J. Med.* **382**, 1199–1207 (2020).
91. S. Ryu, B. C. Chun, An interim review of the epidemiological characteristics of 2019 novel coronavirus. *Epidemiol. Health.* **42**, e2020006 (2020).
92. M. Murhekar, T. Bhatnagar, S. Selvaraju, K. Rade, V. Saravanakumar, J. V. Thangaraj, M. Kumar, N. Shah, R. Sabarinathan, A. Turuk, P. Anand, S. Asthana, R. Balachandar, S. Bangar, A. Bansal, J. Bhat, D. Chakraborty, C. Rangaraju, V. Chopra, D. Das, A. Deb, K. Devi, G. Dwivedi, S. M. S. Khan, I. Haq, M. S. Kumar, A. Laxmaiah, Madhuka, A. Mahapatra, A. Mitra, A. Nirmala, A. Pagdhune, M. Qurieshi, T. Ramarao, S. Sahay, Y. Sharma, M. Shrinivasa, V. Shukla, P. Singh, A. Viramgami, V. Wilson, R. Yadav, C. G. Kumar, H. Luke, U. Ranganathan, S. Babu, K. Sekar, P. Yadav, G. Sapkal, A. Das, P. Das, S. Dutta, R. Hemalatha, A. Kumar, K. Narain, S. Narasimhaiah, S. Panda, S. Pati, S. Patil, K. Sarkar, S. Singh, R. Kant, S. Tripathy, G. Toteja, G. Babu, S. Kant, J. Muliyil, R. Pandey, S. Sarkar, S. Singh, S. Zodpey, R. Gangakhedkar, D. S. Reddy, B. Bhargava, Prevalence of SARS-CoV-2 infection in India: Findings from the national serosurvey, May-June 2020. *Indian J. Med. Res.* **152**, 48 (2020).
93. M. V. Murhekar, T. Bhatnagar, S. Selvaraju, V. Saravanakumar, J. W. V. Thangaraj, N. Shah, M. S. Kumar, K. Rade, R. Sabarinathan, S. Asthana, R. Balachandar, S. D. Bangar, A. K. Bansal, J. Bhat, V. Chopra, D. Das, A. K. Deb, K. R. Devi, G. R. Dwivedi, S. M. S. Khan, C. P. G. Kumar, M. S. Kumar, A. Laxmaiah, M. Madhukar, A. Mahapatra, S. S. Mohanty, C. Rangaraju, A. Turuk, D. K. Baradwaj, A. S. Chahal, F. Debnath, I. Haq, A. Kalliath, S. Kanungo, J. S. Kshatri, G. G. J. N. Lakshmi, A. Mitra, A. R. Nirmala, G. V. Prasad, M. A. Qurieshi, S. Sahay, R. K. Sangwan, K. Sekar, V. K. Shukla, P. K. Singh, P. Singh, R. Singh, D. S. Varma, A. Viramgami, S. Panda, D. C. S. Reddy, B. Bhargava, R. Andhalkar, A. Chaudhury, H. Deval, S. Dhatrak, R. R. Gupta, E. Ilayaperumal, B. Jagjeevan, R. C. Jha, K. Kiran, N. N. Krishnan, A. Kumar, V. V. Kumar, K. Nagbhushanam, A. Nimmathota, A. K. Pandey, H. S. Pawar, K. S. Rathore, A. Robinson, H. B. Singh, V. C. Wilson, A. Yadav, R. Yadav, T. Karunakaran, J. Pradhan, T. Sivakumar, A. Jose, K. Kalaiyarasi, S. Dasgupta, R. Anusha, T. Anand, G. R. Babu, H. Chauhan, T. Dikid, R. R. Gangakhedkar, S. Kant, S. Kulkarni, J. P. Muliyil, R. M. Pandey, S. Sarkar, A. Shrivastava, S. K. Singh, S. Zodpey, A. Das, P. Das, S. Dutta, R. Kant, K. Narain, S. Narasimhaiah, S. Pati, S. Patil, H. Rajkumar, T. Ramarao, K. Sarkar, S. Singh, G. S. Toteja, K. Zaman, SARS-CoV-2 antibody seroprevalence in India, August–September, 2020: Findings from the second nationwide household serosurvey. *Lancet Glob. Health* **9**, e257–e266 (2021).
94. S. Goswami, Fourth serosurvey finds 67.6% have antibodies, 40 crore Indians still vulnerable, Hindustan Times (2021); www.hindustantimes.com/india-news/40-cr-indians-don-t-have-covid-anti-bodies-vulnerable-reveals-4th-serosurvey-101626779228818.html.
95. S. A. Lauer, K. H. Grantz, Q. Bi, F. K. Jones, Q. Zheng, H. R. Meredith, A. S. Azman, N. G. Reich, J. Lessler, The incubation period of coronavirus disease 2019 (COVID-19) from publicly reported confirmed cases: Estimation and application. *Ann. Intern. Med.* **172**, 577–582 (2020).
96. R. Verity, L. C. Okell, I. Dorigatti, P. Winskill, C. Whittaker, N. Imai, G. Cuomo-Dannenburg, H. Thompson, P. G. T. Walker, H. Fu, A. Dighe, J. T. Griffin, M. Baguelin, S. Bhatia, A. Boonyasiri, A. Cori, Z. Cucunubá, R. FitzJohn, K. Gaythorpe, W. Green, A. Hamlet, W. Hinsley, D. Laydon, G. Nedjati-Gilani, S. Riley, S. van Elsland, E. Volz, H. Wang, Y. Wang, X. Xi, C. A. Donnelly, A. C. Ghani, N. M. Ferguson, Estimates of the severity of coronavirus disease 2019: A model-based analysis. *Lancet Infect. Dis.* **20**, 669–677 (2020).
97. India Statistics and Maps, *Atlas Big* (1970); www.atlasbig.com/en-us/india.
98. S. Bhaumik, D. Beri, M. Seerat, N. Gudi, Roopani, R. Dhurjati, N. Dumka, J. Tyagi, "Preparedness for crises response to the second wave of COVID-19 in India: Policy brief" (The George Institute for Global Health, India, National Health Systems Resource Centre, 2021).
99. T. C. Bulfone, M. Malekinejad, G. W. Rutherford, N. Razani, Outdoor transmission of SARS-CoV-2 and other respiratory viruses: A systematic review. *J Infect Dis* **223**, 550–561 (2021).
100. N. Razani, M. Malekinejad, G. W. Rutherford, Clarification regarding outdoor transmission of SARS-CoV-2 and other respiratory viruses, a systematic review. *J. Infect Dis* **224**, 925–926 (2021).
101. K. A. Fisher, M. W. Tenforde, L. R. Feldstein, C. J. Lindsell, N. I. Shapiro, D. C. Files, K. W. Gibbs, H. L. Erickson, M. E. Prekker, J. S. Steingrub, M. C. Exline, D. J. Henning, J. G. Wilson, S. M. Brown, I. D. Peltan, T. W. Rice, D. N. Hager, A. A. Ginde, H. K. Talbot, J. D. Casey, C. G. Grijalva, B. Flannery, M. M. Patel, W. H. Self; IVY Network Investigators, CDC COVID-19 Response Team, IVY Network Investigators, K. W. Hart, R. McClellan, H. Tan, A. Baughman; CDC COVID-19 Response Team, N. A. Hennesy, B. Grear, M. Wu, K. Mlynarczyk, L. Marzano, Z. Plata, A. Caplan, S. M. Olson, C. E. Ogokeh, E. R. Smith, S. S. Kim, E. P. Griggs, B. Richards, S. Robinson, K. Kim, A. M. Kassem, C. N. Sciarratta, P. L. Marcet, Community and close contact exposures associated with COVID-19 among symptomatic adults ≥18 years in 11 outpatient health care facilities — United States, July 2020. *MMWR Morb. Mortal. Wkly Rep.* **69**, 1258–1264 (2020).
102. What is a Lockdown? *Bus. Stand. India*; www.business-standard.com/about/what-is-lockdown#collapse.
103. Ministry of Home Affairs, "Annexure to Ministry of Home Affairs Order No .40-3/2020-D dated ()24.03.2020" (40-3/2020-D, 2020); www.mha.gov.in/sites/default/files/Guidelines.pdf.
104. L. Mathew, A. Ghosh, PM Modi declares lockdown: 'Crossing your home's lakshmanrekha will invite COVID-19 inside,' *Indian Express*, 2020; https://indianexpress.com/article/india/pm-modi-declares-lockdown-crossing-your-homes-lakshmanrekha-will-invite-covid-19-inside-6330017/.
105. Covid-19 India timeline: Looking back at pandemic-induced lockdown and how the country is coping with the crisis, *Indian Express*, 2021; https://indianexpress.com/article/india/covid-19-india-timeline-looking-back-at-pandemic-induced-lockdown-7241583/.
106. Maharashtra brings back Covid-19 e-pass for inter-state, inter-district travel: Here's how to apply, *Hindustan Times*, 2021; www.hindustantimes.com/cities/mumbai-news/maharashtra-brings-back-covid-19-e-pass-for-inter-state-inter-district-travel-here-s-how-to-apply-101619161303652.html.

107. N. Banholzer, E. van Weenen, A. Lison, A. Cenedese, A. Seeliger, B. Kratzwald, D. Tschernutter, J. P. Salles, P. Bottrighi, S. Lehtinen, S. Feuerriegel, W. Vach, Estimating the effects of non-pharmaceutical interventions on the number of new infections with COVID-19 during the first epidemic wave. *PLOS ONE* **16**, e0252827 (2021).
108. J. M. Brauner, S. Mindermann, M. Sharma, D. Johnston, J. Salvatier, T. Gavenčiak, A. B. Stephenson, G. Leech, G. Altman, V. Mikulik, A. J. Norman, J. T. Monrad, T. Besiroglu, H. Ge, M. A. Hartwick, Y. W. Teh, L. Chindelevitch, Y. Gal, J. Kulveit, Inferring the effectiveness of government interventions against COVID-19. *Science* **371**, eabd9338 (2021).
109. S. Talic, S. Shah, H. Wild, D. Gasevic, A. Maharaj, Z. Ademi, X. Li, W. Xu, I. Mesa-Eguiagaray, J. Rostron, E. Theodoratou, X. Zhang, A. Motee, D. Liew, D. Ilic, Effectiveness of public health measures in reducing the incidence of covid-19, SARS-CoV-2 transmission, and covid-19 mortality: Systematic review and meta-analysis. *BMJ* **375**, e068302 (2021).
110. V. Barros, I. Manes, V. Akinwande, C. Cintas, O. Bar-Shira, M. Ozery-Flato, Y. Shimoni, M. Rosen-Zvi, A causal inference approach for estimating effects of non-pharmaceutical interventions during Covid-19 pandemic (2022), p. 2022.02.28.22271671.
111. S. Flaxman, S. Mishra, A. Gandy, H. J. T. Unwin, T. A. Mellan, H. Coupland, C. Whittaker, H. Zhu, T. Berah, J. W. Eaton, M. Monod; Imperial College COVID-19 Response Team, P. N. Perez-Guzman, N. Schmit, L. Cilloni, K. E. C. Ainslie, M. Baguelin, A. Boonyasiri, O. Boyd, L. Cattarino, L. V. Cooper, Z. Cucunubá, G. Cuomo-Dannenburg, A. Dighe, B. Djaafara, I. Dorigatti, S. L. van Elsland, R. G. FitzJohn, K. A. M. Gaythorpe, L. Geidelberg, N. C. Grassly, W. D. Green, T. Hallett, A. Hamlet, W. Hinsley, B. Jeffrey, E. Knock, D. J. Laydon, G. Nedjati-Gilani, P. Nouvellet, K. V. Parag, I. Siveroni, H. A. Thompson, R. Verity, E. Volz, C. E. Walters, H. Wang, Y. Wang, O. J. Watson, P. Winskill, X. Xi, P. G. T. Walker, A. C. Ghani, C. A. Donnelly, S. Riley, M. A. C. Vollmer, N. M. Ferguson, L. C. Okell, S. Bhatt, Estimating the effects of non-pharmaceutical interventions on COVID-19 in Europe. *Nature* **584**, 257–261 (2020).
112. J. Abaluck, L. H. Kwong, A. Styczynski, A. Haque, M. A. Kabir, E. Bates-Jefferys, E. Crawford, J. Benjamin-Chung, S. Raihan, S. Rahman, S. Benhachmi, N. Z. Bintee, P. J. Winch, M. Hossain, H. M. Reza, A. A. Jaber, S. G. Momen, A. Rahman, F. L. Banti, T. S. Huq, S. P. Luby, A. M. Mobarak, Impact of community masking on COVID-19: A cluster-randomized trial in Bangladesh. *Science* **375**, eabi9069 (2022).
113. Z. Zou, C. K. Fairley, M. Shen, N. Scott, X. Xu, Z. Li, R. Li, G. Zhuang, L. Zhang, Critical timing and extent of public health interventions to control outbreaks dominated by SARS-CoV-2 variants in Australia: A mathematical modelling study. *Int. J. Infect. Dis.* **115**, 154–165 (2022).
114. V. P. Bajiya, S. Bugalia, J. P. Tripathi, Mathematical modeling of COVID-19: Impact of non-pharmaceutical interventions in India. *Chaos Interdiscip. J. Nonlinear Sci.* **30**, 113143 (2020).

Acknowledgments: We are grateful to S. Mishra, S. Flaxman, S. Bhatt, T. Mellan, and C. Whittaker from the Imperial College, London for input on a previous version of the manuscript. We thank L. Wang for advisement regarding the use of eSIR R package and P. Song for guidance based on expertise of the eSIR model and the COVID-19 outbreak in China. We also thank INSACOG for efforts in collecting and sharing COVID-19 sequencing data from India. This work is licensed under a Creative Commons Attribution 4.0 International (CC BY 4.0) license, which permits unrestricted use, distribution, and reproduction in any medium, provided that the original work is properly cited. To view a copy of this license, visit https://creativecommons.org/licenses/by/4.0/. This license does not apply to figures/photos/artwork or other content included in the article that is credited to a third party; obtain authorization from the rights holder before using such material. Study limited to analysis of aggregated, publicly available data not requiring regulatory approval. All data used in this work came from covid19india.org, based, in part, on data from the Indian Ministry of Health and Family Welfare. **Funding:** The research was supported by internal funding from the University of Michigan School of Public Health and Michigan Institute of Data Science. **Author contributions:** Conceptualization: B.M., L.G., R.S., and S.S. Methodology: M.S., S.P., M.K., R.B., R.K., L.G., R.S., S.S., and B.M. Investigation: M.S., S.P., R.K., L.Z., D.R., and B.M. Visualization: M.S., S.P., and M.K. Funding acquisition: B.M. Project administration: M.S. and B.M. Supervision: B.M., S.S., L.G., and R.S. Writing (original draft): R.B., S.P., L.Z., D.R., M.S., A.H., and B.M. Writing (review and editing): M.S., S.P., L.Z., D.R., L.G., A.H., R.S., and B.M. **Competing interests:** The authors declare that they have no competing interests. **Data and materials availability:** All data needed to evaluate the conclusions in the paper are present in the paper and/or the Supplementary Materials. We use data on reported infected cases and COVID-19–attributed deaths through 30 June 2021 from covid19india.org (*16*). Population data for India were obtained from the Unique Identification Authority of India (Aadhar) (*74*). Analysis codes are available via Zenodo at www.doi.org/10.5281/zenodo.6514992.

Submitted 2 March 2022
Accepted 4 May 2022
Published 17 June 2022
10.1126/sciadv.abp8621
