# Supplementary material for: Lessons from SARS-CoV-2 in India: A data-driven framework for pandemic resilience
Source: Sci Adv. 2022 Jun 17;8(24):eabp8621. doi: 10.1126/sciadv.abp8621 (PMC9205583; doi:10.1126/sciadv.abp8621)
Supplement: Supplementary file 2 — Sections S1 to S6 Tables S1 to S16 Figs. S1 to S16 References [file sciadv.abp8621_sm.pdf]

Science Advances
AAAS

Supplementary Materials for

Lessons from SARS-CoV-2 in India: A data-driven framework for pandemic resilience

Maxwell Salvatore *et al.*

Corresponding author: Bhramar Mukherjee, bhramar@umich.edu

Sci. Adv. **8**, eabp8621 (2022)
DOI: 10.1126/sciadv.abp8621

The PDF file includes:

Sections S1 to S6
Tables S1 to S16
Figs. S1 to S16
References

Other Supplementary Material for this manuscript includes the following:

Data file S1

Section 1. Methods for Descriptive Comparison of Waves 1 and 2 in India

These timelines provide a broad summary of the types of interventions implemented in Maharashtra during the second wave (**Table S1**) and India during the first and second waves of COVID-19 (**Table S2**). Mapping of public health interventions (PHI) implemented during the specific time periods in Maharashtra that correspond to the intervention effects used in our modeling exercise (i.e., $\pi(t)$ schedule in the eSAIR model) are described in **Table S3**.

The references for **Table S1** are available in the supplemental reference **Data File S1**.

Table S1. Timeline of COVID-19 interventions in **Maharashtra** during December 31, 2020-July 31, 2021

Time Period: December 31, 2020 – July 31, 2021	
Date	***Intervention***
December 31, 2020	• Partial lockdown measures such as curbs on re-opening of offices, swimming pools, interstate activities, public transportation, schools/colleges and ban on social/religions/political gatherings to be continued until January 31, 2021[1]
January 29	• Ongoing partial lockdown extended until February 28, 2021[2]
February 10	• Government of Maharashtra extends travel restrictions for travelers coming from Kerala, and a negative test report is mandated. Similar travel restrictions had been instituted in four states (Delhi, Rajasthan, Goa, Gujarat) since November 23, 2020[3]
February 15	• In-person classes in colleges and universities reopen with restricted capacity start after >10 months of closure[4]
February 18	• Spike in cases over the prior few days and the biggest single-day spike in cases in the last 70 days lead to restrictions on movement of people in 7 districts of Maharashtra[5]<br>• Authorities in some districts impose partial lockdowns[6]
March 10	• To curb a COVID-19 surge, Maharashtra districts are asked to ramp up measures like identifying micro-containment zones, increasing contact tracing, and disallowing weddings and other large gatherings[7]
March 22	• Entry into shopping malls without a negative test report is prohibited[8]
March 28	• Statewide night curfew is imposed[9]
April 2	• Testing capacity and hospital bed capacity are increased[10]
April 9	• Complete weekend lockdown, in addition to night curfew, is imposed across the state[11]<br>• All shops (except essential ones), malls, and markets are to be shut until April 30[12]
April 14	• Maharashtra Chief Minister declares a 15-day curfew from April 14 until May 1. All establishments, public places, and services (except essential services) are to remain closed, but inter-district travel is allowed.[13]
April 17	• School certificate exams are deferred or cancelled[14]
April 22	• Maharashtra goes into a complete lockdown mode after following curfew for a week[15]
April 23	• Restrictions are placed on inter-state and inter-district travel[16]
April 29	• Maharashtra extends the lockdown, with exemption of essential services until May 15[17]
May 13	• The lockdown is extended until June 1, with stricter restrictions implemented at the district level wherever cases are on the rise[18]<br>• Maharashtra plans to create a separate database of mucormycosis ('black fungus') cases to assess its frequency during the second wave of COVID-19[19]
May 27	• Districts with poor vaccination rates are prioritized to get more vaccine doses[20]<br>• State government announces a pediatric task force in preparation for the next wave[21]
May 31	• The lockdown extended until June 15 but with relaxation of restrictions in districts showing reduced test positive rates and occupancy of hospital beds with oxygen support[22]

June 7	◆ 5-level unlocking plan starts across Maharashtra; levels of relaxation of restrictions are defined based on an area's test positivity rate and availability of hospital beds with oxygen[23]
June 28	◆ Maharashtra increases restrictions after cases from Delta+ variants rise; 5-level unlocking plan is reduced to a 3-level unlock plan, with the first 2 levels with maximum relaxations removed[24]
July 15	◆ Schools for classes (grades) 8 to 12 to reopen in non-covid zones with low COVID-19 transmission[25]
July 26	◆ State government relaxes some rules this week for districts with a low test positivity rate[26]

In **Table S2**, Wave 2 is defined as starting from February 19, 2021, when the 7-day trailing average national effective reproduction number for COVID-19 in India, R_t, crossed unity. Wave 1 is defined as starting on the same day the daily case count first reached or surpassed the daily case count at the start of Wave 2 (13,916 daily cases on February 19, 2021), which first happened on June 19, 2020. Entries (with exceptions cited) for Wave 1 are updated from Table 1 in Ray et al., 2020 (*5*).

The references for **Table S2** are available in the supplemental reference **Data File S1**.

Table S2. Timeline of COVID-19 interventions in India during the first and second waves.

Wave 1: June 19, 2020 – February 18, 2021	
Date	***Intervention***
Jul 1, 2020	◆ Central government announces guidelines for second phase of lifting countrywide lockdown, referred to as Unlock 2.0
Jul 15, 2020	◆ Covaxin, an Indian-based COVID-19 vaccine, enters phase I of clinical trials
Jul 25, 2020	◆ Central government issues guidelines for third phase of lifting countrywide lockdown restrictions, referred to as Unlock 3.0
Aug 26, 2020	◆ Covishield, an Oxford-AstraZeneca vaccine, enters clinical trials
Aug 29, 2020	◆ Central government announces guidelines for fourth phase of lifting countrywide lockdown, referred to as Unlock 4.0
Sep 30, 2020	◆ Central government announces guidelines for fifth phase of lifting countrywide lockdown, referred to as Unlock 5.0
Oct 5, 2020	◆ Central government states that 200-250 million people will be vaccinated by July 25, 2021
Oct 26, 2020	◆ Central government requests preparation of 3-tier approach to vaccine program
Nov 10, 2020	◆ Delhi experiences third wave of COVID-19 pandemic
Dec 21, 2020	◆ India imposes air travel ban on flights from the United Kingdom
Dec 29, 2020	◆ Six passengers from the United Kingdom tested positive for new B.1.1.7 lineage of SARS-CoV-2
Dec 30, 2020	◆ Numerous cities issue a night curfew
December 31, 2020	◆ Maharashtra issues partial lockdown measures such as curbs on re-opening of offices, swimming pools, interstate activities, public transportation, schools/colleges and ban on social/religions/political gatherings to be continued until January 31, 2021[1]
Jan 16, 2021	◆ Nationwide vaccination program begins with initial prioritization of healthcare workers[2]

Jan 19, 2021	◆ Lakshadweep, an island territory off the coast of Kerala, is the last region in India to report first case[3]
January 29	◆ Maharashtra extends ongoing partial lockdown until February 28, 2021[4(p28)]
February 10	◆ Maharashtra government extends travel restrictions for travelers coming from Kerala, and a negative test report is mandated. Similar travel restrictions had been instituted in four states (Delhi, Rajasthan, Goa, Gujarat) since November 23, 2020[5]
February 15, 2021	◆ In Maharashtra, in-person classes in colleges and universities reopen with restricted capacity start after >10 months of closure[6]
February 18, 2021	◆ Maharashtra sees spike in cases over the prior few days and the biggest single-day spike in cases in the last 70 days lead to restrictions on movement of people in 7 districts of Maharashtra[7]<br>◆ Authorities in some districts impose partial lockdowns[8(p000)]
Wave 2: February 19, 2021 – July 31, 2021	
Date	***Intervention***
March 10, 2021	◆ To curb a COVID-19 surge, Maharashtra districts are asked to ramp up measures like identifying micro-containment zones, increasing contact tracing, and disallowing weddings and other large gatherings[9]
March 22, 2021	◆ In Maharashtra, entry into shopping malls without a negative test report is prohibited[10]
March 28, 2021	◆ Maharashtra imposes statewide night curfew[11]
April 1, 2021	◆ Central government extends vaccine eligibility to adults aged ≥ 45, as announced on March 23[12]
April 2, 2021	◆ In Maharashtra, testing capacity and hospital bed capacity are increased[13]
April 5, 2021	◆ Maharashtra issues restrictions on malls, restaurants, religious buildings, and cinemas, as announced on April 4[14]
April 8, 2021	◆ Six states at least, including Andhra Pradesh, Chhattisgarh, Haryana, Maharashtra, Odisha and Telangana, report vaccine shortages[15]<br>◆ Madhya Pradesh imposes night curfew in all urban areas, as announced on April 7[16]
April 9, 2021	◆ Maharashtra imposes complete weekend lockdown, in addition to night curfew, statewide[17]<br>◆ In Maharashtra, all shops (except essential ones), malls, and markets are to be shut until April 30[18]<br>◆ Jammu and Kashmir imposes night curfew in urban regions across eight districts, as announced April 8[19]<br>◆ Maharashtra suspends vaccine administration in multiple districts[20]<br>◆ Chhattisgarh district of Raipur issues lockdown until April 19, as announced on May 7[21]
April 10, 2021	◆ Maharashtra enters statewide weekend lockdown, as announced on April 4,[14] which was extended until June 15[22]<br>◆ Karnataka imposes night curfew until April 20, as announced on April 9[23]
April 14, 2021	◆ Maharashtra Chief Minister declares a 15-day curfew from April 14 until May 1. All establishments, public places, and services (except essential services) are to remain closed statewide, but inter-district travel is allowed[24]
April 16, 2021	◆ Delhi imposes a weekend curfew, as announced on April 16[25]<br>◆ Karnataka imposes restrictions on public gatherings and entertainment activities, as announced on April 16[23]
April 17, 2021	◆ In Maharashtra, school certificate exams are deferred or cancelled[26]
April 19, 2021	◆ Delhi imposes a week-long lockdown until April 26, as announced on April 19,[25] which was extended until June 7[22]
April 20, 2021	◆ Telangana issues an immediate statewide night curfew, as announced on April 20[27]

April 22, 2021	◆ Jharkhand imposes lockdown-like restrictions until April 29, as announced on April 20[28]<br>◆ Maharashtra goes into a complete lockdown mode after following curfew for a week[29]
April 23, 2021	◆ Puducherry imposes statewide lockdown, as announced on April 21,[30] which was extended until June 7[22]<br>◆ Maharashtra places restrictions on inter-state and inter-district travel[31]
April 24, 2021	◆ Andhra Pradesh issues a statewide night curfew, as announced on April 23[32]<br>◆ Uttar Pradesh issues a statewide weekend lockdown, as announced on April 20,[33] which was extended until June 1[22]
April 27, 2021	◆ Assam imposes statewide night curfew through May 7, as announced on April 27[34]<br>◆ Himachal Pradesh announces night curfew across 1/3 of all districts and issues weekend lockdown, as announced on April 25[35]<br>◆ Karnataka imposes a two-week statewide close down, as announced on April 26[36]
April 29, 2021	◆ Maharashtra extends the lockdown, with exemption of essential services until May 15[37]
April 30, 2021	◆ Haryana issues a weekend curfew across nine districts, as announced on April 30[34]<br>◆ West Bengal issues restrictions including a ban on gatherings as well as shutdown of cinemas, gyms, malls and restaurants, as announced on April 30[34], which were extended until June 15[22]<br>◆ Nagaland enters statewide lockdown until May 14, as announced on April 27,[38] which was extended until June 11[39]
May 1, 2021	◆ Central government extends vaccine eligibility to adults aged ≥ 18[40]
May 3, 2021	◆ Haryana imposes a statewide lockdown through May 10, as announced on May 3,[34] which was extended until June 7[22]<br>◆ Select districts in Mizoram, including Aizawl, enter an eight-day lockdown, as announced on May 2,[41] which was extended until June 6[42]<br>◆ Punjab issues a weekend lockdown and a night curfew through May 15, as announced on May 3,[43] which was extended until June 10[22]
May 5, 2021	◆ Bihar issues statewide lockdown until May 15, as announced on May 4,[34] which was extended until June 8[22]<br>◆ Odisha enters a 14-day statewide lockdown until May 19, as announced on May 2,[44] which was extended until June 17[45]<br>◆ Andhra Pradesh enters a statewide 14-day partial curfew, as announced on May 3,[46] which was extended until June 10[22]
May 7, 2021	◆ Gujarat issues night curfew on twenty-nine cities, and travel restrictions as well as social distancing measures through April 30, as announced May 7,[47] which was extended until June 4[22]
May 8, 2021	◆ Kerala enters a statewide lockdown until May 16, as announced on May 6,[34] which was extended until June 9[22]
May 9, 2021	◆ Goa imposes a statewide curfew until May 23, as announced on May 7[34]
May 10, 2021	◆ Karnataka imposes a statewide lockdown until May 24, as announced on May 7,[34] which was extended until June 7[22]<br>◆ Rajasthan enters a statewide lockdown until May 24, as announced on May 6,[34] which was extended to June 2[22]<br>◆ Tamil Nadu issues a statewide lockdown until May 24, as announced on May 8,[34] which was extended until June 7[22]
May 12, 2021	◆ Telangana imposes statewide lockdown, as announced on May 11, [48] which was extended until June 9[22]
May 13, 2021	◆ Maharashtra lockdown is extended until June 1, with stricter restrictions implemented at the district level wherever cases are on the rise[49(p1)]<br>◆ Maharashtra plans to create a separate database of mucormycosis (‘black fungus’) cases to assess its frequency during the second wave of COVID-19[50]

May 23, 2021	◆ Rajasthan extends lockdown until June 8, as announced on May 23[51]
May 27, 2021	◆ Maharashtra districts with poor vaccination rates are prioritized to get more vaccine doses[52(p19)]<br>◆ Maharashtra state government announces a pediatric task force in preparation for the next wave[53]
May 31, 2021	◆ The lockdown extended until June 15 but with relaxation of restrictions in districts showing reduced test positive rates and occupancy of hospital beds with oxygen support[54(p15)]
June 5, 2021	◆ Himachal Pradesh extends night curfew statewide until June 14, as announced on June 5[55]
June 7, 2021	◆ Across Maharashtra, 5-level unlocking plan starts; levels of relaxation of restrictions are defined based on an area's test positivity rate and availability of hospital beds with oxygen[56]
June 8, 2021	◆ Madhya Pradesh extends full lockdown through June 15, as announced on June 8, whilst allowing markets in Ujjain to remain open with a curfew and requiring shop employees to be vaccinated. Weekend curfew remains in effect for Bhopal[57]<br>◆ Rajasthan relaxes lockdown restrictions from June 8, as announced on June 8[58]
June 9, 2021	◆ Bihar lifts full lockdown from June 9, as announced on June 9, with certain restrictions remaining[59]
June 27, 2021	◆ Rajasthan further eases restrictions from June 28, whilst mandating vaccination of shop employees and the allowance of those vulnerable to work remotely (e.g., those whom are pregnant or have comorbidities, etc.), as announced on June 27[60]
June 28, 2021	◆ Maharashtra increases restrictions after cases from Delta+ variants rise; 5-level unlocking plan is reduced to a 3-level unlock plan, with the first 2 levels with maximum relaxations removed[61]
July 1, 2021	◆ Telangana extends restrictions through July 31, as announced on July 1[62]
July 15, 2021	◆ In Maharashtra, schools for classes (grades) 8 to 12 to reopen in non-covid zones with low COVID-19 transmission[63]
July 25, 2021	◆ Jammu and Kashmir relaxes restrictions by lifting statewide weekend curfew, while night curfew remains in place, as announced on July 25[64]
July 26, 2021	◆ Maharashtra state government relaxes some rules this week for districts with a low test positivity rate[65]
July 27, 2021	◆ Chhattisgarh extends lockdown through August 6, as announced on July 27[66]
July 30, 2021	◆ Jharkhand extends restrictions and re-opens schools for grades 9-12, as announced July 31[67]<br>◆ Nagaland extends lockdown measures through August 31, as announced on July 30, with Kohima and 2 other cities under full lockdown[68]
July 31, 2021	◆ Karnataka extends restrictions through August 16, as announced on July 31, and announces target of having 72% of teachers vaccinated[69]<br>◆ Punjab plans to re-open school for all classes on August 2, as announced on July 31[70]

Section 2. Methods for Characterizing the Potential Effect of Public Health Interventions

Section 2.1.1. Details of the eSAIR model

We implement a modified version of the traditional (S)usceptible-(A)ntibody-(I)nfected-(R)ecovered (SAIR) model (*81*), called an extended SAIR (or eSAIR) model (*76*). The eSAIR framework assumes that the true underlying probabilities of the four compartments follow a latent Markov transition process, and that we only observe a fraction of the daily new infected cases and removed. We assume that the observed proportions of infected and removed cases on day *t* are denoted by Y_t^I and Y_t^R, respectively, and further note that the true underlying probabilities of the S, A, I, and R compartments always add up to unity, i.e., $\theta_t^S + \theta_t^A + \theta_t^I + \theta_t^R = 1$ for all t. This model can then be described by the system of differential equations as presented in **Figure S1**.

Description of compartmental model and underlying transmission dynamics

Observed layer

Latent layer

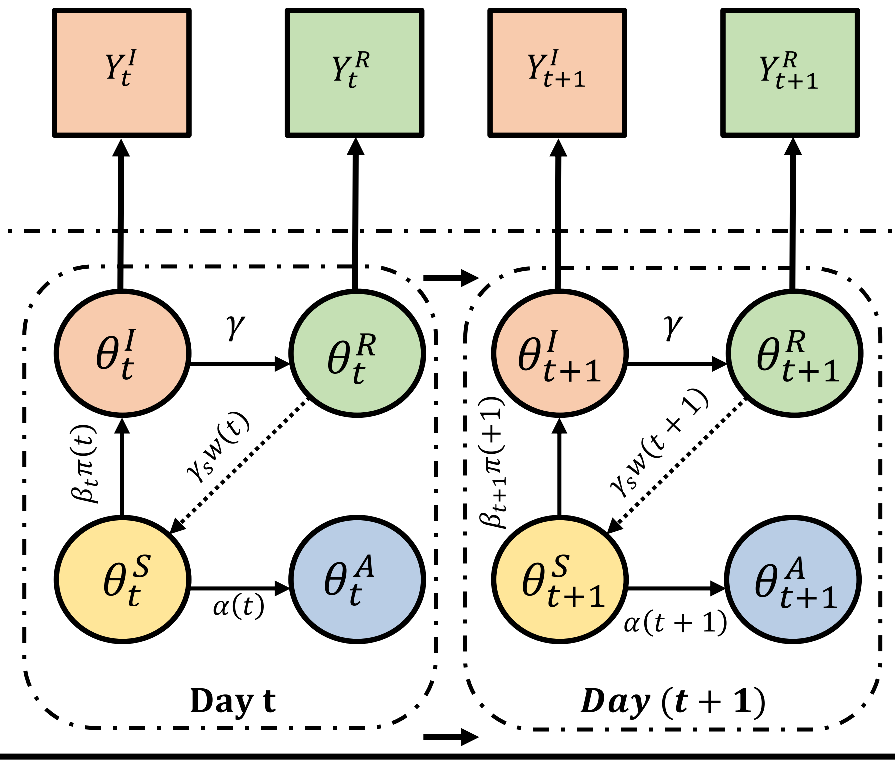

Model for observed proportions (*Y*) given latent proportions (*θ*):

$$Y_t^I | \boldsymbol{\theta_t}, \boldsymbol{\tau} \sim Beta\left(\lambda^I \theta_t^I, \lambda^I (1-\theta_t^I)\right)$$
$$Y_t^R | \boldsymbol{\theta_t}, \boldsymbol{\tau} \sim Beta\left(\lambda^R \theta_t^R, \lambda^R (1-\theta_t^R)\right)$$

System of equations: $f(\boldsymbol{\theta_t}, \boldsymbol{\beta_t}, \boldsymbol{\pi(t)}, \boldsymbol{\alpha(t)}, \boldsymbol{\gamma}, \boldsymbol{\gamma_s})$

$$\frac{d\theta_t^S}{dt} = -\beta_t \pi(t) \theta_t^S \theta_t^I - \alpha(t)\theta_t^s + w(t)\gamma_s \theta_t^R$$
$$\frac{d\theta_t^I}{dt} = \beta_t \pi(t) \theta_t^S \theta_t^I - \gamma \theta_t^I$$
$$\frac{d\theta_t^R}{dt} = \gamma \theta_t^I - w(t)\gamma_s \theta_t^R$$
$$\frac{d\theta_t^A}{dt} = \alpha(t)\theta_t^s$$

In this extended Susceptible-Antibody-Infected-Removed (e-SAIR) model, we allow for a compartment A that contains the fraction of population with antibodies, determined by $\alpha(t)$, the proportion of seropositive individuals at time t. The model allows for a time-varying transmission rate β_t that takes the presence of different strains with differing transmission rates into account. Further, the effective reproduction number R_t is modified through an intervention schedule π to account for the effect of a public health intervention (PHI). To account for waning immunity, the $w(t)\gamma_s$ factor captures the process of reinfection. The model assumes that the true underlying compartment-specific probabilities follow a latent Markov transition process. The observed proportions in compartment X (say) on day t is given by Y_t^X, with true underlying probability θ_t^X. The transmission model is described by the set of equations $f(\boldsymbol{\theta_t}, \beta_t, \alpha(t), \pi(t), w(t), \gamma, \gamma_s)$ above, where γ and γ_s denote the disease removal and reinfection rates, respectively. A Markov process is used to build the vector $\boldsymbol{\theta_t}$ of the underlying probabilities of the three compartments at time t, whose mean is modeled as an unknown function of $\boldsymbol{\theta_{t-1}}$, along with some transition parameters like λ^I, λ^R and κ which control the variability of the observed and latent processes. The table below describes the parameters and their hyperparameter choices in greater detail.

	Parameter		Description
	**Original prior**	**Prior for sensitivity analysis**	
Transmission dynamics	$\boldsymbol{\theta}_0 \sim$ Dirichlet$(1 - Y_0^I - Y_0^R, Y_0^I, Y_0^R)$		Underlying probabilities of the three compartments on the first day, i.e., $t = 0$. Y_0^X denotes the observed proportions in compartment X on the first day when data is available.
	$R_0 \sim$ Lognormal$(0.582,\ 0.223)$	$R_0 \sim$ Lognormal$(1.590,\ 0.039)$	Effective reproduction number at $t = 0$. Hyperparameter values were so chosen such that for the original (modified) prior (*41*) we have $E(R_0) = 2$ (5) and $SD(R_0) = 1$.
	$\gamma \sim$ Lognormal$(-2.955,\ 0.910)$		Hyperparameter values are so chosen such that the average infectious period is given by $1/E(\gamma) = 12$ (days) and $SD(\gamma) = 0.1$ (*5*). The prior mean of γ indicates an average infectious period of 12 days, which is originally set using the estimation from SARS outbreak in Hong Kong (*82*) due to the similarity between the two viruses; and this value also aligns well with a few 2020 studies on COVID-19 in China (*83*).
	$\gamma_s \sim$ Lognormal$(-8.660,\ 6.358)$		Hyperparameter values are so chosen such that the average immunity period is given by $1/E(\gamma_s) = 8$ (months) and $SD(\gamma_s) = 0.1$. The prior mean for the average immunity period is based on a recent study (*79*).
	$w(t) \sim$ Rayleigh(310)		The time-dependent waning of immunity $\omega(t)$ is modeled as a Rayleigh distribution (σ=310) to ensure 50% individuals are still immune after 1 year (*84*).
Variability	$\kappa,\ \lambda^I,\ \lambda^R \sim$ Gamma$(2,\ 0.0001)$		Hyperparameter values are so chosen such that the variability parameters have large variances in both observed and latent processes, which may be adjusted over the course of epidemic with more data becoming available.

Figure S1. The compartments and transitions for the eSAIR model used.

Here, $\beta_t > 0$ denotes the time-varying disease transmission rate, $\gamma > 0$ denotes the removal rate, $\gamma_s > 0$ denotes the re-infection rate, $\alpha(t)$ denotes the seroprevalence at time t and $w(t)$ acts as a time-varying waning immunity modifier of γ_s. The basic reproduction number $R_0 \coloneqq \beta_0/\gamma$ indicates the expected number of cases generated by one infected case in the absence of any intervention and assuming that the whole population is susceptible. At this stage, for the observed infected and removed proportions, we assume a Beta-Dirichlet state-space model, independent conditionally on the underlying process:

$$Y_t^I | \boldsymbol{\theta_t}, \boldsymbol{\tau} \sim Beta\big(\lambda^I \theta_t^I, \lambda^I (1 - \theta_t^I)\big),$$

$$Y_t^R | \boldsymbol{\theta_t}, \boldsymbol{\tau} \sim Beta\big(\lambda^R \theta_t^R, \lambda^R (1 - \theta_t^R)\big).$$

Further, the Markov process on the latent proportions is built as:

$$\boldsymbol{\theta_t} | \boldsymbol{\theta_{t-1}}, \boldsymbol{\tau} \sim Dirichlet\big(\kappa f(\boldsymbol{\theta_{t-1}}, \beta_{t-1}, \gamma)\big)$$

where $\boldsymbol{\theta_t}$, whose mean is modeled as an unknown function of the probability vector from the previous time point, along with the transition parameters; $\boldsymbol{\tau} = (\beta_t, \gamma, \gamma_s, \boldsymbol{\theta_0^T}, \boldsymbol{\lambda}, \kappa)$ denoting the whole set of parameters where λ^I, λ^R and κ are parameters controlling variability of the observation and latent process, respectively.

Prior specifications: We follow the same MCMC scheme as in the original eSIR package. **Figure S2** presents a summary of the priors we utilize in the model and relevant hyperparameters. Specifically, our priors are as follows.

- $\boldsymbol{\theta}_0 \sim$ Dirichlet$\big((1 - Y_0^I - Y_0^R - Y_0^A), Y_0^A, Y_0^I, Y_0^R\big)$. Y_0^X denotes the observed proportions in compartment X on the first day when data is available. While we do not directly observe the A compartment, we incorporate information from serosurveys carried out in India to plug in a reasonable estimate of Y_0^A.
- $R_0 \sim$ LogNormal$(0.582, 0.223)$. This is chosen so that the mean reproduction number $E(R_0) = 2$, and $SD(R_0) = 1$. This prior mean, 2.0, is approximately the average of the estimates from many other (wave 1) COVID-19 studies on the Indian population (*85*–*89*). A modified lognormal prior which ensures $E(R_0) = 5$, and $SD(R_0) = 1$ was used in some analyses as well (*41*).
- $\gamma \sim$ LogNormal$(-2.955, 0.910)$. This is chosen such that the average infectious period is $\frac{1}{E(\gamma)} = \frac{1}{0.082} \approx 12$ days, and $SD(\gamma) = 0.1$ (*5*). This prior mean indicates an average infectious period of 12 days, which is originally set using the estimation from SARS outbreak in Hong Kong (*82*) due to the similarity between the two viruses; and this value also aligns well with a few 2020 studies on COVID-19 in China (*83*, *90*, *91*).
- $\beta = R_0 \gamma$.
- $\kappa, \lambda^I, \lambda^R \sim iid\ Gamma(2,\ 0.0001)$. It is customary for these variability parameters to set large variances in both observed and latent processes, which may be adjusted over the course of epidemic with more data becoming available.

Specifying the A compartment: To account for information arising from the four national level serosurveys conducted serially in India as they relate to the proportion of population in compartment A, we consider the following approach. The national serosurveys (*17*, *92–94*) were conducted by the government run Indian Council of Medical Research (ICMR). These surveys revealed a seroprevalence of 0.73% in June 2020, 7.1% in September 2020, 24.1% in February 2021 and 67.6% in July 2021. We use information presented in the nationwide serological surveys to determine the proportion of population in compartment A at time t or θ_A^t. As is seen in **Figure S2**, in absence of any further information, the trajectory of seroprevalence over time may be studied by means of a piecewise-linear time-varying curve. The fitted curve is generated by setting knots at the second and third serosurvey dates (i.e., September 1, 2020, and February 1, 2021). We use this curve to find the estimated seroprevalence at a given time t, which we use as our choice of input for $\alpha(t)$.

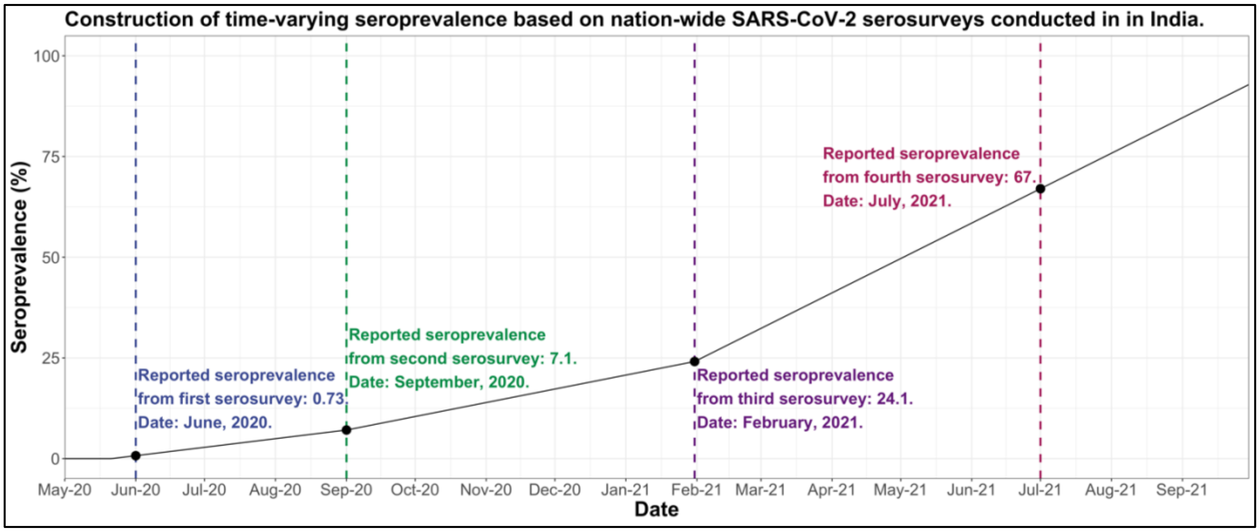

Figure S2. A graph depicting piecewise linear growth in COVID-19 seroprevalence estimates in India from June 2020 through July 2021. The first (*92*), second (*93*), third (*17*), and fourth (*94*) national seroprevalence estimates (as a percentage) are shown in blue, green, purple, and red, respectively.

Choice of time-varying transmission parameter β_t: We additionally need to specify β_t. We construct the time-varying overall transmission rate by considering the top three dominant strains, namely the ancestral strain, with a transmission rate of $\beta^{(1)}$, the Alpha variant, with a 50% increase in transmissibility given by $\beta^{(2)} = 1.5 \times \beta^{(1)}$ (*77*), and the Delta variant, with a 150% increase in transmissibility, given by $\beta^{(3)} = 2.5 \times \beta^{(1)}$ (*41*). Based on INSACOG data (*78*), the relative prevalence of the ancestral, Alpha and Delta strains at time t are given by $p_1(t), p_2(t)$ and $p_3(t)$ respectively. Note that in this formulation $\beta^{(1)}$ is the unknown key parameter. We construct the overall time-varying transmission rate as a weighted average: $\beta^*(t) = \sum_{i=1}^{3} p_i(t)\beta^{(i)}$, obtain estimates of this quantity from the posterior draws of $\beta^{(1)}$and then apply a LOESS-smoother with span 0.25 to construct a smoothed version of $\beta^*(t)$, namely β_t, which we use in our analyses along with corresponding smoothed credible intervals (**Figure S3**).

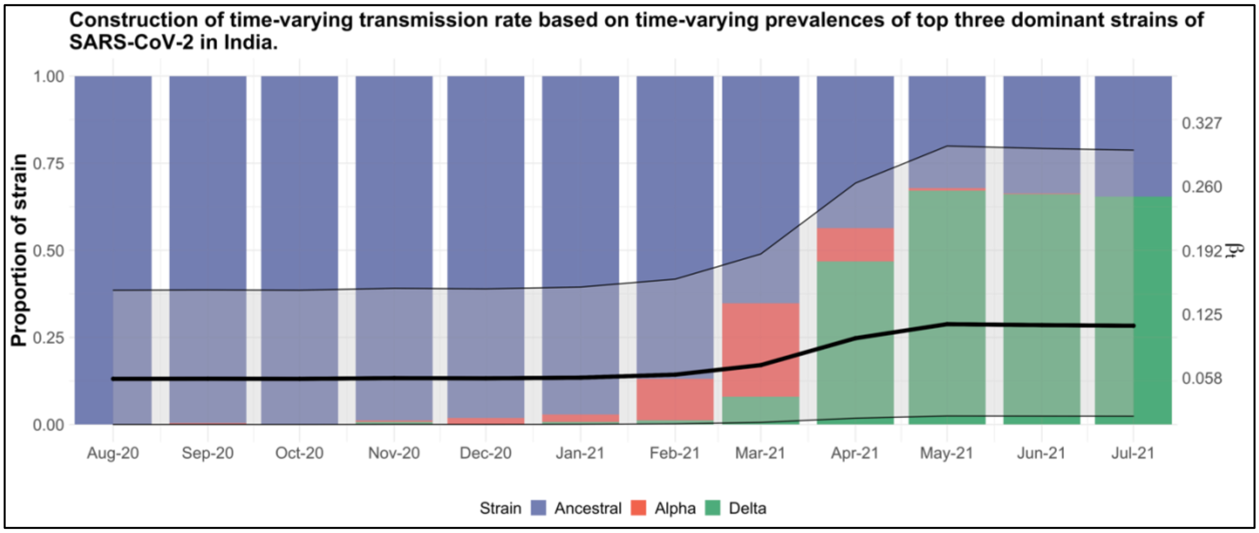

Figure S3. The posterior mean and 95% credible interval of the time-varying effective transmissibility parameter, β_t used in our analyses in the main text. It is estimated using the relative proportion of COVID-19 strains in India based on data from INSACOG. The Alpha and Delta strains are estimated to be 50% (*77*) and 150% (*41*) more transmissible than the ancestral strain, respectively. The posterior estimates are then smoothed using a LOESS smoother (span = 0.25).

Choice of the Intervention Schedule $\pi(t)$: The primary challenge for obtaining a realistic lockdown intervention is the choice of an appropriate underlying intervention schedule or "$\pi(t)$ schedule" in the eSIR modeling vocabulary. We make use of three actual PHI scenarios from India from which we derive a realistic $\pi(t)$ schedule, namely: (1) interventions implemented from March 28, 2021, to April 13, 2021, in the weeks immediately preceding the lockdown in Maharashtra, which we refer to as **strengthened PHI (non-lockdown) effect,** (2) the Maharashtra lockdown beginning on April 14, 2021, in response to the second wave, **a moderate lockdown effect**; and (3) the national lockdown beginning on March 25, 2020, in response to the first wave, a **strong lockdown effect** (presented only in supplement)**. Figure 2** in the main text describes our approach to deriving a $\pi(t)$ schedule using key intervention dates from time-varying effective reproduction number R_t, which is calculated from the daily case count time series.

Figure 2A shows daily COVID-19 case counts in Maharashtra from February 15, 2021, to July 31, 2021. The vertical dashed lines correspond to key PHI dates. March 28, 2021, corresponds to the day Maharashtra instituted a statewide suite of non-lockdown PHI. April 14, 2021, corresponds to the day Maharashtra instituted a statewide lockdown. June 7, 2021, corresponds to the day Maharashtra began lifting the lockdown measures.

The daily case counts are used to calculate the time-varying effective reproduction number, R_t, as shown in **Figure 2B**. To do this, we used the `estimate_R` function from the EpiEstim package in R, with the following settings: method = "parametric_si"; mean_si = 7; std_si = 4.5.

We present an example $\pi(t)$ schedule in **Figure 2C**. This is the ratio of time-varying R_t over the trailing 7-day average R_t prior to the start of the intervention. In **Figure 2C**, the intervention begins on March 28, 2021, so the denominator of this ratio is the average R_t

estimate from March 21, 2021, through March 27, 2021. The resulting vector is then smoothed using a LOESS smoother with span = 1. This $\pi(t)$ schedule represents the institution of non-lockdown PHI followed 18 days later by the institution of lockdown measures, which are then gradually lifted beginning 55 days after the lockdown begins.

Figure 2D shows the four $\pi(t)$ schedules used in our models: moderate PHI (non-lockdown) in orange, strengthened PHI (non-lockdown) in red, moderate lockdown in purple, and strong lockdown in black.

The **strengthened PHI (non-lockdown) effect** – estimated from the pre-lockdown intervention implemented from March 28, 2021, to April 13, 2021, in Maharashtra– had a more subdued impact relative to the 2021 Maharashtra lockdown. We carried forward the last raw $\pi(t)$ estimate (i.e., $\pi(t)$ on April 13, 2021) out such that the $\pi(t)$ vector had length 100. This resulting raw $\pi(t)$ schedule was than smoothed using a LOESS smoother with span = 1 to achieve the strengthened PHI (non-lockdown) effect represented by the red line in **Figure 2D**.

The strengthened PHI (non-lockdown) effect comprises stringent interventions that likely would not have been feasible to implement nationally very early in Wave 2. However, we wanted to evaluate whether less intensive interventions that might have been feasible to implement very early in Wave 2 could have mitigated cases and deaths. We therefore derived a **moderate PHI (non-lockdown) effect** by reducing the effect of the strengthened PHI (non-lockdown) effect by 20% (i.e., $\min(\pi(t) * 1.2, 1)$).

The **moderate lockdown effect** is estimated from the statewide lockdown in Maharashtra starting April 14, 2021, through August 15, 2021. This schedule incorporates the initial 55-day lockdown period and the subsequent, gradual lifting of lockdown restrictions beginning June 7, 2021. The **strong lockdown effect** – estimated from the 2020 nationwide lockdown – was swift and resulted in immediate and long-term reductions in R_t, representing the strongest intervention effect of any of our scenarios. As with the moderate lockdown effect, the strong lockdown effect incorporates the subsequent unlock periods and covers the 200 days from the institution of the lockdown – March 27, 2020 – through October 12, 2020.

Given that we derive intervention effects for the $\pi(t)$ schedule from COVID-19 data during specific time periods in Maharashtra or India, we are able to specify the types of PHI implemented in each setting that may have contributed to the observed intervention effects (**Table S4**). We obtained information on the specific PHI implemented during each time period by searching articles published in the Indian media and government documents. For example, from March 28, 2021, to April 13, 2021, in Maharashtra, an array of PHI were implemented in rapid succession—including closure of restaurants and houses of worship, workplace and store capacity reductions, night curfew, and weekend lockdowns—which may explain the observed strengthened PHI (non-lockdown) effect. We also note differences in implementation of the 2021 Maharashtra lockdown and 2020 national lockdown in India—such as more comprehensive suspension of public and private transportation during the national lockdown—that may explain the larger observed

effect of strong lockdown as compared to moderate lockdown (summarized in **Table S3** with detailed timeline of 2021 Maharashtra and 2020 India lockdowns in **Table S1** and **Table S2**, respectively).

Table S3. Modeled intervention effects and their correspondence to real world interventions implemented at different time periods and settings in India

**Intensity of intervention as described in our model**	Moderate PHI (non-lockdown)	Strengthened PHI (non-lockdown)	Moderate lockdown	Strong lockdown
**Time period and setting from which intervention effects are derived**	80% of the effect of early non-lockdown intervention	Interventions implemented in the Maharashtra state from March 28, 2021, to April 13, 2021	Lockdown implemented in Maharashtra state from April 14, 2021, to June 1, 2021	Lockdown implemented nationally in India from March 25, 2020, to May 2020
**Specific components of each intervention that was implemented**	Reduced effect is meant to estimate the potential impact of partial reductions in capacity in high-risk indoor settings and events, such as restaurants, bars, coffee/tea shops, cinemas, performance venues, weddings, and social, political, and religious gatherings.<br><br>We presume that low-risk settings such as workplaces, retail stores, and public transportation would be operating at normal capacity, in contrast to moderate non-lockdown interventions.	• Shut down of "90% of restaurants," but food pickup and delivery were allowed[1]<br>• Night curfew was implemented to reduce congregation in bars and restaurants[2]<br>• Ban on most large gatherings[3-5] but moderate limitations on weddings to 50 people.[1]<br>• Places of worship closed.[1]<br>• Workplace capacity was reduced, and private offices eventually closed; 50% capacity limits instituted in government offices[1]<br>• Capacity limits in malls and other shopping establishments[1] with eventual closure of all non-essential stores[6]<br>• Theaters and multiplexes remain closed.[1]<br>• Hair salons and beauty parlors to remain closed[6]<br>• Trains, buses, taxis, and autos allowed to operate at 50% capacity[1]<br>• Weekend lockdown (i.e., shelter at home)[1]<br>• Section 144 enforced: During the day cannot be seen in groups larger than 5 people[6]<br>• Schools and colleges remain closed[6]	All of the moderate non-lockdown interventions that were modeled previously in Maharashtra; however, in addition, the following were implemented:<br><br>• A shelter-at-home order outside of leaving for essential work or essential needs<br>• Eventual implementation of restrictions on interstate or interdistrict travel on April 23, 2021<br>• However, private transportation and public transportation "at seating capacity" on buses and trains were still allowed to operate to facilitate essential needs, essential services, and movement of essential goods[7]	As with moderate lockdown, all individuals in the country were ordered to shelter at home, outside of leaving for essential work or essential needs. However, the national lockdown included the following additional components that may have increased the observed intervention effect above that of moderate lockdown:<br><br>• Suspension of nearly all public and private transportation—locally, interdistrict, and interstate—except for transporting goods and emergency services[8]<br>• Anecdotally, greater intensity of enforcement of PHIs when compared to the Maharashtra state lockdown
**Date at which we implement a given intervention effect across India in our model**	February 19, 2021	March 13, 2021	March 19, 2021	March 19, 2021
**Correspondence of given intervention to our tiered Covid-19 framework (Table S14)**	Tier 2	Tier 3	Tier 4	Does not correspond to a tier in our Covid-19 response framework

The references for **Table S3** are available in the supplemental reference **Data File S1**.

We chose February 19, 2021, as the start date for the moderate PHI (non-lockdown) scenario because it was the first time the trailing 7-day average R_t crossed 1, and we wanted to evaluate the potential impact on COVID-19 cases and deaths if less intensive (and presumably more feasible) interventions had been implemented early in Wave 2. We chose March 13, 2021, as the start date for the strengthened PHI (non-lockdown) scenario because it was the first time the trailing 7-day average R_t crossed 1.2. We chose March 19, 2021, as the start date for the moderate lockdown scenario because it was the first time the trailing 7-day average R_t crossed 1.4. We summarize R_t, the PHI thresholds, and the trigger dates in **Figure S4**. To understand the impact of delayed intervention on COVID-19 cases and deaths, we also assessed the impact of moderate lockdown with later start dates: March 30, 2021, and April 15, 2021.

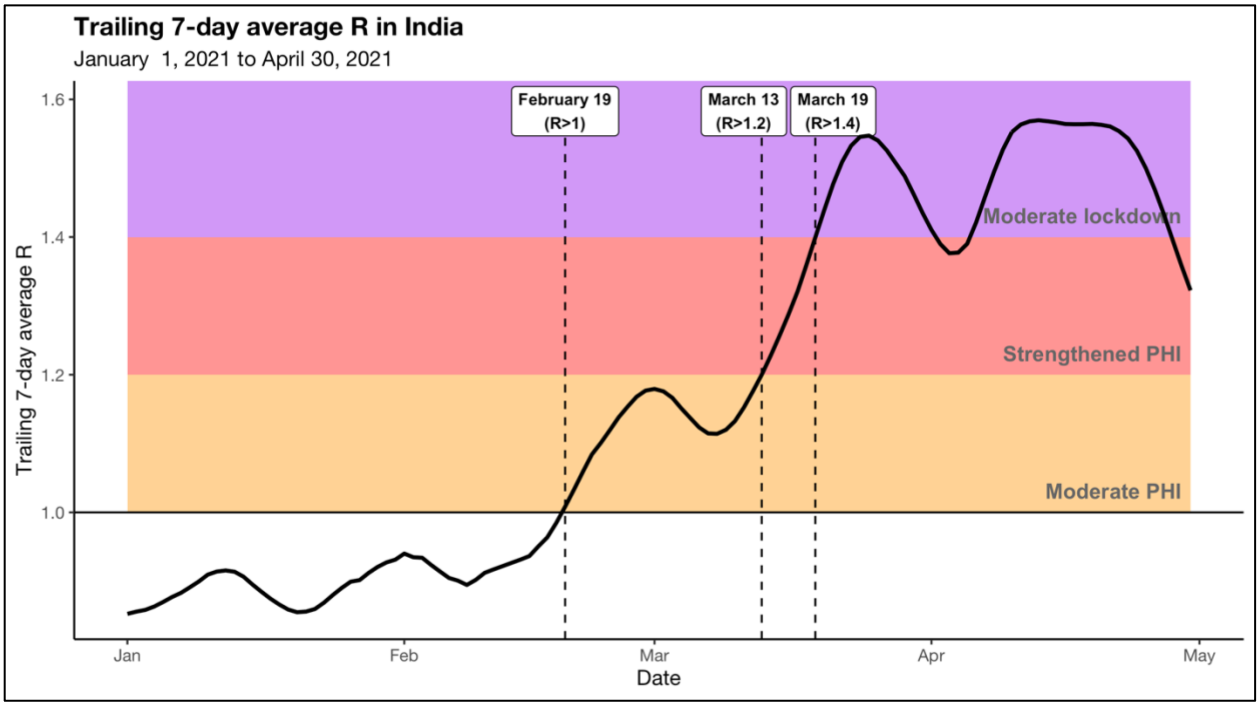

Figure S4. Time-series plot for the 7-day trailing average time-varying effective reproduction number, R_t, in India from January 1, 2021, through April 30, 2021. R_t is estimated using daily case counts and the estimate_R function from the EpiEstim package in R (specifying method = "parametric_si", mean_si = 7, std_si = 4.5). We have marked the dates that trigger our intervention scenarios: February 19, when the 7-day R_t crossed 1, triggering moderate PHI (non-lockdown; orange); March 13, when the 7-day R_t crossed 1.2, triggering strengthened PHI (non-lockdown; red); and March 19, when the 7-day R_t crossed 1.4, triggering a moderate lockdown (purple).

We project each scenario out to June 30, 2021, and compare the forecasted results to observed, reported counts on April 15, 2021, May 15, 2021, and June 15, 2021. For example, for the March 19, 2021, start date of the moderate lockdown, we apply the $\pi(t)$ schedule from March 19, 2021, until June 15, 2021. Note that on June 7, 2021, Maharashtra started lifting the statewide lockdown (**Table S1**). and that effect is incorporated in our current schedule.

Modeling re-infection through waning immunity: Following Faria et al., 2021, we model time-dependent waning of immunity as a Rayleigh distribution ($\sigma = 310$) to ensure

50% individuals are still immune after 1 year (*84*). For the re-infection rate, we choose a prior as $\gamma_s \sim$ LogNormal$(-8.660\,,\ 6.358)$. This is to ensure that average immunity period is $\frac{1}{E(\gamma_s)} \approx 8$ months, and $SD(\gamma_s) = 0.1$. This prior mean for the average immunity period is based on a recent study by Hansen et al., 2021 (*79*).

Constructing predictions for number of fatalities from the case predictions: We can also estimate the number of deaths under these forecasting scenarios under a hypothetical intervention. To do this, we apply a case fatality rate (CFR) schedule, which was calculated as the LOESS smoothed (span = 0.3) observed daily CFR (i.e., ratio of daily reported deaths to daily reported cases from 14 days prior: $\text{CFR}_t = \frac{\text{deaths}_t}{\text{cases}_{t-14}}$) for Maharashtra (high CFR), India (moderate CFR), and Kerala (low CFR) through June 30, 2021. We include the Kerala CFR schedule because of their low COVID-19 CFR relative to other large states in India and their robust healthcare system, which can be interpreted as a best-case scenario. The CFR schedules are based on observed data from February 15, 2021, through June 30, 2021, and are shown in **Figure 2E**. We then multiply the projected case counts by the CFR schedule to obtain an estimate of the daily number of COVID-19 deaths, under the presented intervention scenarios.

MCMC algorithm: Denoting t_0 as the last date of data availability, and assuming that the forecast spans over the period $[t_0 + 1, T]$, our MCMC algorithm is as follows.

0. Take M draws from the posterior $[\boldsymbol{\theta}_{\mathbf{1}:\boldsymbol{t_0}}, \boldsymbol{\tau} | \boldsymbol{Y}_{\mathbf{1}:\boldsymbol{t_0}}]$.
1. For each solution path $m \in \{1, \dots, M\}$, iterate between the following two steps via MCMC.
    i. Draw $\boldsymbol{\theta}_{\boldsymbol{t}}^{(\boldsymbol{m})}$ from $\left[\boldsymbol{\theta}_{\boldsymbol{t}} \middle| \boldsymbol{\theta}_{t-1}^{(m-1)}, \boldsymbol{\tau}^{(m)}\right], t \in \{t_0 + 1, \dots, T\}$.
    ii. Draw $\boldsymbol{Y}_{\boldsymbol{t}}^{(\boldsymbol{m})}$ from $\left[\boldsymbol{Y}_{\boldsymbol{t}} \middle| \boldsymbol{\theta}_{t}^{(m)}, \boldsymbol{\tau}^{(m)}\right], t \in \{t_0 + 1, \dots, T\}$.

We chose to present only models where $w(t) = 0$ in the main text because the results are virtually identical and the model with this setting is simpler. Results with waning immunity are presented in **Section 2.5**.

Section 2.1.2. Calculation of credible intervals

One major advantage of the Bayesian implementation is that uncertainty associated with all parameters and functions of parameters can be calculated from exact posterior draws without relying on large-scale approximation or delta theorem. The credible intervals (CI) for the prevalence and incidence are computed using the posterior distribution of proportions given the observed confirmed and removed prevalence, i.e., $Y^I_{(t_0+1):T} | Y^I_{1:t_0}, Y^R_{1:t_0}$ and $Y^R_{(t_0+1):T} | Y^I_{1:t_0}, Y^R_{1:t_0}$, where t_0 denotes the last observed date, and T denotes the last forecast date. More specifically, suppose we want to compute the 95% posterior CI for the observed proportion of confirmed cases on the first day of forecast, i.e., a CI for the random variable $Y^I_{t_0+1}$. Then, from the M solution paths of the posterior, we have the draws $\left\{Y^{I\,(m)}_{t_0+1}, 1 \le m \le M\right\}$. We construct a 95% posterior CI for $Y^I_{t_0+1}$ by simply computing the 2.5th upper and lower quantiles from this set of M draws. The cumulative prevalence is sums of the draws from the I and R compartments at a given time, whereas the incidences are the sums of successive differences in these two compartments – thus the confidence interval for these can be calculated in a similar way. Case-counts can be obtained from prevalence by using population size. Similar techniques apply to $\theta^I_{t_0+j}$ (for quantifying uncertainties for the true counterparts to the observed prevalences and incidences) for any $1 \le j \le T - t_0$ and transmission parameters like β_t, γ and γ_s.

For example, for posterior inference on β_t, we consider the decomposition $\beta_t = \beta^{(1)} \times s(t)$, where we obtain *M* posterior draws of the transmission rate of the ancestral variant, $\beta^{(1)}$. The multiplier series $s(t)$ is non-stochastic and is supplied to the model. Using the posterior draws of $\beta^{(1)}$, we can construct a posterior mean and 95% credible intervals of time-varying β_t. Finally, we smooth both the posterior estimate and the credible interval using a LOESS smoother. Similarly, we get the 95% posterior CI for the basic reproduction number R_0 from the M posterior draws $\left\{R_0^{(m)}, 1 \le m \le M\right\}$.

Estimating the cases and deaths averted due to intervention: We evaluate the intervention scenarios with different dates of introduction to illustrate the effect of timing of interventions. Let the start of the non-pharmaceutical public health intervention (NPI) be at day *t* (March 15, 30 and April 15 in our example) and the end of the follow-up period be denoted by *t*+*T* (May 15 in our case, where *T* is the number of days in the follow-up period where NPI takes place. We introduce the following notations.

$D_s = \text{Observed total reported deaths on day } s$,
$C_s = \text{Observed total reported cases on day } s$
$dcfr_s = \text{observed CFR at time } s$,
$\widehat{Y_s}$ = the predicted daily new case count on day *s* while $\widehat{P_s}$ is the predicted total case count on day *s*.

For Cases we report:

- Observed cases in period [t+1, t+T]: (1) $(C_{t+T} - C_t)$
- Predicted cases in period [t+1, t+T]: (2) $\sum_{s=t+1}^{T} \widehat{Y_s} = (\widehat{P_{t+T}} - \widehat{P_t})$
- Cases averted up to time t+T by introducing an intervention at time t:

  (3)= (1)-(2): $(C_{t+T} - C_t) - (\sum_{s=t+1}^{T} \widehat{Y_s})$

- Reduction in cases by introducing the intervention at time t:

  (Cases averted in [t+1, t+T]/Cases observed in [t+1, t+T])*100 or (4)= (3)/(1)*100

For deaths we report:

- Observed deaths in period [t+1, t+T]: (1) $(D_{t+T} - D_t)$
- Predicted deaths in period [t+1, t+T]: (2) $\sum_{s=t+1}^{T} (dcfr_s * \widehat{Y_s})$
- Deaths averted up to time *t+T* by introducing an intervention at time *t:*

  (3) = (1)-(2): $(D_{t+T} - D_t) - \sum_{s=t+1}^{T} (dcfr_s \ \widehat{Y_s})$

- Reduction in deaths by introducing the intervention at time t:

  (Deaths averted in [t+1, t+T]/Deaths observed in [t+1, t+T])*100 or (4)= (3)/(1)*100

We obtain the credible intervals from the MCMC draws of the daily number of cases predicted by the eSIR model and ignore the uncertainty in the observed CFR estimates used as weights to obtain the credible intervals for each of the above quantities which are linear combination of the posterior draws of predicted daily new cases

Section 2.2. Numerical results of intervention under low and high case fatality rate (CFR) schedules

Table S4. Classification of states and union territories in India by cumulative case fatality rate (CFR) through July 31, 2021

First tertile - low CFR				Second tertile - moderate CFR				Third tertile - high CFR			
**Place**	**Total deaths**	**Total cases**	**CFR (%)**	**Place**	**Total deaths**	**Total cases**	**CFR (%)**	**Place**	**Total deaths**	**Total cases**	**CFR (%)**
Dadra and Nagar Haveli and Daman and Diu	4	10,513	0.04	Gujarat	10,075	824,876	1.22	Jharkhand	5,128	347,147	1.48
Mizoram	148	38,064	0.39	Haryana	9,635	769,913	1.25	Pondicherry	1,795	120,917	1.48
Arunachal Pradesh	229	48,123	0.48	Karnataka	36,569	2,905,124	1.26	Manipur	1,556	98,493	1.58
Lakshadweep	50	10,189	0.49	Sikkim	344	26,562	1.30	Meghalaya	1,085	64,953	1.67
**Kerala**	**16,782**	**3,390,761**	**0.49**	Chandigarh	811	61,953	1.31	Himachal Pradesh	3,505	206,027	1.70
Telangana	3,802	644,441	0.59	Madhya Pradesh	10,514	791,828	1.33	Andaman and Nicobar Islands	129	7,533	1.71
Odisha	5,955	977,268	0.61	Bihar	9,643	724,835	1.33	Delhi	25,053	1,436,265	1.74
Andhra Pradesh	13,377	1,966,175	0.68	Tamil Nadu	34,076	2,559,597	1.33	Goa	3,147	171,146	1.84
Assam	5,265	566,096	0.93	Uttar Pradesh	22,756	1,708,441	1.33	Nagaland	560	27,835	2.01
Rajasthan	8,954	953,667	0.94	**India**	**423,793**	**31,654,522**	**1.34**	**Maharashtra**	**132,695**	**6,309,629**	**2.10**
Tripura	752	78,360	0.96	Chhattisgarh	13,524	1,002,008	1.35	Uttarakhand	7,362	342,513	2.15
Ladakh	207	20,338	1.02	Jammu and Kashmir	4,378	321,462	1.36	Punjab	16,293	599,107	2.72
West Bengal	18,136	1,528,019	1.19								

Based on total reported cases and deaths by state and union territory, and nationally, through July 31, 2021, from covid19india.org data (*16*). The cumulative case fatality rate (CFR) is calculated as the ratio of total deaths over total cases. The CFR are presented as a percentage. The rows that are bold indicate the places chosen to represent the tertile in the calculation of low, moderate, and high CFR estimates.

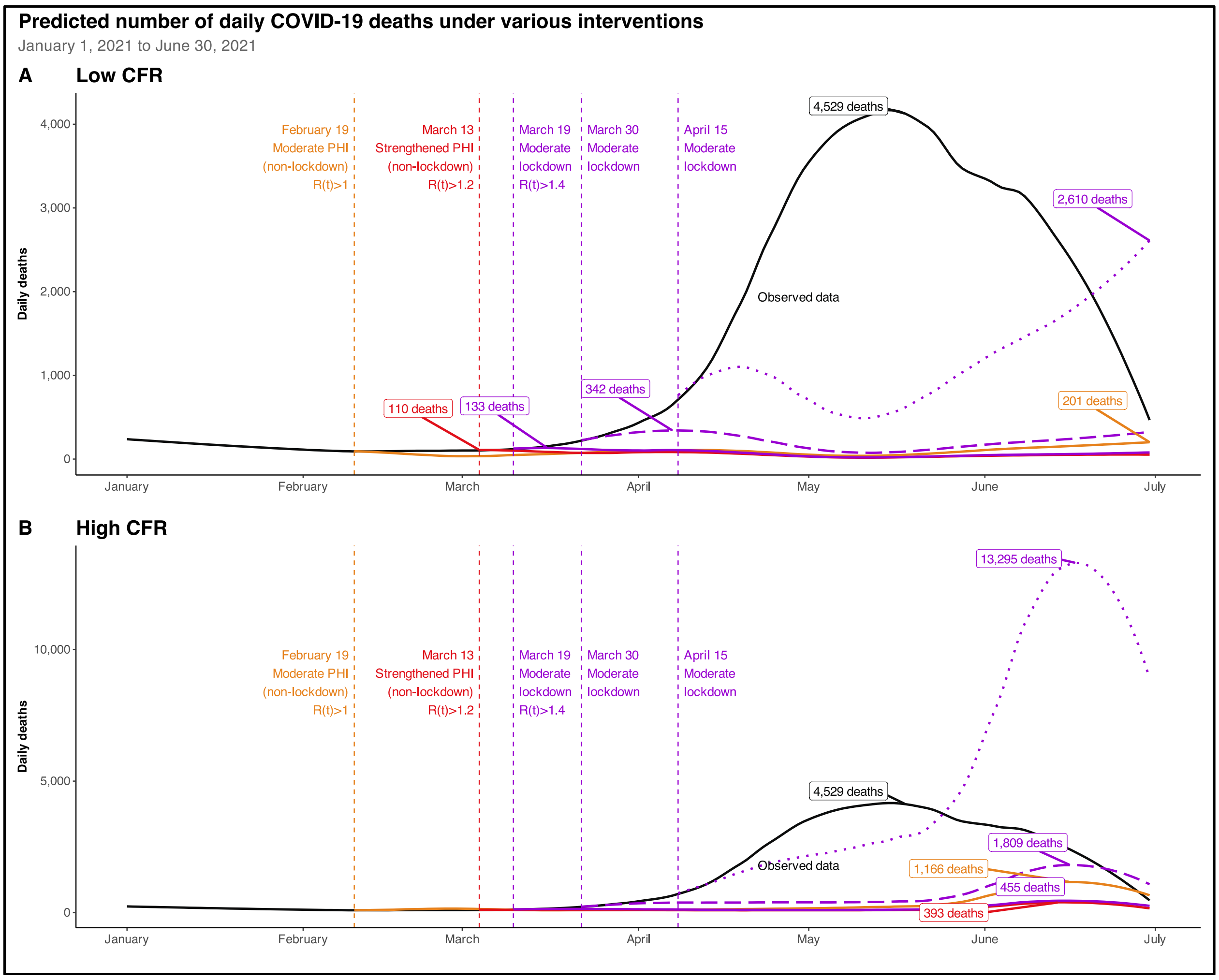

Figure S5. Observed (black) and predicted daily death counts in India, under intervention scenarios starting on different dates using low and high CFR schedules observed from February 15, 2021, to June 30, 2021. Effects of interventions are drawn from relative reductions in the time-varying effective reproduction number (R_t) in Maharashtra from April 14, 2021, to June 7, 2021 (for moderate lockdown) and March 28, 2021, to April 13, 2021 (for strengthened PHI (non-lockdown)). In addition, moderate PHI (non-lockdown) effect was estimated by reducing the effect of the strengthened PHI (non-lockdown) effect by 20%. Low and high CFR schedules are calculated from Kerala and Maharashtra, respectively. The CFR schedule is derived from the daily 14-day case lagged CFR in India (i.e., $\mathrm{CFR_t} = \frac{\mathrm{deaths_t}}{\mathrm{cases_{t-14}}}$) which was then LOESS-smoothed using span = 0.3. The daily death estimates represent the estimated daily case count multiplied by the respective CFR schedule. Three intervention start dates are depicted: moderate PHI (non-lockdown; orange) measures on February 19 (when the trailing 7-day average R_t first crossed 1), strengthened PHI (non-lockdown; red) measures on March 13 (7-day R_t > 1.2), and moderate lockdown (solid purple) measures on March 19 (7-day R_t > 1.4). Delayed moderate lockdowns on March 30 (dashed purple) and April 15 (dotted purple) are also shown. Peak daily death counts under each intervention scenario are shown.

Table S5. Predicted total death counts and deaths averted under low CFR schedule (in thousands)

Evaluation Date	Metrics	Moderate PHI (non-lockdown) start date	Strengthened PHI (non-lockdown) start date	Moderate lockdown start date		
		February 19	March 13	March 19	March 30	April 15
3/30/21	*Observed*	6.3	3.9	2.9	-	-
	Predicted	2.1 [0.0, 15.1]	1.3 [0.0, 10.9]	1.2 [0.0, 9.8]		
	Averted	4.1 [-8.8, 6.3]	2.6 [-7.1, 3.9]	1.7 [-6.9, 2.9]		
	% Reduction	66.1% [-141.5%, 100.0%]	67.4% [-183.2%, 100.0%]	59.8% [-238.2%, 100.0%]		
4/15/21	*Observed*	18.1	15.7	14.7	11.8	-
	Predicted	3.8 [0.0, 24.8]	2.6 [0.0, 18.9]	2.8 [0.0, 18.4]	5.6 [0.0, 20.9]	
	Averted	14.3 [-6.7, 18.1]	13.1 [-3.2, 15.7]	11.9 [-3.6, 14.7]	6.2 [-9.1, 11.8]	
	% Reduction	79.1% [-37.0%, 100.0%]	83.5% [-20.4%, 100.0%]	80.7% [-24.7%, 100.0%]	52.5% [-76.7%, 100.0%]	
4/30/21	*Observed*	**55.6**	**53.2**	**52.2**	**49.3**	**37.5**
	Predicted	**5.0 [0.0, 32.0]**	**3.4 [0.0, 23.1]**	**3.8 [0.0, 22.6]**	**9.0 [0.0, 30.8]**	**16.4 [0.7, 34.6]**
	Averted	**50.6 [23.6, 55.6]**	**49.8 [30.1, 53.2]**	**48.5 [29.7, 52.2]**	**40.3 [18.5, 49.3]**	**21.1 [2.9, 36.8]**
	% Reduction	**90.9% [42.4%, 100.0%]**	**93.7% [56.6%, 100.0%]**	**92.7% [56.8%, 100.0%]**	**81.8% [37.6%, 100.0%]**	**56.3% [7.9%, 98.1%]**
5/15/21	*Observed*	**114.1**	**111.7**	**110.7**	**107.8**	**96.0**
	Predicted	**5.7 [0.0, 35.4]**	**3.7 [0.0, 24.3]**	**4.1 [0.0, 24.0]**	**10.3 [0.0, 34.0]**	**24.3 [4.0, 48.7]**
	Averted	**108.4 [78.7, 114.1]**	**108.0 [87.4, 111.7]**	**106.6 [86.7, 110.7]**	**97.5 [73.8, 107.8]**	**71.6 [47.3, 92.0]**
	% Reduction	**95.0% [69.0%, 100.0%]**	**96.7% [78.2%, 100.0%]**	**96.3% [78.3%, 100.0%]**	**90.4% [68.5%, 100.0%]**	**74.6% [49.3%, 95.8%]**
5/30/21	*Observed*	**172.9**	**170.5**	**169.5**	**166.6**	**154.8**
	Predicted	**6.7 [0.0, 42.1]**	**4.1 [0.0, 26.4]**	**4.6 [0.0, 26.1]**	**12.1 [0.0, 39.4]**	**36.7 [8.2, 72.5]**
	Averted	**166.2 [130.8, 172.9]**	**166.4 [144.1, 170.5]**	**164.9 [143.5, 169.5]**	**154.5 [127.2, 166.6]**	**118.1 [82.3, 146.6]**
	% Reduction	**96.1% [75.6%, 100.0%]**	**97.6% [84.5%, 100.0%]**	**97.3% [84.6%, 100.0%]**	**92.7% [76.4%, 100.0%]**	**76.3% [53.2%, 94.7%]**
6/15/21	*Observed*	**223.4**	**221.0**	**220.0**	**217.1**	**205.3**
	Predicted	**8.8 [0.0, 56.9]**	**4.8 [0.0, 31.3]**	**5.5 [0.0, 30.9]**	**15.3 [0.0, 51.0]**	**59.9 [14.5, 126.5]**
	Averted	**214.5 [166.5, 223.4]**	**216.1 [189.6, 221.0]**	**214.5 [189.1, 220.0]**	**201.8 [166.1, 217.1]**	**145.4 [78.7, 190.7]**
	% Reduction	**96.1% [74.5%, 100.0%]**	**97.8% [85.8%, 100.0%]**	**97.5% [85.9%, 100.0%]**	**92.9% [76.5%, 100.0%]**	**70.8% [38.4%, 92.9%]**

Notes: Each cell reports (1) the total number of observed deaths since the start of intervention in the first row, (2) total number of predicted deaths since the start of intervention in the second row (with 95% CI), (3) the number of deaths averted (relative to observed) since the start of intervention in the third row (with 95% CI), and (4) the relative reduction in cases (as a percent) under lockdown in the fourth row (with 95% CI) from the intervention start date through the evaluation date. Cells that are bolded represent a statistically significant reduction in the number of cases under intervention at the 95% credible interval level. Numbers are reported in thousands.

Table S6. Predicted total death counts and deaths averted under high CFR schedule (in thousands)

Evaluation Date	Metrics	Moderate PHI (non-lockdown) start date	Strengthened PHI (non-lockdown) start date	Moderate lockdown start date		
		February 19	March 13	March 19	March 30	April 15
3/30/21	*Observed*	6.3	3.9	2.9	-	-
	Predicted	4.9 [0.0, 36.0]	1.7 [0.0, 14.7]	1.5 [0.0, 13.0]		
	Averted	1.3 [-29.7, 6.3]	2.2 [-10.8, 3.9]	1.4 [-10.1, 2.9]		
	% Reduction	21.3% [-475.4%, 100.0%]	56.0% [-280.8%, 100.0%]	47.0% [-346.0%, 100.0%]		
4/15/21	*Observed*	18.1	15.7	14.7	11.8	-
	Predicted	6.8 [0.0, 44.4]	3.2 [0.0, 23.1]	3.5 [0.0, 22.3]	6.5 [0.0, 24.2]	
	Averted	11.3 [-26.3, 18.1]	12.5 [-7.4, 15.7]	11.3 [-7.6, 14.7]	5.3 [-12.4, 11.8]	
	% Reduction	62.2% [-145.7%, 100.0%]	79.5% [-47.1%, 100.0%]	76.4% [-51.4%, 100.0%]	45.0% [-104.8%, 100.0%]	
4/30/21	*Observed*	55.6	**53.2**	**52.2**	**49.3**	37.5
	Predicted	9.0 [0.0, 55.9]	**4.6 [0.0, 32.1]**	**5.1 [0.0, 31.3]**	**12.4 [0.0, 42.9]**	28.7 [2.1, 60.4]
	Averted	46.6 [-0.3, 55.6]	**48.6 [21.1, 53.2]**	**47.1 [21.0, 52.2]**	**36.9 [6.4, 49.3]**	8.8 [-22.9, 35.4]
	% Reduction	83.7% [-0.5%, 100.0%]	**91.4% [39.6%, 100.0%]**	**90.2% [40.1%, 100.0%]**	**74.9% [13.0%, 100.0%]**	23.5% [-61.1%, 94.4%]
5/15/21	*Observed*	**114.1**	**111.7**	**110.7**	**107.8**	96.0
	Predicted	**12.0 [0.0, 73.9]**	**6.0 [0.0, 41.7]**	**6.6 [0.0, 41.1]**	**18.4 [0.0, 62.6]**	65.2 [12.5, 130.9]
	Averted	**102.1 [40.2, 114.1]**	**105.7 [70.0, 111.7]**	**104.1 [69.6, 110.7]**	**89.5 [45.2, 107.8]**	30.8 [-34.9, 83.5]
	% Reduction	**89.5% [35.2%, 100.0%]**	**94.7% [62.7%, 100.0%]**	**94.0% [62.8%, 100.0%]**	**83.0% [41.9%, 100.0%]**	32.0% [-36.4%, 87.0%]
5/30/21	*Observed*	**172.9**	**170.5**	**169.5**	**166.6**	154.8
	Predicted	**16.5 [0.0, 103.9]**	**7.9 [0.0, 57.0]**	**8.8 [0.0, 55.7]**	**26.2 [0.0, 91.0]**	119.0 [27.7, 243.5]
	Averted	**156.4 [69.0, 172.9]**	**162.6 [113.5, 170.5]**	**160.8 [113.8, 169.5]**	**140.5 [75.6, 166.6]**	35.8 [-88.7, 127.1]
	% Reduction	**90.5% [39.9%, 100.0%]**	**95.4% [66.6%, 100.0%]**	**94.8% [67.1%, 100.0%]**	**84.3% [45.4%, 100.0%]**	23.1% [-57.3%, 82.1%]
6/15/21	*Observed*	223.4	**221.0**	**220.0**	**217.1**	205.3
	Predicted	31.7 [0.0, 223.9]	**13.0 [0.0, 116.9]**	**15.0 [0.0, 119.0]**	**49.4 [0.0, 202.7]**	286.3 [48.3, 668.1]
	Averted	191.7 [-0.6, 223.4]	**207.9 [104.0, 221.0]**	**205.0 [101.0, 220.0]**	**167.7 [14.4, 217.1]**	-81.1 [-462.8, 157.0]
	% Reduction	85.8% [-0.3%, 100.0%]	**94.1% [47.1%, 100.0%]**	**93.2% [45.9%, 100.0%]**	**77.3% [6.6%, 100.0%]**	-39.5% [-225.5%, 76.5%]

Notes: Each cell reports (1) the total number of observed deaths since the start of intervention in the first row, (2) total number of predicted deaths since the start of intervention in the second row (with 95% CI), (3) the number of deaths averted (relative to observed) since the start of intervention in the third row (with 95% CI), and (4) the relative reduction in cases (as a percent) under lockdown in the fourth row (with 95% CI) from the intervention start date through the evaluation date. Cells that are bolded represent a statistically significant reduction in the number of cases under intervention at the 95% credible interval level. Numbers are reported in thousands.

Section 2.3. Numerical results from strong lockdown intervention

Here we present the numerical results of a strong lockdown intervention had it taken place on March 13, March 19, March 30, or April 15, 2021. By strong lockdown, we mean a lockdown that would have the same effect (on time-varying R_t) as the one seen during the nationwide lockdown instituted during the first wave of the COVID-19 pandemic in India in March 2020. The intervention schedule and description of its derivation are presented in **Supplementary Section 2.2**.

The results for reduction in cases under strong lockdown (**Table S7**) on various start dates is effective even as late as April 15, 2021. However, there is a significant drop off in the relative reduction in cases through June 15, 2021 (56.0% [95% CI: 10.7%, 92.0%] with an April 15, 2021, start date vs 91.0% [95% CI 66.6%, 100.0%] with a March 30, 2021, start date).

Table S7. Predicted total case counts, cases averted and % reduction with corresponding 95% credible intervals under strong lockdown intervention (in millions)

Evaluation Date	Metrics	Strong lockdown start date: March 13	March 19	March 30	April 15
3/30/21	*Observed*	0.8	0.6	-	-
	Predicted	0.2 [0.0, 1.8]	0.2 [0.0, 1.5]		
	Averted	0.6 [-1.0, 0.8]	0.4 [-0.9, 0.6]		
	% Reduction	78.6% [-127.5%, 100.0%]	72.6% [-158.2%, 100.0%]		
4/15/21	*Observed*	**2.9**	**2.7**	2.1	-
	Predicted	**0.3 [0.0, 2.5]**	**0.3 [0.0, 2.4]**	0.6 [0.0, 2.5]	
	Averted	**2.7 [0.4, 2.9]**	**2.4 [0.3, 2.7]**	1.5 [-0.3, 2.1]	
	% Reduction	**91.0% [13.7%, 100.0%]**	**88.5% [12.2%, 100.0%]**	71.7% [-15.2%, 100.0%]	
4/30/21	*Observed*	**7.8**	**7.6**	**7.0**	**4.9**
	Predicted	**0.3 [0.0, 3.0]**	**0.4 [0.0, 3.0]**	**1.0 [0.0, 3.6]**	**2.2 [0.0, 4.8]**
	Averted	**7.5 [4.8, 7.8]**	**7.2 [4.6, 7.6]**	**6.0 [3.4, 7.0]**	**2.6 [0.1, 4.8]**
	% Reduction	**95.9% [60.9%, 100.0%]**	**94.6% [60.7%, 100.0%]**	**86.1% [48.7%, 100.0%]**	**53.8% [1.3%, 99.4%]**
5/15/21	*Observed*	**13.3**	**13.1**	**12.5**	**10.4**
	Predicted	**0.4 [0.0, 3.5]**	**0.5 [0.0, 3.5]**	**1.2 [0.0, 4.5]**	**4.1 [0.5, 8.2]**
	Averted	**13.0 [9.9, 13.3]**	**12.6 [9.6, 13.1]**	**11.3 [8.1, 12.5]**	**6.3 [2.2, 9.9]**
	% Reduction	**97.3% [74.0%, 100.0%]**	**96.4% [73.0%, 100.0%]**	**90.2% [64.3%, 100.0%]**	**60.7% [21.0%, 95.0%]**
5/30/21	*Observed*	**16.7**	**16.5**	**15.9**	**13.8**
	Predicted	**0.4 [0.0, 3.8]**	**0.5 [0.0, 4.0]**	**1.4 [0.0, 5.2]**	**5.5 [0.9, 10.9]**
	Averted	**16.3 [12.9, 16.7]**	**16.0 [12.5, 16.5]**	**14.5 [10.7, 15.9]**	**8.3 [2.9, 12.8]**
	% Reduction	**97.7% [77.0%, 100.0%]**	**96.8% [75.9%, 100.0%]**	**91.1% [67.3%, 100.0%]**	**60.0% [20.8%, 93.1%]**
6/15/21	*Observed*	**18.3**	**18.1**	**17.5**	**15.3**
	Predicted	**0.4 [0.0, 4.1]**	**0.6 [0.0, 4.4]**	**1.6 [0.0, 5.8]**	**6.8 [1.2, 13.7]**
	Averted	**17.9 [14.1, 18.3]**	**17.5 [13.7, 18.1]**	**15.9 [11.6, 17.5]**	**8.6 [1.6, 14.1]**
	% Reduction	**97.8% [77.4%, 100.0%]**	**96.9% [75.7%, 100.0%]**	**91.0% [66.6%, 100.0%]**	**56.0% [10.7%, 92.0%]**

Notes: Each cell reports (1) the total number of observed cases since the start of lockdown in the first row, (2) total number of predicted cases since the start of lockdown in the second row (with 95% CI), (3) the number of cases averted (relative to observed) since the start of lockdown in the third row (with 95% CI), and (4) the relative reduction in cases (as a percent) under lockdown in the fourth row (with 95% CI) from the intervention start date through the evaluation date. Cells that are bolded represent a statistically significant reduction in the number of cases under intervention at the 95% credible interval level. Numbers are reported in millions.

There are similar results for reduction in deaths under strong lockdown (**Table S8**) on various start dates. Again, there is a significant drop off in the relative reduction in deaths through June 15, 2021 (54.9% [95% CI: 10.0%, 91.5%] with an April 15, 2021, start date vs 90.1% [95% CI 63.0%, 100.0%] with a March 30, 2021, start date).

Table S8. Predicted total death counts, deaths averted and % reduction with corresponding 95% credible intervals under strong lockdown and moderate CFR (in thousands)

Evaluation		Strong lockdown start date			
Date	Metrics	March 13	March 19	March 30	April 15
3/30/21	*Observed*	3.9	2.9	-	-
	Predicted	1.8 [0.0, 19.4]	1.9 [0.0, 17.4]		
	Averted	2.0 [-15.6, 3.9]	1.1 [-14.5, 2.9]		
	% Reduction	52.8% [-403.5%, 100.0%]	36.4% [-499.4%, 100.0%]		
4/15/21	*Observed*	15.7	14.7	11.8	-
	Predicted	3.1 [0.0, 30.7]	3.9 [0.0, 30.5]	8.2 [0.0, 33.7]	
	Averted	12.6 [-15.0, 15.7]	10.8 [-15.7, 14.7]	3.6 [-21.9, 11.8]	
	% Reduction	80.2% [-95.5%, 100.0%]	73.4% [-106.8%, 100.0%]	30.6% [-184.9%, 100.0%]	
4/30/21	*Observed*	**53.2**	**52.2**	49.3	37.5
	Predicted	**4.0 [0.0, 41.4]**	**5.5 [0.0, 41.7]**	14.2 [0.0, 53.3]	36.7 [0.5, 78.4]
	Averted	**49.2 [11.8, 53.2]**	**46.8 [10.6, 52.2]**	35.1 [-4.0, 49.3]	0.8 [-40.9, 37.0]
	% Reduction	**92.5% [22.2%, 100.0%]**	**89.5% [20.3%, 100.0%]**	71.1% [-8.0%, 100.0%]	2.2% [-109.0%, 98.6%]
5/15/21	*Observed*	**111.7**	**110.7**	**107.8**	96.0
	Predicted	**4.5 [0.0, 45.9]**	**6.3 [0.0, 47.9]**	**17.4 [0.0, 63.6]**	59.5 [7.5, 119.4]
	Averted	**107.2 [65.8, 111.7]**	**104.4 [62.8, 110.7]**	**90.4 [44.2, 107.8]**	36.4 [-23.4, 88.5]
	% Reduction	**96.0% [58.9%, 100.0%]**	**94.3% [56.7%, 100.0%]**	**83.9% [41.0%, 100.0%]**	38.0% [-24.4%, 92.2%]
5/30/21	*Observed*	**170.5**	**169.5**	**166.6**	**154.8**
	Predicted	**4.7 [0.0, 49.0]**	**6.8 [0.0, 51.8]**	**19.4 [0.0, 70.1]**	**74.7 [12.9, 146.6]**
	Averted	**165.8 [121.5, 170.5]**	**162.7 [117.7, 169.5]**	**147.2 [96.5, 166.6]**	**80.1 [8.2, 141.9]**
	% Reduction	**97.2% [71.2%, 100.0%]**	**96.0% [69.4%, 100.0%]**	**88.4% [57.9%, 100.0%]**	**51.8% [5.3%, 91.7%]**
6/15/21	*Observed*	**221.0**	**220.0**	**217.1**	**205.3**
	Predicted	**5.0 [0.0, 54.6]**	**7.4 [0.0, 58.4]**	**21.6 [0.0, 80.3]**	**92.5 [17.5, 184.7]**
	Averted	**215.9 [166.3, 221.0]**	**212.6 [161.6, 220.0]**	**195.5 [136.8, 217.1]**	**112.8 [20.6, 187.8]**
	% Reduction	**97.7% [75.3%, 100.0%]**	**96.6% [73.4%, 100.0%]**	**90.1% [63.0%, 100.0%]**	**54.9% [10.0%, 91.5%]**

Notes: Each cell reports (1) the total number of observed deaths since the start of intervention in the first row, (2) total number of predicted deaths since the start of intervention in the second row (with 95% CI), (3) the number of deaths averted (relative to observed) since the start of intervention in the third row (with 95% CI), and (4) the relative reduction in cases (as a percent) under lockdown in the fourth row (with 95% CI) from the intervention start date through the evaluation date. Cells that are bolded represent a statistically significant reduction in the number of cases under intervention at the 95% credible interval level. Numbers are reported in thousands.

Section 2.4. Presentation of results with waning immunity

This section shows the data for the projections that allow for waning immunity (**Figure S6**). In this projection, we modeled time-dependent waning immunity as a Rayleigh distribution to ensure 50% of individuals are still immune after 1 year, as described in the **Supplementary Section 2.2.1**. The plots demonstrate virtually indistinguishable results from the projections without waning immunity (**Figure 3**).

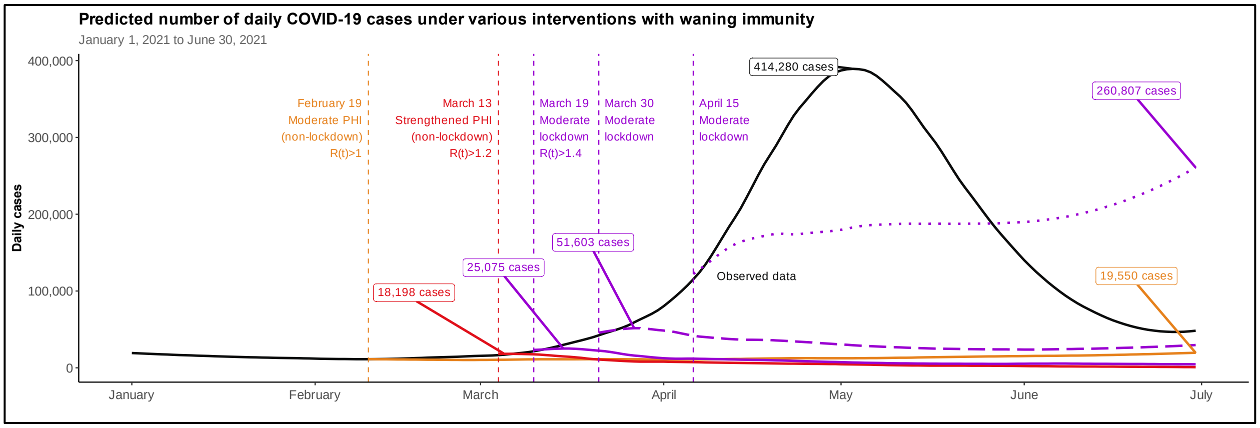

Figure S6. Observed (black) and predicted daily case counts from February 15, 2021, to June 30, 2021, in India, under intervention scenarios with waning immunity starting on different dates. Predictions under moderate PHI (non-lockdown; orange), strengthened PHI (non-lockdown; red), and moderate lockdown (purple) intervention effect schedules are described. Moderate PHI (non-lockdown) and strengthened PHI (non-lockdown) measures curbed transmission in a manner than avoided escalation to a lockdown; however, these interventions were continued throughout the entire prediction period. Effect of interventions are drawn from relative reduction in time-varying effective reproduction number (R_t) from Maharashtra after the implementation of their COVID-19 interventions in March 2021 and April 2021. The intervention effect schedules are then smoothed using a LOESS smoother (span = 1) to account for day-to-day variations in R_t. Three intervention start dates are depicted: moderate PHI (non-lockdown; orange) measures on February 19, 2021, (when the trailing 7-day average R_t first crossed 1), strengthened PHI (non-lockdown; red) measures on March 13, 2021 (7-day R_t > 1.2) and moderate lockdown (solid purple) measures on March 19, 2021 (7-day R_t > 1.4). Delayed moderate lockdowns on March 30, 2021 (dashed purple) and April 15, 2021 (dotted purple) are also shown. Peak daily case counts under each intervention scenario are shown.

Section 2.5. Effect of unlocking and extending the length of lockdowns

We also conducted a series of analyses to understand the impact of unlocking (i.e., relaxing lockdown restriction) after two months of lockdowns initiated at different time point in the second wave (**Figure S7**). We derived the impact of unlocking from the effect observed during the unlock period in Maharashtra (from June 7, 2021, to July 31, 2021). While unlocking is associated with a modest uptick in COVID-19 cases when moderate lockdowns are initiated relatively early in the second wave (e.g., March 19, 2021, or March 30, 2021), unlocking is associated with a more dramatic increase when a moderate lockdown is initiated at a more delayed time point (e.g., April 15, 2021). These pandemic control benefits during the unlocking period provide another rationale for escalating PHI early in a COVID-19 wave.

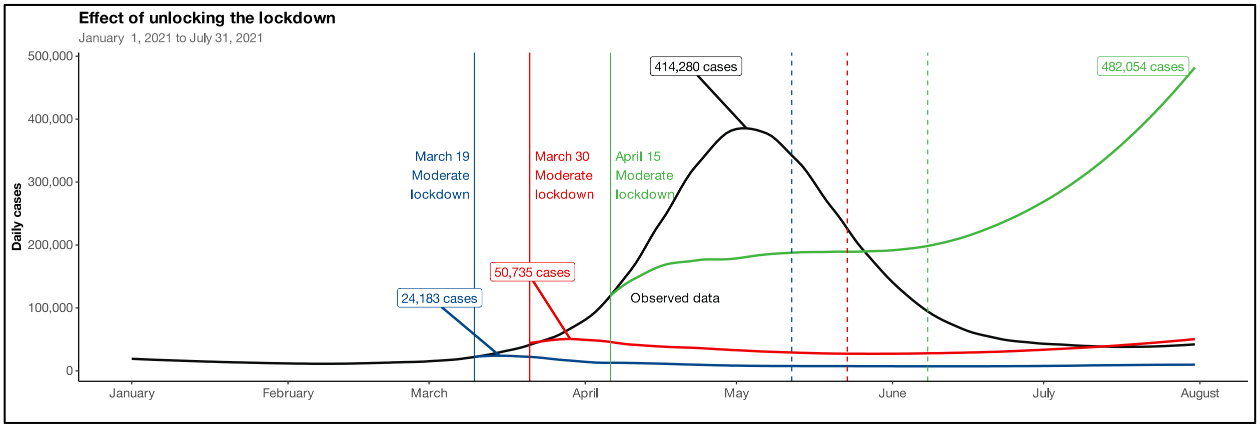

Figure S7. Effect of moderate lockdown followed by unlocking (i.e., relaxation of lockdown) COVID-19 cases in India. Each lockdown scenario is continued for two months, after which unlocking is initiated. Projections in the unlocking period are estimated from the effect observed during the unlocking period in Maharashtra from June 7, 2021, to July 31, 2021. Moderate lockdowns initiated on March 19, 2021 (solid blue), March 24, 2021 (solid red), or April 15, 2021 (solid green) are associated with unlocking dates of May 12, 2021 (dashed blue), May 29, 2021 (dashed red) and June 8, 2021 (dashed green), respectively. Peak daily case counts under each intervention scenario are shown.

We then conducted an analysis to understand the impact of varying the length of lockdowns on trends in subsequent COVID-19 cases during the unlocking period (**Figure S7**). We compare 4-, 6-, and 8-week versions of the moderate lockdown scenario on three different start dates: March 19 (**Figure S8A**), March 30 (**Figure S8B**), and April 15 (**Figure S8C**).

We see that if instituted on March 19, 2021, moderate lockdowns for 4-, 6-, and 8-weeks result in an early peak of similar magnitude with sustained benefit in case reduction over the prediction period (**Figure S8A**). If delayed to March 30, 2021, 6- and 8- week moderate lockdowns result in early peaks and sustained reduction of cases, while a 4-week lockdown does not have a sustained benefit with a peak occurring at the end of the prediction period (indicating an increasing trend in case counts; **Figure S8B**). However, if a moderate lockdown is instituted on April 15, 2021, extending the length of the lockdown (e.g., from 4 weeks to either 6 weeks or 8 weeks) has only modest incremental benefit, as COVID-19 cases still rise rapidly after unlocking in all these scenarios (**Figure S8C**). The speed of the rise (i.e., slope) in cases in the unlocking period drops appreciably from a 4-week lockdown to a 6-week lockdown with an additional reduction with an 8-week lockdown. While a moderate lockdown starting on April 15, 2021, may have been too late to avoid a surge in cases post-lockdown, this analysis shows an extended lockdown would delay and potentially reduce the size of that surge.

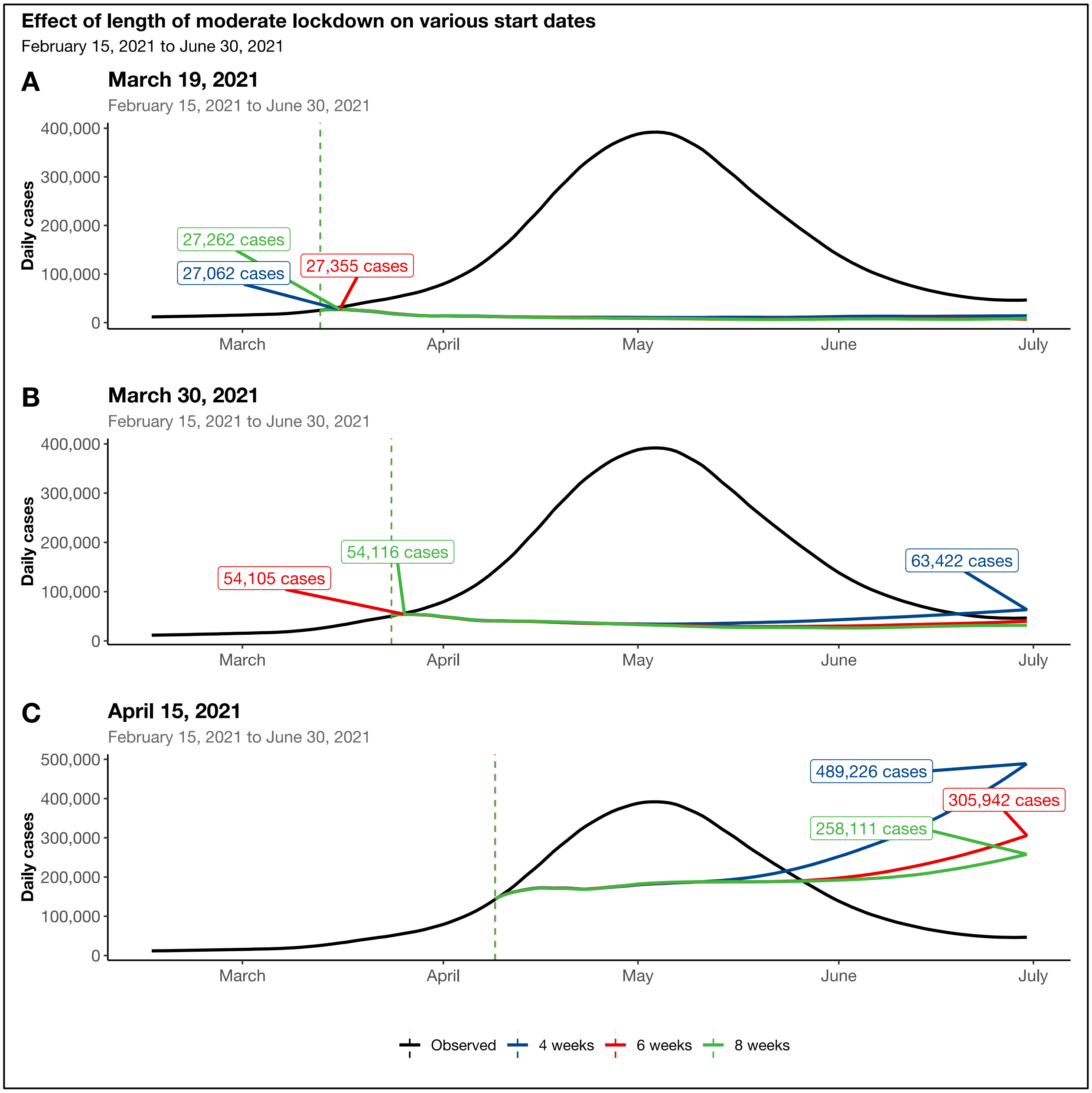

Figure S8. Comparing the effect of moderate lockdown on COVID-19 cases in India had it been instituted nationwide on various dates with varying durations of implementation. The time of "unlocking" (i.e., relaxation of lockdown restrictions) is varied to start after 4 weeks of lockdown (blue), 6 weeks of lockdown (red), and 8 weeks of lockdown (green). Locking and unlocking effects are adapted from Maharashtra in 2021 (moderate lockdown scenario). Peak daily case counts under each scenario are shown.

Section 3. Main text scenarios using a modified SEIR model

To assess the sensitivity of the results to model choices we used another alternative modeling approach to the eSAIR model. This modified version of the common Susceptible-Exposed-Infected-Recovered (SEIR) infectious disease compartmental model was modified to account for high false-negative test results and symptom-based administration of testing, called SEIRfansy. The SEIRfansy model was developed by Bhaduri et al. 2022 (*42*) to study the COVID-19 outbreak in India and can be implemented via the R package SEIRfansy (*80*). Because we are assuming no false-negative testing in our scenarios (i.e., $f = 0$), we presented a simplified schematic (hereafter simply SEIR) without the tested and false-negative compartments. The SEIR model diagram, its corresponding set of differential equations, and the model parameters and their choices and descriptions are presented in **Figure S9**.

Description of compartmental model and underlying transmission dynamics

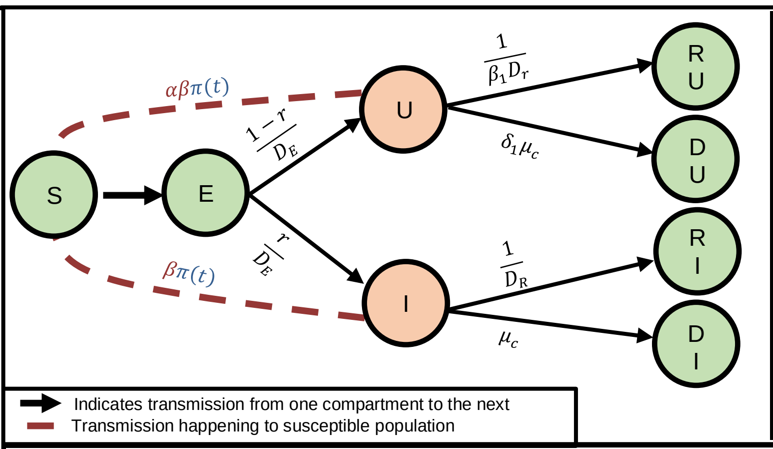

System of differential equations

$$\frac{\delta S}{\delta t} = -\frac{\beta\pi(t)S(t)}{N}\big(P(t)+\alpha U(t)\big) + \lambda N - \mu S(t)$$
$$\frac{\delta E}{\delta t} = \frac{\beta\pi(t)S(t)}{N}\big(P(t)+\alpha U(t)\big) - \frac{E(t)}{D_e} - \mu E(t)$$
$$\frac{\delta U}{\delta t} = \frac{(1-r)E(t)}{D_e} - \frac{U(t)}{\beta_1 D_r} - \delta_1\mu_c U(t) - \mu U(t)$$
$$\frac{\delta P}{\delta t} = \frac{rE(t)}{D_e} - \frac{P(t)}{D_r} - \mu_c P(t) - \mu P(t)$$
$$\frac{\delta RU}{\delta t} = \frac{U(t)}{\beta_1 D_r} - \mu RU(t)$$
$$\frac{\delta RR}{\delta t} = \frac{P(t)}{D_r} - \mu RR(t)$$
$$\frac{\delta DU}{\delta t} = \delta_1\mu_c U(t)$$
$$\frac{\delta DR}{\delta t} = \mu_c P(t)$$

We consider the Susceptible-Exposed-Infected-Recovered (SEIR) model that considers both detected and undetected infections and deaths. A feature of this model is that it can give estimates of underreporting factors. In our formulation the model consists of eight compartments, and we are estimating two time-varying parameters *β (transmission rate)* and *r (ascertainment rate)* under a hierarchical Bayes model specification and governed by the above system of equations. The training period is divided into an initial 25-day bin and then subsequent 15-day bins with each time interval allowing for a different *β* and *r to* account for time varying transmission and ascertainment rates. Here *r* is the probability of being tested/ascertained for an infectious individual which is set at a starting value of 0.05, that is equivalent to an underreporting factor of 20, consistent with national seroprevalence surveys in January 2021 (*17*). For the two time-varying parameters we are assuming flat non-informative priors and the differential equations are solved recursively by discretizing the equations. We allow for time-varying intervention effects on the transmission rate through $\pi(t)$ during the prediction period. The table below describes the parameters and their choices in greater detail.

Parameters and their descriptions

$\beta; \pi(\beta_t) \propto \mathbb{I}(\beta_t > 0)$	Rate of transmission of infection by positive individuals
$r \sim U(0,\ 1)$	Probability of being tested for infection
$\alpha = 1.0$	Scaling factor for the rate of transmission by untested individuals. May be assumed to be less than 1 as U compartment mostly consists of asymptomatic or mildly symptomatic cases who are less likely to be contagious than those having symptoms
$D_E = 5.2$ days	Incubation period (in number of days) (*95*)
$D_r = 17.8$ days	Mean number of days till recovery for those who test positive (*96*)
$\text{mCFR} = \frac{\text{Cumulative deaths}}{\text{Cumulative deaths} + \text{Cumulative recovered}}$	Case-fatality rate estimated to be the ratio of (reported) cumulative deaths over (reported) cumulative deaths and recovered at the end of the training period
$\mu_c = \frac{\text{mCFR}}{17.8}$	Death rate due to COVID-19 infection which is equivalent to the inverse of the average number of days from disease onset to death times the true infection fatality rate
$\lambda = \mu = 3.95 \times 10^{-5}$	Natural birth and death rates in the population. (*97*) Assumed to be equal for the sake of simplicity.
$\beta_1 = 0.6$	Scaling factors for rate of recovery for untested infectious individuals. The severity of symptoms in untested individuals is assumed to be less than those tested positive. Consequently, untested individuals are assumed to recover faster than those who tested positive.
$\delta_1 = 0.3$	Scaling factors for death rate for untested infectious individuals, respectively. The untested individuals are assumed to have a smaller probability of dying relative to those who test positive, since untested people are mostly asymptomatic.

Figure S9. A schematic representation of the compartments of the SEIR model. A graphical depiction of the compartments of the model (upper left) shows the flow between compartments and the key rates describing these transitions, which are accompanied by the system of differential equations (upper right). The lower half of the figure lists the key model parameters, their descriptions, and the rationale for their values. Adaptation of Figure 1 from Bhaduri et al. 2022 (*42*) (published in *Statistics in Medicine* by John Wiley & Sons Ltd. under Creative Commons license CC BY-NC 4.0). In the figure U and I correspond to Undetected latent Infectious and Identified Infectious people, respectively.

To address concerns regarding underlying seropositivity in India, we have implemented the SEIR model. The SEIR model allows for time-varying β and ascertainment rate *r* over different periods (an initial 25-day bin and subsequent 15-day bins into which the 100-day training period is divided; hereafter simply 15-day bins for simplicity). Here we use a Bayesian approach for estimating the parameters, which is based on an underlying compartmental model framework. In this Bayesian approach, we specify the likelihood and the prior distributions. To incorporate time varying parameters, we have divided the product of likelihoods, into the 15-day bins. The time varying parameters β and *r* changes in the likelihood over the 15-day bins and these parameters are subsequently estimated by MCMC Metropolis Hastings Algorithm which uses the above likelihood and non-informative flat priors. The β estimated from the last period (in this case, the last 15-day bin of the training period) is multiplied by the $\pi(t)$ schedule to get modified predictions in the same manner as we get for the eSIR model in the main text does (**Materials and Methods** in manuscript).

Using the same training data, intervention effect schedules (**Figure 2D**), and intervention start dates, we use the SEIR model to predict reported cases (**Figure S10**) and reported deaths (**Figure S11**).

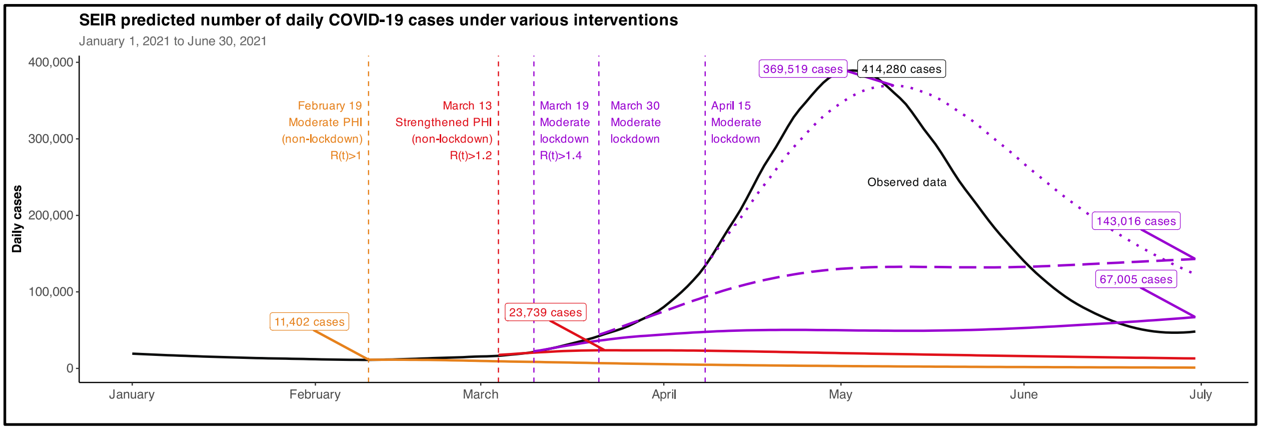

Figure S10. Observed (black) and SEIR-predicted reported daily case counts from February 15 to June 30, 2021, in India, under intervention scenarios starting on different dates. Predictions under moderate PHI (non-lockdown; orange), strengthened PHI (non-lockdown; red), and moderate lockdown (purple) intervention effect schedules are described. Moderate and strengthened PHI do not contain a lockdown but continue throughout the entire prediction period. Effect of interventions are drawn from relative reduction in time-varying effective reproduction number (R_t) from Maharashtra after the implementation of their COVID-19 interventions in March and April 2021. The intervention effect schedules are then smoothed using a LOESS smoother to account for day-to-day variations in R_t. Three intervention start dates are depicted: moderate PHI (non-lockdown; orange) measures on February 19 (when the trailing 7-day average R_t first crossed 1), strengthened PHI (non-lockdown; red) measures on March 13 (7-day R_t > 1.2) and moderate lockdown (solid purple) measures on March 19 (7-day R_t > 1.4). Delayed moderate lockdowns on March 30 (dashed purple) and April 15 (dotted purple) are also shown. Peak daily case counts under each intervention scenario are shown.

From **Figure S10**, we see that moderate PHI (non-lockdown) on February 19, strengthened PHI (non-lockdown) on March 13, and moderate lockdown on March 19 appear effective at avoiding a surge. Moderate lockdown on March 19 avoids a surge, but case counts appear elevated throughout the prediction period. In these three

scenarios, the SEIR model estimates that the reduction in cases ranges from 78-97% through June 15, 2021 (depending on the intervention and start date). This reduction in cases drops significantly to around 50% for a moderate lockdown starting on March 30. It's evident that by April 15, a moderate lockdown is too late, with the SEIR model predicting approximately 20% more cases than what was observed. The qualitative conclusions from this model are like those presented in the main text (**Figure 3**) – specifically, that timely non-lockdown interventions are effective at avoiding a second wave and the need for a lockdown. Additionally, March seems to be the effective time period for a moderate lockdown. The numeric results are presented in **Table S9**. We believe a limitation is that the reported confidence levels are very narrow and do not reflect a reasonable level of uncertainty.

Table S9. SEIR-predicted total case counts, cases averted and % reduction with corresponding 95% credible intervals under different lockdown interventions (in millions)

Evaluation Date	Metrics	Moderate PHI (non-lockdown) start date	Strengthened PHI (non-lockdown) start date	Moderate lockdown start date		
		February 19	March 13	March 19	March 30	April 15
3/30/21	*Observed*	**1.2**	**0.8**	**0.6**	**-**	**-**
	Predicted	**0.32 [0.31, 0.32]**	**0.41 [0.4, 0.41]**	**0.46 [0.45, 0.47]**		
	Averted	**0.88 [0.88, 0.89]**	**0.39 [0.39, 0.4]**	**0.14 [0.13, 0.15]**		
	% Reduction	**73.6% [72%, 74.2%]**	**48.8% [48.3%, 49.4%]**	**23.4% [22.3%, 24.3%]**		
4/15/21	*Observed*	**3.3**	**2.9**	**2.7**	**2.1**	**-**
	Predicted	**0.39 [0.38, 0.4]**	**0.77 [0.76, 0.78]**	**1.21 [1.19, 1.23]**	**1.54 [1.53, 1.56]**	
	Averted	**2.91[2.9, 2.92]**	**2.13 [2.12, 2.14]**	**1.49 [1.47, 1.51]**	**0.56 [0.54 0.57]**	
	% Reduction	**88.2% [87.9%, 88.6%]**	**73.4% [73.04%, 73.8%]**	**55.2% [54.3%, 55.9%]**	**26.5% [25.8%, 27.2%]**	
4/30/21	*Observed*	**8.2**	**7.8**	**7.6**	**7.0**	**4.9**
	Predicted	**0.44[0.42, 0.45]**	**1.08[1.06, 1.1]**	**1.96[1.91, 2]**	**3.36[3.32, 3.4]**	**4.5 [4.45, 4.58]**
	Averted	**7.76[7.75, 7.78]**	**6.72[6.7, 6.74]**	**5.64[5.6, 5.68]**	**3.63[3.6, 3.68]**	**0.4 [0.32, 0.45]**
	% Reduction	**94.7% [94.5%, 94.9%]**	**86.1% [85.9%, 86.3%]**	**74.3% [73.7%, 74.8%]**	**51.9% [51.4%, 52.5%]**	**8.3% [6.6%, 9.3%]**
5/15/21	*Observed*	**13.7**	**13.3**	**13.1**	**12.5**	**10.4**
	Predicted	**0.47 [0.45, 0.49]**	**1.36[1.34, 1.38]**	**2.69[2.63, 2.7]**	**5.34[5.27, 5.4]**	**9.9 [9.8, 10.3]**
	Averted	**13.23 [13.21, 13.27]**	**11.94[11.92, 11.96]**	**10.4[10.34, 10.5]**	**7.16[7.1, 7.23]**	**0.45 [0.11, 0.64]**
	% Reduction	**96.6% [96.4%, 96.7%]**	**89.8% [89.6%, 90%]**	**79.4% [78.9%, 80%]**	**57.3% [56.8%, 57.8%]**	**4.34% [1.09%, 6.19%]**
5/30/21	*Observed*	**17.1**	**16.7**	**16.5**	**15.9**	**13.8**
	Predicted	**0.49 [0.48, 0.51]**	**1.61[1.58, 1.64]**	**3.4[3.35, 3.54]**	**7.3[7,23, 7.4]**	**14.77 [14.2, 15.5]**
	Averted	**16.6 [16.59, 16.62]**	**15.1[15.06, 15.12]**	**13.07[13, 13.15]**	**8.59[8.48, 8.67]**	**-0.97 [-1.7, -0.49]**
	% Reduction	**97.1% [97%, 97.2%]**	**90.4% [90.2%, 90.6%]**	**79.2% [78.5%, 79.7%]**	**54% [53.3%, 54.5%]**	**-7% [-12.4%, -3.5%]**
6/15/21	*Observed*	**18.7**	**18.3**	**18.1**	**17.5**	**15.3**
	Predicted	**0.51 [0.49, 0,53]**	**1.85[1.81, 1.89]**	**3.4 [3.34, 3.54]**	**9.5 [9.3, 9.7]**	**18.39 [17.55, 19.6]**
	Averted	**18.19[18.16, 18.20]**	**16.45 [16.41, 16.49]**	**13.07 [13.00, 13.16]**	**8.0 [7.8, 8.2]**	**-3.09 [-4.26, -2.25]**
	% Reduction	**97.3% [97.1%, 97.4%]**	**89.9% [89.7%, 90%]**	**79.2% [78.5%, 79.7%]**	**46% [44.7%, 46.7%]**	**-20.2% [-27.0%, -14.7%]**

Notes: Each cell reports (1) the total number of observed cases since the start of lockdown in the first row, (2) total number of predicted cases since the start of lockdown in the second row (with 95% CI), (3) the number of cases averted (relative to observed) since the start of lockdown in the third row (with 95% CI), and (4) the relative reduction in cases (as a percent) under lockdown in the fourth row (with 95% CI) from the intervention start date through the evaluation date. Numbers are reported in millions.

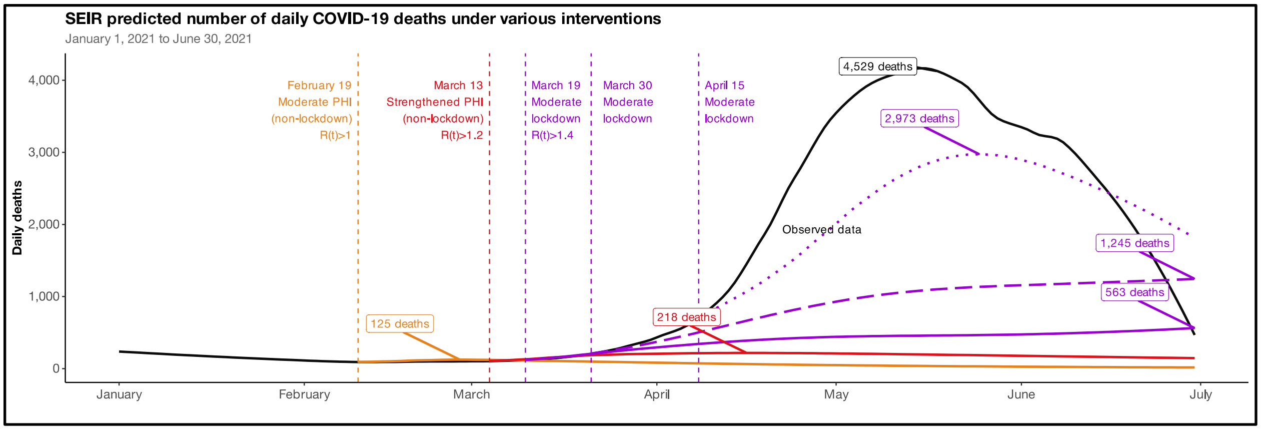

Figure S11. Observed (black) and SEIR-predicted daily reported death counts in India, under intervention scenarios starting on different dates from February 15 to June 30, 2021. Effect of the interventions are estimated from relative reduction in time-varying effective reproduction number from Maharashtra after strong intervention measures were implemented from March 28, with a lockdown starting April 14-June 7. Three intervention start dates are depicted: moderate PHI (non-lockdown; orange) measures on February 19 (when the trailing 7-day average R_t first crossed 1), strengthened PHI (non-lockdown; red) measures on March 13 (7-day R_t > 1.2) and moderate lockdown (solid purple) measures on March 19 (7-day R_t > 1.4). Delayed moderate lockdowns on March 30 (dashed purple) and April 15 (dotted purple) are also shown. Peak daily death counts under each intervention scenario are shown.

We see a similar trend in predicted reported death counts (**Figure S11**). Again, the early moderate and strengthened PHI scenarios avoid an increase in death counts along with the March 19 moderate lockdown. A slight, stable increase in deaths is seen under the March 30 moderate lockdown, though many fewer than daily reported death counts from the second wave in India. There is again a surge in deaths under the April 15 moderate lockdown scenario, though not quite a steep as what was observed.

An advantage of the SEIR model is that it can directly estimate the reported death counts, as opposed to the eSAIR model, which relies on the crude application of case-fatality rates to estimated daily case counts.

Table S10. SEIR-predicted total death counts, deaths averted and % reduction with corresponding 95% credible intervals under different lockdown interventions predicted by the SEIR model (in thousands)

Evaluation Date	Metrics	Moderate PHI (non-lockdown) start date February 19	Strengthened PHI (non-lockdown) start date March 13	Moderate lockdown start date March 19	March 30	April 15
3/30/21	*Observed*	**6.3**	**3.9**	2.9	-	-
	Predicted	**4.4 [4.3, 4.6]**	**3.35 [3.24, 3.47]**	2.87 [2.76, 2.98]		
	Averted	**1.86 [1.71, 2]**	**0.55 [0.43, 0.66]**	0.03 [-0.07, 0.14]		
	% Reduction	**29.5% [27.2%, 31.8%]**	**14.1% [11%, 17%]**	1.06% [-2.55%, 4.72%]		
4/15/21	*Observed*	**18.1**	**15.7**	**14.7**	**11.8**	-
	Predicted	**5.6 [5.5, 5.8]**	**6.8 [6.6, 6.9]**	**8.28 [8.09, 8.49]**	**8.3 [8.1, 8.5]**	
	Averted	**12.47 [12.29, 12.6]**	**8.9 [8.77, 9.1]**	**6.42 [6.21, 6.61]**	**3.5 [3.32, 3.7]**	
	% Reduction	**68.89% [67.88%, 69.86%]**	**56.9% [55.9%, 58.03%]**	**43.68% [42.27%, 45%]**	**29.8% [28.2%, 31.4%]**	
4/30/21	*Observed*	**55.6**	**53.2**	**52.2**	**49.3**	**37.5**
	Predicted	**6.5 [6.3, 6.7]**	**9.99 [9.77, 10.2]**	**14.5 [14.2, 14.9]**	**20.2 [19.9, 20.5]**	**22.7 [22.4, 23]**
	Averted	**49.1 [48.9, 49.33]**	**43.2 [43, 43.42]**	**37.7 [37.3, 38]**	**29.1 [28.8, 29.4]**	**14.8 [14.4, 15]**
	% Reduction	**87.97% [88.35%, 88.7%]**	**81.2% [80.8%, 81.62%]**	**72.1% [71.5%, 72.8%]**	**59% [58.3%, 59.7%]**	**39.3% [38.5%, 40.2%]**
5/15/21	*Observed*	**114.1**	**111.7**	**110.7**	**107.8**	**96.0**
	Predicted	**7.12 [6.89, 7.36]**	**13 [12.76, 13.34]**	**21.3 [20.8, 21.8]**	**35.4 [34.9, 35.9]**	**59.6 [58.6, 60.8]**
	Averted	**106.98 [106.7, 107.2]**	**98.64 [98.4, 98.9]**	**89.4 [88.9, 89.8]**	**72.4 [71.9, 72.9]**	**36.4 [35.1, 37.4]**
	% Reduction	**93.76% [93.55%, 93.96%]**	**88.3% [88.1%, 88.6%]**	**80.7% [80.3%, 81.1%]**	**67.1% [66.7%, 67.7%]**	**37.9% [36.6%, 38.9%]**
5/30/21	*Observed*	**172.9**	**170.5**	**169.5**	**166.6**	**154.8**
	Predicted	**7.6 [7.4, 7.9]**	**15.9 [15.5, 16.2]**	**28.27 [27.6, 29]**	**52.3 [51.6, 53]**	**103.7 [101.4, 107.2]**
	Averted	**165.3 [165, 165.5]**	**154.6 [154.3, 155]**	**141.2 [140.5, 142]**	**114.3 [113.6, 115]**	**51.1 [47.6, 53.4]**
	% Reduction	**95.6% [95.4%, 95,8%]**	**90.7% [90.5%, 91%]**	**83.3% [82.9%, 83.7%]**	**68.6% [68.2%, 69%]**	**33% [30.8%, 34.5%]**
6/15/21	*Observed*	**223.4**	**221.0**	**220.0**	**217.1**	**205.3**
	Predicted	**8 [7.73, 8.3]**	**15.88 [15.54, 16.2]**	**36 [35.1, 37.2]**	**71 [70.19, 72.2]**	**147 [142.2, 154]**
	Averted	**215.4 [215.1, 215.7]**	**154.6 [154.3, 155]**	**183.9 [182.8, 184.9]**	**146 [144.9, 146.9]**	**58.3 [51.4, 63]**
	% Reduction	**96.42% [96.3%, 96.5%]**	**90.7% [90.5%, 90.9%]**	**83.6% [83.1%, 84%]**	**67.3% [66.7%, 67.7%]**	**28.4% [25%, 30.7%]**

Notes: Each cell reports (1) the total number of observed deaths since the start of intervention in the first row, (2) total number of predicted deaths since the start of intervention in the second row (with 95% CI), (3) the number of deaths averted (relative to observed) since the start of intervention in the third row (with 95% CI), and (4) the relative reduction in cases (as a percent) under lockdown in the fourth row (with 95% CI) from the intervention start date through the evaluation date. Cells that are bolded represent a statistically significant reduction in the number of cases under intervention at the 95% credible interval level. Numbers are reported in thousands.

We can compare the SEIR-predicted reported death counts with those from the crude CFR (**Figure S12**) – i.e., by applying the same method for obtaining the death estimates in the main text (**Figure 4**). Panels A, B, and C (**Figure S12**) correspond to the high, moderate, and low CFR schedules (**Figure 2E**), respectively. Their numeric summaries are in **Tables S11**, **S12**, and **S13**, respectively. Across all CFR schedules, the models are predicting more deaths than in the analogous eSIR models albeit with exceptionally narrow credible intervals. Because of the way the CFR schedules are applied, they ignore the underlying transmission dynamics which give rise to the cases. In this sense, the deaths directly predicted by SEIR (**Figure S11**) represent a more natural distribution of deaths.

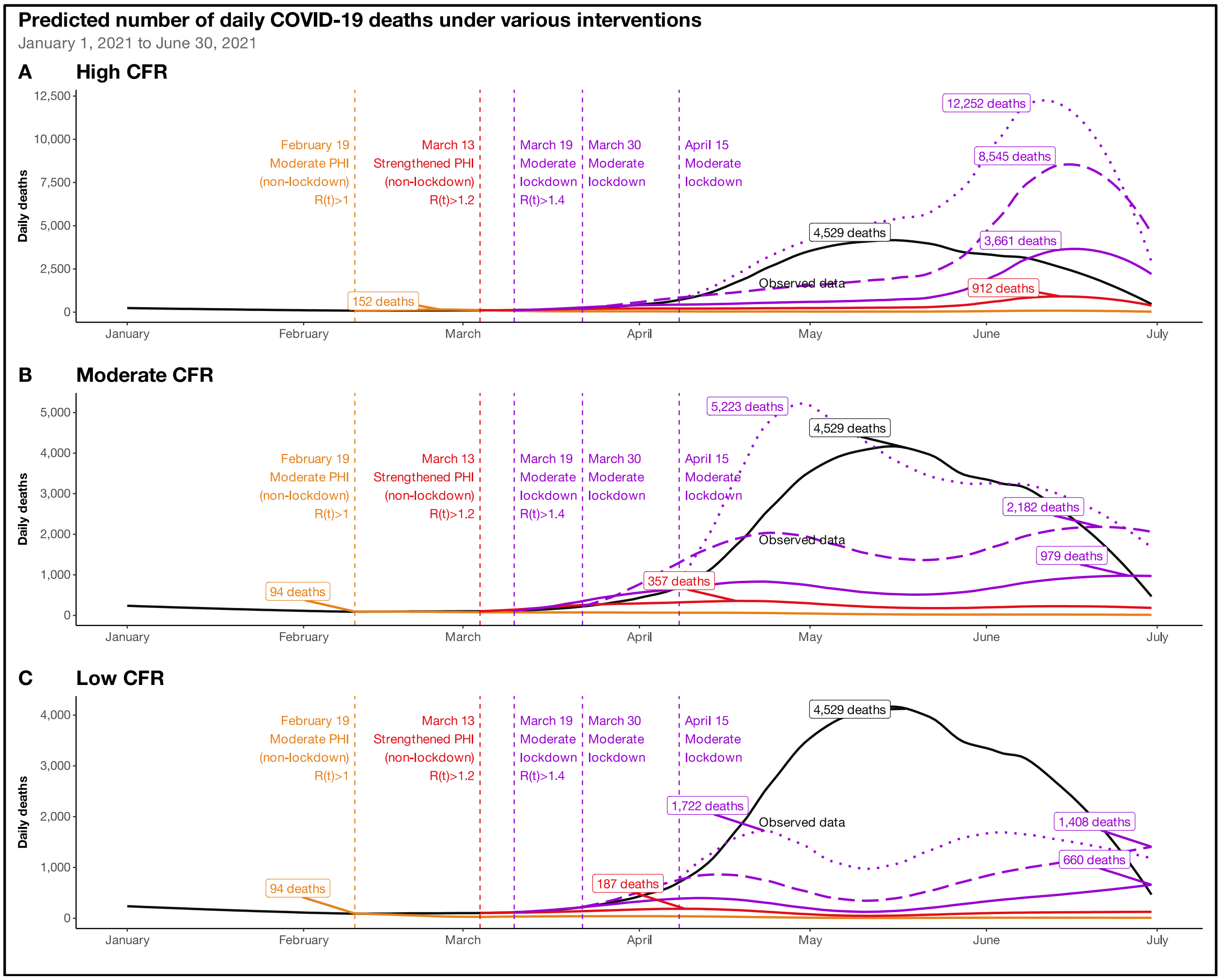

Figure S12. Observed (black) and predicted daily death counts in India, under intervention scenarios starting on different dates using high, moderate, and low CFR schedules observed from February 15 to June 30, 2021. Effect of the interventions are estimated from relative reduction in time-varying effective reproduction number from Maharashtra after strong intervention measures were implemented from March 28, with a lockdown starting April 14-June 7. High, moderate, and low CFR schedules are calculated from Maharashtra, India, and Kerala, respectively. The CFR schedule is derived from the daily 14-day case lagged CFR in India (i.e., $\mathrm{CFR_t} = \frac{\mathrm{deaths_t}}{\mathrm{cases_{t-14}}}$) which was then LOESS-smoothed using span = 0.3. The daily death estimates represent the SEIR-predicted reported daily case count multiplied by the respective CFR

schedule. Three intervention start dates are depicted: moderate PHI (non-lockdown; orange) measures on February 19 (when the trailing 7-day average R_t first crossed 1), strengthened PHI (non-lockdown; red) measures on March 13 (7-day R_t > 1.2) and moderate lockdown (solid purple) measures on March 19 (7-day R_t > 1.4). Delayed moderate lockdowns on March 30 (dashed purple) and April 15 (dotted purple) are also shown. Peak daily death counts under each intervention scenario are shown.

Table S11. Predicted total death counts, deaths averted and % reduction with corresponding 95% credible intervals under different lockdown interventions using SEIR-predicted cases and the high CFR schedule (in thousands)

Evaluation Date	Metrics	Moderate PHI (non-lockdown) start date	Strengthened PHI (non-lockdown) start date	Moderate lockdown start date		
		February 19	March 13	March 19	March 30	April 15
3/30/21	*Observed*	**6.3**	**3.9**	**2.9**	-	-
	Predicted	**3.87 [3.8, 3.96]**	**3.3 [3.26, 3.33]**	**3.9 [3.86, 3.96]**		
	Averted	**2.4 [2.3, 2.5]**	**0.6 [0.57, 0.64]**	**-1 [-1.06, -0.96]**		
	% Reduction	**38.5% [37.2%, 39.8%]**	**15.5% [14.7%,16.5%]**	**-34.8% [-36.7%, -33.2%]**		
4/15/21	*Observed*	**18.1**	**15.7**	**14.7**	**11.8**	-
	Predicted	**4.6 [4.48, 4.7]**	**6.7 [6.58, 6.76]**	**10.8 [10.6, 11]**	**14.2 [14, 14.3]**	
	Averted	**13.5 [13.4, 13.6]**	**9 [8.9, 9.1]**	**3.9 [3.7, 4.1]**	**-2.4 [-2.5, -2.2]**	
	% Reduction	**74.7% [74%, 75.3%]**	**57.5% [57%, 58%]**	**26.4% [24.9%, 27.6%]**	**-20.2% [-21.3%, -19%]**	
4/30/21	*Observed*	**55.6**	**53.2**	**52.2**	**49.3**	**37.5**
	Predicted	**5.2 [5, 5.3]**	**10.1 [9.9, 10.2]**	**19 [18.6, 19.4]**	**34 [33.6, 34.4]**	**48.9 [48.3, 50]**
	Averted	**50.1 [50, 50.3]**	**43.1 [43, 43.3]**	**33.2 [32.8, 33.6]**	**15.3 [14.9, 15.7]**	**-11.4 [-12.3, -10.8]**
	% Reduction	**90.7% [90.4%, 91%]**	**81% [80.7%, 81.3%]**	**63.7% [62.8%, 64.4%]**	**30.9% [30.2%, 31.8%]**	**-30.3% [-32.8%, -28.9%]**
5/15/21	*Observed*	**114.1**	**111.7**	**110.7**	**107.8**	**96.0**
	Predicted	**5.7 [5.5, 5.9]**	**13.8 [13.6, 14.1]**	**28.7 [28, 29.5]**	**60 [59.3, 60.8]**	**120.7 [118.2, 125]**
	Averted	**108.4 [108.2, 108.6]**	**97.8 [97.6, 98.1]**	**82 [81.2, 82.7]**	**47.7 [47, 48.4]**	**-24.7 [-29, -22.2]**
	% Reduction	**95% [94.8%, 95.2%]**	**87.6% [87.4%, 87.8%]**	**74.1% [73.3%, 74.7%]**	**44.3% [43.6%, 45%]**	**-25.7% [-30.2%, -23.1%]**
5/30/21	*Observed*	**172.9**	**170.5**	**169.5**	**166.6**	**154.8**
	Predicted	**6.3 [6.1, 6.5]**	**18.7 [18.3, 19]**	**43 [41.8, 44.5]**	**97.6 [96.4, 99.3]**	**211.3 [203.2, 223.3]**
	Averted	**166.6 [166.4, 166.8]**	**151.8 [151.4, 152.2]**	**126.5 [125, 128]**	**69 [67.2, 70.1]**	**-56.5 [-68.5, -48.4]**
	% Reduction	**96.4% [96.2%, 96.5%]**	**89% [88.8%, 89.2%]**	**74.6% [73.8%, 75.3%]**	**41.4% [40.4%, 42%]**	**-36.5% [-44.3%, -31.3%]**
6/15/21	*Observed*	**223.4**	**221.0**	**220.0**	**217.1**	**205.3**
	Predicted	**7.6 [7.3, 7.9]**	**31.4 [30.7, 32.2]**	**89.8 [86.9, 93.4]**	**210.4 [205.9, 218]**	**395.7 [369, 430]**
	Averted	**215.8 [215.5, 216.1]**	**189.6 [188.7, 190]**	**130.2 [126.6, 133.1]**	**6.7 [-0.94, 11.2]**	**-190 [-225, -164]**
	% Reduction	**96.6% [96.5%, 96.7%]**	**85.8% [85.4%, 86.1%]**	**57.5% [59.2%, 60.5%]**	**3.08% [-0.44%, 5.2%]**	**-92.7% [-110%, -80%]**

Notes: Each cell reports (1) the total number of observed deaths since the start of intervention in the first row, (2) total number of predicted deaths since the start of intervention in the second row (with 95% CI), (3) the number of deaths averted (relative to observed) since the start of intervention in the third row (with 95% CI), and (4) the relative reduction in cases (as a percent) under lockdown in the fourth row (with 95% CI) from the intervention start date through the evaluation date. Cells that are bolded represent a statistically significant reduction in the number of cases under intervention at the 95% credible interval level. Numbers are reported in thousands.

Table S12. Predicted total death counts, deaths averted and % reduction with corresponding 95% credible intervals under different lockdown interventions using SEIR-predicted cases and the moderate CFR schedule (in thousands)

Evaluation Date	Metrics	Moderate PHI (non-lockdown) start date	Strengthened PHI (non-lockdown) start date	Moderate lockdown start date		
		February 19	March 13	March 19	March 30	April 15
3/30/21	*Observed*	**6.3**	**3.9**	**2.9**	-	-
	Predicted	**3.1 [3.06, 3.21]**	**4.53 [4.5, 4.6]**	**5.3 [5.27, 5.41]**		
	Averted	**3.17 [3.09, 3.24]**	**-0.63 [-0.69, -0.58]**	**-2.43 [-2.51, -2.38]**		
	% Reduction	**50.2% [49%, 51.4%]**	**-16.2% [-17.5%, -15%]**	**-84% [-86.6%, -82%]**		
4/15/21	*Observed*	**18.1**	**15.7**	**14.7**	**11.8**	-
	Predicted	**4.2 [4.1, 4.3]**	**9.6 [9.5, 9.7]**	**15.8 [15.5, 16.1]**	**21.4 [21.2, 21.6]**	
	Averted	**13.9 [13.8, 14]**	**6.1 [6, 6.2]**	**-1 [-1.4 -0.82]**	**-9.6 [-9.8, -9.4]**	
	% Reduction	**76.8% [76.1%, 77.5%]**	**39% [38%, 40%]**	**-7.3% [-9.5%, -5.6%]**	**-81.7% [-83.4%, -79.8%]**	
4/30/21	*Observed*	**55.6**	**53.2**	**52.2**	**49.3**	**37.5**
	Predicted	**5.1 [4.9 5.3]**	**14.8 [14.6, 15]**	**28 [27.5, 28.8]**	**51.3 [50.7 51.9]**	**73.3 [72.5, 74.6]**
	Averted	**50.5 [50.3, 50.7]**	**38.4 [38.2, 38.7]**	**24.1 [23.4, 24.7]**	**-2 [-2.6, -1.4]**	**-35.8 [-37.1, -35]**
	% Reduction	**90.8% [90.5%, 91.2%]**	**72.2% [71.8%, 72.7%]**	**46.2% [44.8%, 47.3%]**	**-4.1% [-5.2%, -2.8%]**	**-95.5% [-99%, -93.4%]**
5/15/21	*Observed*	**114.1**	**111.7**	**110.7**	**107.8**	**96.0**
	Predicted	**5.6 [5.4, 5.8]**	**18.4 [18, 18.7]**	**37.3 [36.4, 38.3]**	**75.9 [74.9, 76.8]**	**141 [138.4, 145]**
	Averted	**108.5 [108.3, 108.7]**	**93.3 [93.1, 93.7]**	**73.4 [72.4, 74.3]**	**31.9 [31.1, 32.9]**	**-45 [-49.3, -42.4]**
	% Reduction	**95.1% [94.9%, 95.3%]**	**83.6% [83.3%, 84%]**	**66.3% [65.4%, 67.1%]**	**29.6% [28.8%, 30.5%]**	**-46.8% [-51.4%, -44.2%]**
5/30/21	*Observed*	**172.9**	**170.5**	**169.5**	**166.6**	**154.8**
	Predicted	**5.9 [5.7, 6.2]**	**21 [20.7, 21.5]**	**45.3 [44.2, 46.7]**	**97 [96, 98]**	**192.3 [187, 201]**
	Averted	**167 [166.7, 167.2]**	**149.4 [149, 150]**	**124.2 [122.8, 125.3]**	**69.7 [68.4, 70.8]**	**-37.5 [-46.2, -32]**
	% Reduction	**96.6% [96.4%, 96.7%]**	**87.7% [87.4%, 87.9%]**	**73.3% [72.5%, 74%]**	**41.8% [41.1%, 42.5%]**	**-24.2% [-29.8%, -20.7%]**
6/15/21	*Observed*	**223.4**	**221.0**	**220.0**	**217.1**	**205.3**
	Predicted	**6.3 [6.1, 6.6]**	**24.6 [24.1, 25.1]**	**58 [56.4, 60]**	**127.8 [126.2, 130.6]**	**243.6 [233, 259]**
	Averted	**217 [216.9, 217.4]**	**196.4 [195.9, 197]**	**162 [160, 163.6]**	**89.2 [86.4, 90.8]**	**-38.3 [-53.2, -27.7]**
	% Reduction	**97.2% [97.1%, 97.3%]**	**88.9% [88.7%, 89.1%]**	**73.6% [72.7%, 74.3%]**	**41.1% [39.8%, 41.8%]**	**-18.7% [-25.9%, -13.5%]**

Notes: Each cell reports (1) the total number of observed deaths since the start of intervention in the first row, (2) total number of predicted deaths since the start of intervention in the second row (with 95% CI), (3) the number of deaths averted (relative to observed) since the start of intervention in the third row (with 95% CI), and (4) the relative reduction in cases (as a percent) under lockdown in the fourth row (with 95% CI) from the intervention start date through the evaluation date. Numbers are reported in thousands.

Table S13. Predicted total death counts, deaths averted and % reduction with corresponding 95% credible intervals under different lockdown interventions using SEIR-predicted cases and the low CFR schedule (in thousands)

Evaluation Date	Metrics	Moderate PHI (non-lockdown) start date	Strengthened PHI (non-lockdown) start date	Moderate lockdown start date		
		February 19	March 13	March 19	March 30	April 15
3/30/21	*Observed*	**6.3**	**3.9**	**2.9**	-	-
	Predicted	**1.4 [1.38, 1.45]**	**2.45 [2.41, 2.48]**	**2.96 [2.93, 3]**		
	Averted	**4.89 [4.85, 4.92]**	**1.45 [1.42, 1.48]**	**-0.06 [-0.1, -0.03]**		
	% Reduction	**77.6% [77%, 78%]**	**37.2% [36.6%, 38%]**	**-2.12% [-3.58%, -0.94%]**		
4/15/21	*Observed*	**18.1**	**15.7**	**14.7**	**11.8**	-
	Predicted	**2 [1.97, 2.1]**	**5.4 [5.3, 5.5]**	**9 [8.8, 9.2]**	**12.3 [12.2, 12.4]**	
	Averted	**16.06 [16, 16.1]**	**10.3 [10.25, 10.4]**	**5.7 [5.5, 5.9]**	**-0.49 [-0.6, -0.36]**	
	% Reduction	**88.8% [88.4%, 89.1%]**	**65.8% [65.3%,66.2%]**	**39% [37.7%, 40%]**	**-4.2% [-5.1%, -3.1%]**	
4/30/21	*Observed*	**55.6**	**53.2**	**52.2**	**49.3**	**37.5**
	Predicted	**2.4 [2.3, 2.5]**	**7.3 [7.2, 7.4]**	**13.6 [13.3, 13.9]**	**23.4 [23.1, 23.6]**	**27.2 [26.9, 27.6]**
	Averted	**53.2 [53.1, 53.3]**	**45.9 [45.8, 46]**	**38.6 [38.3, 38.9]**	**25.9 [25.7, 26.2]**	**10.3 [9.9, 10.6]**
	% Reduction	**95.7% [95.6%, 95.9%]**	**86.2% [86%, 86.4%]**	**74% [73.4%, 74.5%]**	**52.6% [52.1%, 53.2%]**	**27.6% [26.4%, 28.3%]**
5/15/21	*Observed*	**114.1**	**111.7**	**110.7**	**107.8**	**96.0**
	Predicted	**2.5 [2.4, 2.6]**	**8.2 [8, 8.3]**	**15.7 [15.4, 16.1]**	**29 [28.7, 29.4]**	**42.8 [42.2, 44]**
	Averted	**111.6 [111.5, 111.7]**	**103.6 [103.4, 103.7]**	**95 [94.6, 95.3]**	**78.7 [78.4, 79.1]**	**53.2 [52, 53.8]**
	% Reduction	**97.8% [97.7%, 97.9%]**	**92.7% [92.6%, 92.8%]**	**85.8% [85.5%, 86.1%]**	**73% [72.8%, 73.4%]**	**55.4% [54.2%, 56.1%]**
5/30/21	*Observed*	**172.9**	**170.5**	**169.5**	**166.6**	**154.8**
	Predicted	**2.6 [2.5, 2.7]**	**9.3 [9.1, 9.4]**	**19 [18.6, 19.5]**	**37.7 [37.3, 38.1]**	**63.5 [61.6, 66.5]**
	Averted	**170.3 [170.2, 170.4]**	**161.2 [161, 161.4]**	**150.5 [150, 151]**	**128.9 [128.5, 129.3]**	**91.3 [88.3, 93.2]**
	% Reduction	**98.5% [98.4%, 98.54%]**	**94.6% [94.5%, 94.7%]**	**88.8% [88.4%, 89%]**	**77.4% [77.1%, 77.6%]**	**59% [57%, 60.2%]**
6/15/21	*Observed*	**223.4**	**221.0**	**220.0**	**217.1**	**205.3**
	Predicted	**2.8 [2.7, 2.9]**	**11.04 [10.8,11.3]**	**25.5 [24.8, 26.3]**	**53.3 [52.7, 54.6]**	**89.7 [85.2, 95.8]**
	Averted	**220.6 [220.5, 220.7]**	**210 [209.7, 210.2]**	**194.5 [193.7, 195.2]**	**163.8 [162.5, 164.4]**	**115.6 [109, 120]**
	% Reduction	**98.7% [98.7%, 98.8%]**	**95% [94.9%, 95.1%]**	**88.4% [88%, 88.7%]**	**75.4% [74.9%, 75.8%]**	**56.3% [53.4%, 58.5%]**

Notes: Each cell reports (1) the total number of observed deaths since the start of intervention in the first row, (2) total number of predicted deaths since the start of intervention in the second row (with 95% CI), (3) the number of deaths averted (relative to observed) since the start of intervention in the third row (with 95% CI), and (4) the relative reduction in cases (as a percent) under lockdown in the fourth row (with 95% CI) from the intervention start date through the evaluation date. Cells that are bolded represent a statistically significant reduction in the number of cases under intervention at the 95% credible interval level. Numbers are reported in thousands.

We have also conducted a SEIR analog to the eSIR-based length of lockdown analysis (**Figure S8**) in **Figure S13**. We see that the results are essentially identical over the first four weeks of prediction, as is expected. A 4-week lockdown results in elevated case counts over the prediction period while the longer scenarios further suppress case counts. There is not much apparent benefit in extending the lockdown from 6-weeks to 8-weeks. In all length of lockdown scenarios, an April 15 lockdown is too late to avoid a second wave.

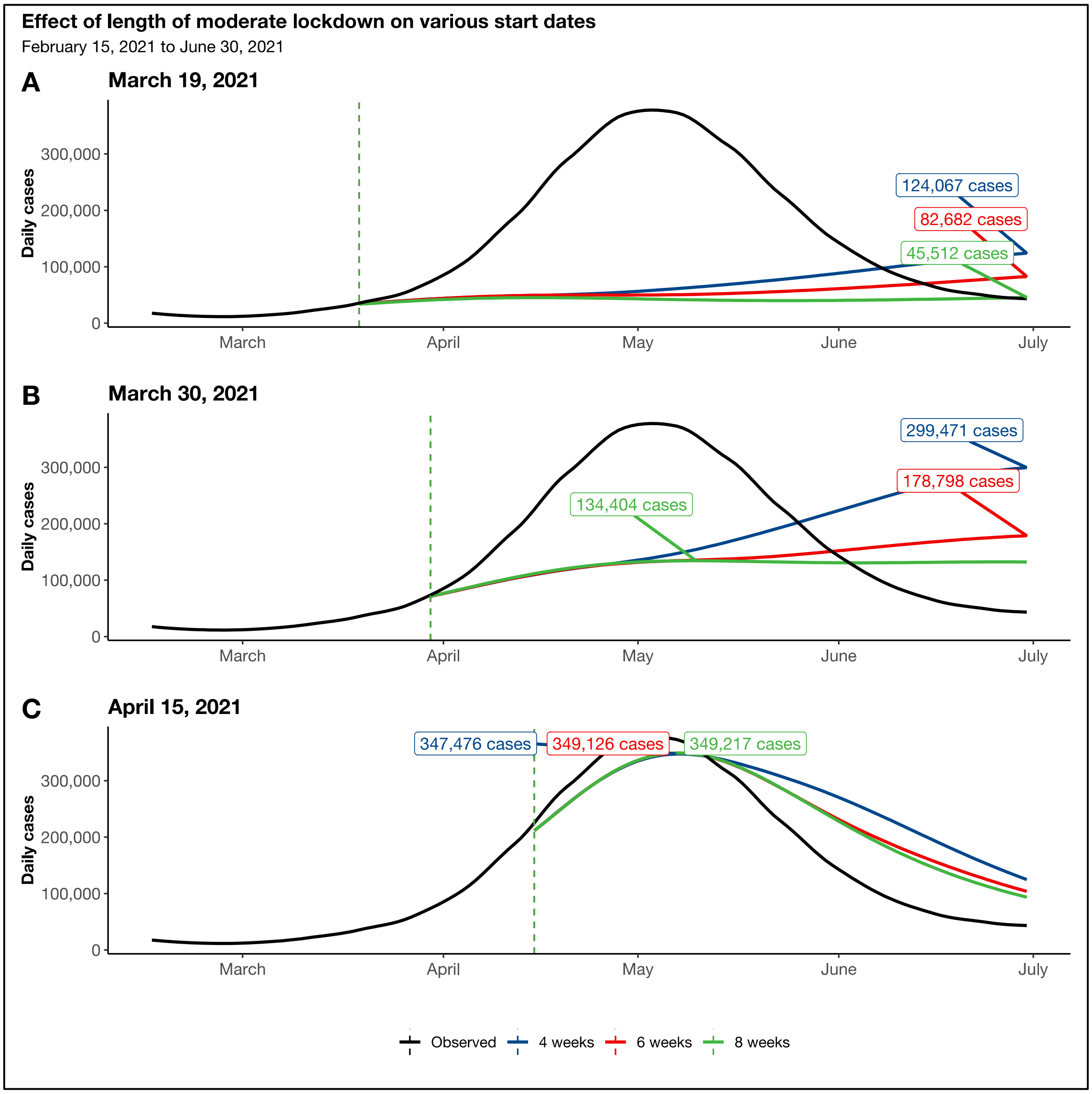

Figure S13. Comparing the effect of strengths and SEIR-modeled lengths of lockdown on COVID-19 cases in India had they been instituted nationwide on April 15, 2021. The time of "unlock" is varied in each scenario. Locking and unlocking effects are adapted from Maharashtra in 2021 (moderate lockdown).

These SEIR-based length of lockdown results differ. The eSAIR results (**Figure S8**) predict a far more subdued and suppressed trajectory for both the March 30 and April 15

moderate length of lockdown analyses. We also see that the SEIR-based length of lockdown analysis predicts a larger increase in cases through the prediction period, regardless of lockdown length, for a moderate lockdown starting March 19 than the eSAIR-based results.

Section 4. Impact of PHI on hospital and intensive care units bed capacity

One of the central goals of PHI is to “flatten the curve” – that is, to keep the number of COVID-19 cases within the capacity of the health system to adequately care for individuals requiring hospitalization of intensive care. We therefore conducted an analysis to understand whether PHI implemented at various dates could have prevented the health system capacity from being overwhelmed in India’s second COVID-19 wave (**Figure S14**). The area shaded in red under the observed needs for hospital and ICU beds (black line) shows the extent to which these needs exceeded health system capacity in India’s second wave. If implemented early enough in February or March 2021, moderate PHI (non-lockdown), strengthened PHI (non-lockdown), and moderate lockdown each would have prevented enough COVID-19 cases to keep hospitalization and ICU needs well within the capacity of India’s health system. However, if implementation of moderate lockdown were delayed until April 15, 2021, hospital bed and ICU capacity would have been greatly exceeded, as indicated by the shaded red area under the dotted purple line.

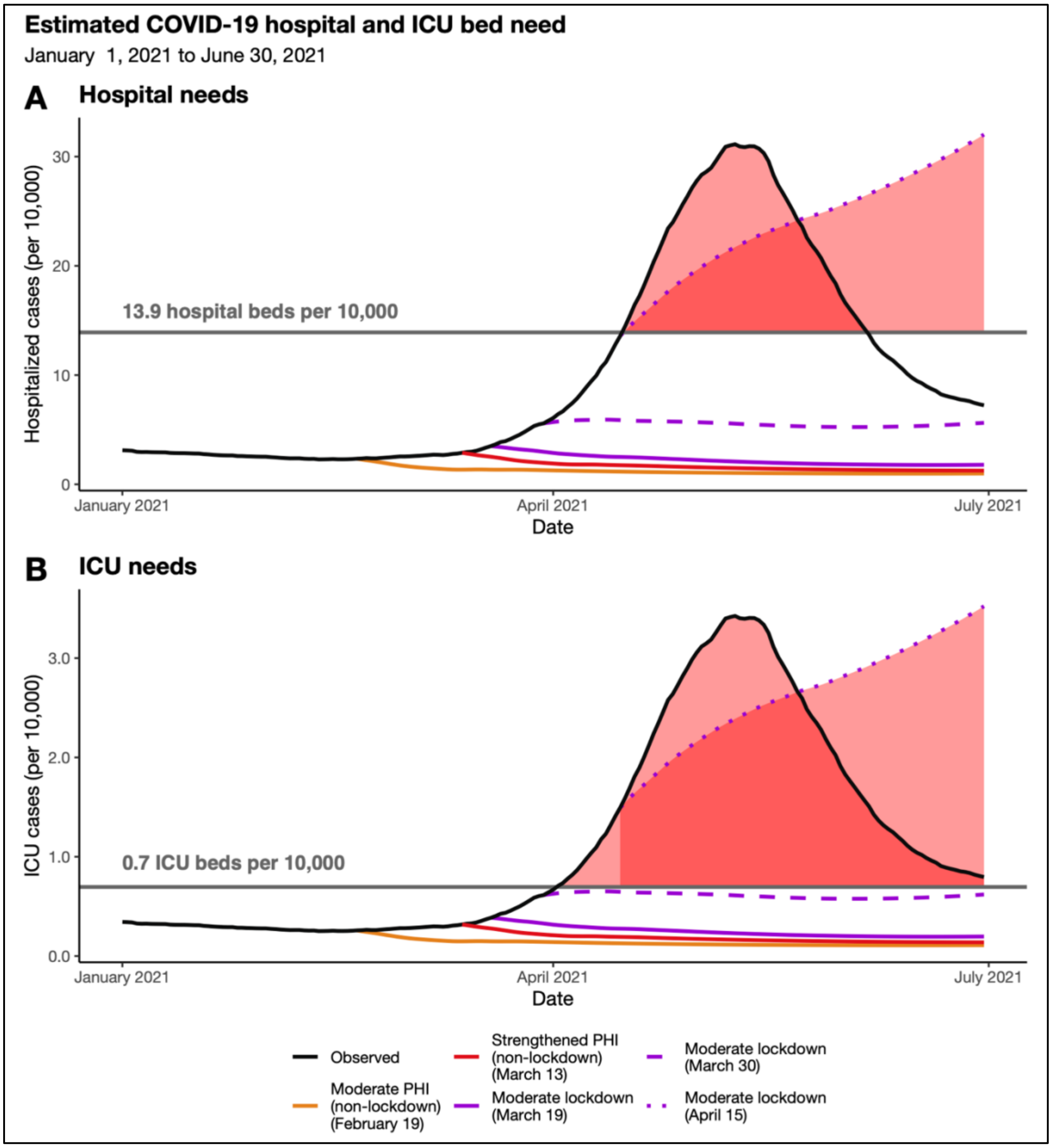

Figure S14. Impact of public health interventions (PHI) in the context of estimated hospital bed capacity (panel A) and intensive care unit (ICU) bed capacity (panel B) in India. The horizontal line (gray) represents estimated hospital or ICU bed capacity per 10,000 people in India. The observed need (black) is estimated for hospital beds assuming that 7.8% of active COVID-19 cases require hospitalization (*46*) and for ICU beds assuming that 11% of those requiring hospitalization merit ICU care (*47*), both reported per 10,000 cases. Active COVID-19 cases were estimated as comprising the difference in cumulative confirmed cases from cumulative recovered cases. Projections for cases requiring hospitalization or ICU care are presented under scenarios in which moderate PHI (non-lockdown) are implemented on February 19, 2021 (orange), strengthened PHI (non-lockdown) are implemented on March 13, 2021 (red), moderate lockdown is implemented on March 19, 2021 (solid purple), moderate lockdown is implemented on March 30, 2021 (dashed purple), and moderate lockdown is implemented on April 15, 2021 (dotted purple). Area shaded in red indicates scenarios under which hospital or ICU bed capacity is exceeded.

We have also done the same hospital and ICU need estimation from the SEIR models (**Figure S15**), assuming the same hospitalization rate and ICU rate as presented in **Figure S14**. The active cases in the SEIR model are given directly by the P compartment (model schematic in **Figure S9**). The results are like the eSIR model, though the moderate lockdown on March 30 results in a surge that exceeds hospital and ICU capacity. From the April 15 model (dotted purple), we see that while the peak *daily reported case count* is not as high as observed (**Figure S10**), the resulting estimated number of cases requiring concurrent hospitalization (**Figure S15A**), or ICU care (**Figure S15B**) is substantially higher than what was observed. It is important to note that hospital and ICU needs are a function of *active* cases rather than *daily* cases. Active cases are all individuals with an active, ongoing infection while daily cases are only those individuals who are newly diagnosed (i.e., ignores recently diagnosed cases who have not yet recovered). This suggests that even if an intervention would have reduced or flattened a surge in the way a moderate lockdown on March 30, 2021, or April 15, 2021, would have (according to SEIR predicted cases in **Figure S10**), the health system capacity would still be exceeded. In these scenarios, cases that would have otherwise received hospitalized or ICU care wouldn't, resulting in avoidable adverse outcomes.

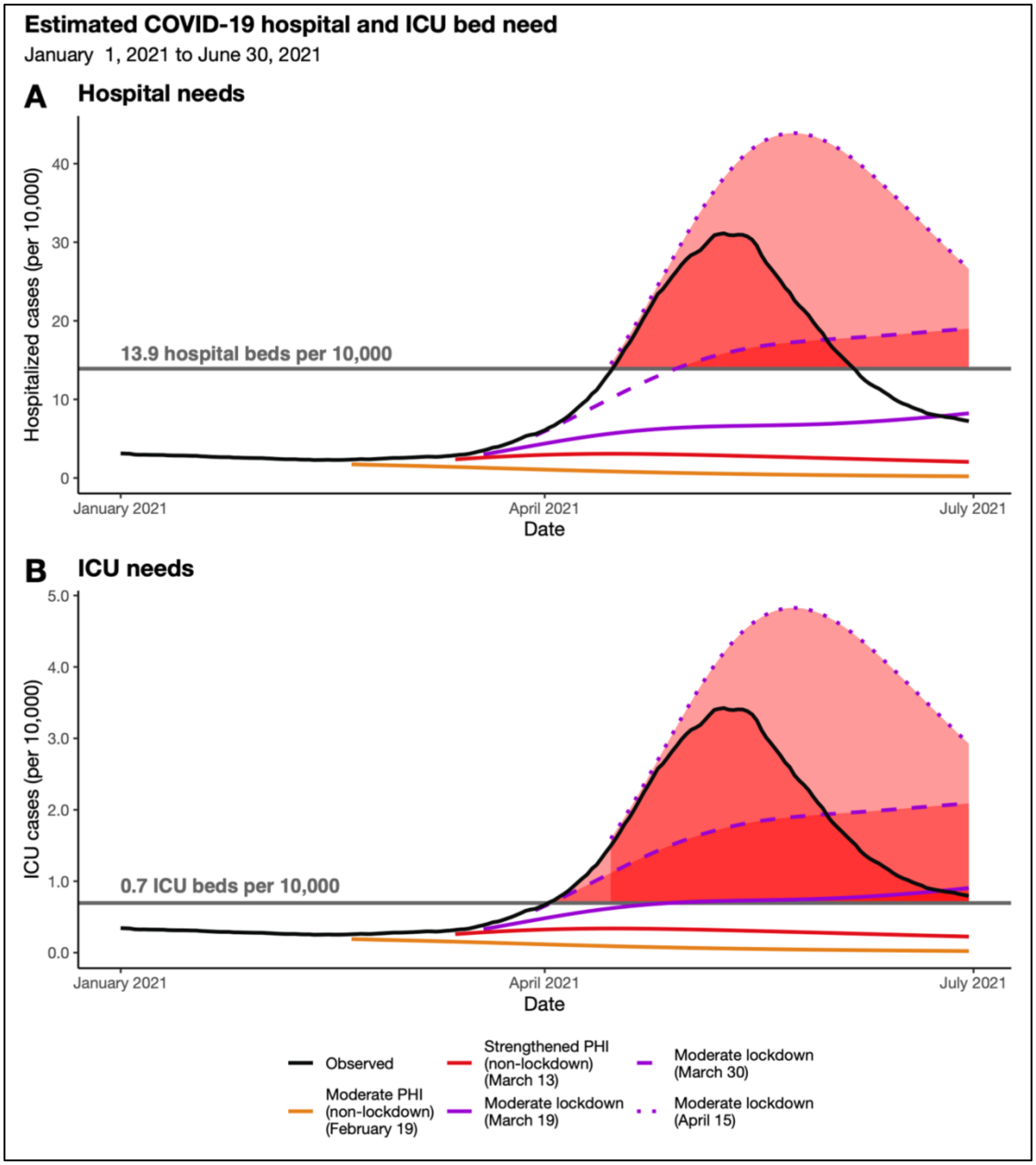

Figure S15. Impact of public health interventions (PHI) in the context of estimated hospital bed capacity (panel A) and intensive care unit (ICU) bed capacity (panel B) in India. The horizontal line (gray) represents estimated hospital or ICU bed capacity per 10,000 people in India. The observed need (black) is estimated for hospital beds assuming that 7.8% of active COVID-19 cases require hospitalization (*46*) and for ICU beds assuming that 11% of those requiring hospitalization merit ICU care (*47*), both reported per 10,000 cases. Active COVID-19 cases were estimated as comprising the difference in cumulative confirmed cases from cumulative recovered cases with estimates from the SEIR model. Projections for cases requiring hospitalization or ICU care are presented under scenarios in which moderate PHI (non-lockdown) are implemented on February 19, 2021 (orange), strengthened PHI (non-lockdown) are implemented on March 13, 2021 (red), moderate lockdown is implemented on March 19, 2021 (solid purple), moderate lockdown is implemented on March 30, 2021 (dashed purple), and moderate lockdown is implemented on April 15, 2021 (dotted purple). Area shaded in red indicates scenarios under which hospital or ICU bed capacity is exceeded.

Section 5. Development of the tiered COVID-19 response framework and data needs to improve future surveillance

Development of the tiered COVID-19 response framework (**Table 3**) involved a few steps: (1) review of other tiered COVID-19 response frameworks, including those produced by the World Health Organization (WHO) (*48*), Ontario (Canada) (*49*), Scotland (*50*), and Singapore (*51*); (2) refinement of proposed PHI in different tiers based on key principles derived from recent evidence on transmission risk in different settings; (3) mapping of PHI and escalation tiers to interventions used in the modeling analysis; and (4) determination of appropriate threshold ranges for epidemiological indicators (e.g., R_t and test positive rates) that should trigger escalation to the next tier of PHI in a manner informed by the model. More specifically, our R_t and test positive rate thresholds were informed by values of these indicators in Maharashtra or India more generally around dates of implementation of corresponding interventions in the model. We describe this process in more detail below.

(1) *Review of other tiered COVID-19 response frameworks*

We reviewed a recent policy brief that evaluated multiple tiered PHI escalation frameworks (*98*). That policy brief recommended the Ontario framework for visualizing these tiered strategies given its user friendliness, as well as the range of parameters included in this framework (epidemiological thresholds, health system capacity, public health system capacity). We simplified the structure of the Ontario framework to four color-coded and graded responses and included PHI components, surveillance measures, and other components from similar frameworks produced by the WHO, Scotland, and Singapore. We contextualized PHI to address activities that are central to Indian society (e.g., weddings, household help, auto-rickshaw as a mode of transportation, etc.) and included additional guidance on anticipatory surveillance and care delivery preparations.

(2) *Refinement of proposed PHI based on evidence on transmission risk in different settings*

We then revised our framework to reflect key principles based on recent evidence on transmission risk in different settings. For example, based on evidence that outdoor transmission is infrequent (*99*, *100*), one principle across all PHI tiers is the importance of risk reduction in indoor settings. Consistent with this principle, a major goal is to move people and activities outdoors when possible. In the Indian context, one implication of this principle is that authorities should shift their focus away from punitive measures against individuals and small groups who are outside, in favor of ensuring indoor establishments are compliant with PHI. Another implication is that non-congregate physical activity and exercise outdoors should be allowed and even encouraged across all tiers.

A second principle builds upon research suggesting that transmission risk varies in different indoor settings (*101*). As such, in our framework, settings in which individuals are more likely to be unmasked and have prolonged contact time with others (e.g., restaurants, weddings, religious gatherings) and larger congregate venues (e.g.,

cinemas) are considered higher risk indoor settings. In contrast, settings in which individuals are more likely to be masked with shorter contact time with any given individual (e.g., stores) are considered lower risk indoor settings. One implication of this principle is that earlier tiers might focus on escalating PHI in higher risk indoor settings, while subsequent tiers might extend PHI across most indoor settings.

A third principle is the importance of prioritizing people's ability to access essential needs—such as water, food, and health care—across all tiers. India's nationwide lockdown in 2020 shut down most forms of public transportation and inadvertently resulted in substantial drops in care seeking for other medical conditions. For example, the number of people reported as being treated for tuberculosis dropped by 50% (*14*), and HIV testing dropped by more than 80% at some facilities (*13*). As such, we emphasize the importance of allowing public and private transportation, even in the tier with maximal PHI, with mitigation measures such as masking and capacity limits. In addition, shifting to a higher tier (e.g., tier 2) should immediately prompt health system preparations for potential escalation to the subsequent tier (e.g., tier 3). We have outlined examples of possible health system preparatory measures in the table.

A fourth principle undergirding this framework is that escalation in PHI should be accompanied by augmented social protections. During earlier tiers, the central and state government should expand existing safety net programs to make them more robust, in anticipation of challenges that could be faced with escalation of PHI. In the tier with maximal PHI, governments should focus on disbursing rapid financial relief to affected businesses, food for vulnerable populations, and cash transfers to large segments of the population who may be temporarily unemployed. We have previously argued that these periods of lockdown should be reframed using the term "humane shelter at home" (HuSH), to indicate that social protections are central to this intervention, rather than just an afterthought (*61*).

(3) *Mapping of PHI and escalation tiers to interventions used in the modeling analysis*

We then mapped PHI for particular tiers in our framework to generally correspond to interventions that were implemented in the modeling analysis. For example, in the modeling analysis, "strengthened PHI (non-lockdown)" represents the effect of a variety of interventions implemented from March 28, 2021, to April 13, 2021, in Maharashtra state, as described previously in **Table S4**. The specific interventions implemented during that time period in Maharashtra correspond very closely to the interventions proposed in Tier 3 of our COVID-19 response framework, as shown in **Table S14**.

Similarly, in the modeling analysis, "moderate lockdown" represented the effect of the lockdown implemented in Maharashtra from April 14, 2021, to June 7, 2021. This lockdown involved all of the interventions implemented from March 28, 2021, to April 13, 2021; however, in addition, everyone had to shelter at home and could only leave to fulfill essential needs. These moderate lockdown interventions correspond very closely to the interventions proposed in Tier 4 in our framework. For example, in Tier 4 of our

framework, all non-essential workplaces and shopping establishments are shut down, leaving few indoor spaces available for interactions outside of the home.

Table S14. Comparison of public health interventions (PHIs) between "strengthened PHI (non-lockdown)" in the modeling analysis and Tier 3 in the COVID-19 response framework

Type of intervention setting	Components of "strengthened PHI (non-lockdown)" implemented from March 28, 2021, to April 13, 2021, in Maharashtra	Proposed components of Tier 3 in the COVID-19 response framework
Restaurants, bars, coffee/tea shops, gyms	Shut down of "90% of restaurants," but food pickup and delivery were allowed.[1] Night curfew was implemented to reduce congregation in bars and restaurants.[2]	Closure of indoor dining, but food pickup and delivery is allowed.
Weddings and social, political, and religious gatherings	Ban on most large gatherings but moderate limitations on weddings to 50 people.[3-5] Places of worship closed.[1]	Stringent indoor capacity limits, e.g., not more than 5 to 10 people
Workplaces	Workplace capacity was reduced, and private offices eventually closed; 50% capacity limits instituted in government offices[1]	Institution of indoor capacity limits
Non-essential stores	• Capacity limits in malls and other shopping establishments[1] with eventual closure of all non-essential stores[6]<br>• Theaters and multiplexes remain closed.[1]<br>• Hair salons and beauty parlors to remain closed[6]	Institution of indoor capacity limits
Transportation	Trains, buses, taxis, and autos allowed to operate at 50% capacity[1]	Institution of capacity limits
Additional interventions that do not align directly	Weekend lockdown (i.e., shelter at home) [1]	Reduced frequency of visits for non-live-in domestic workers for non-essential tasks.
		Public communication that families should not visit any other household or allow visitors into their homes
	Section 144 enforced: During the day cannot be seen in groups larger than 5 people[6]	
	Schools and colleges remain closed[6]	

The references for **Table S14** are available in the supplemental reference **Data File S1**.

Tier 2 in our COVID-19 response framework comprises PHI that are less intensive than in Tier 3. There was no intervention period in Maharashtra that mapped well to Tier 2 in our COVID-19 response framework. As such, "moderate PHI (non-lockdown)"—in which the intervention effect is attenuated by 20% compared to strengthened PHI (non-lockdown) or Tier 3—is meant to correspond to the less intensive interventions in Tier 2.

Notably, the 2020 national lockdown in India—which lasted from March 25, 2020, with staged reopening starting in May 2020—had a greater intensity of PHI than the 2021 lockdown in Maharashtra. As such, the intervention effect of the 2020 national lockdown does not map onto any of the tiers of our proposed in our COVID-19 response framework. As per news reports and government documents, the 2020 national lockdown was like the 2021 lockdown in Maharashtra, in that it involved a mandatory order that everyone in the country should shelter at home, outside of leaving for essential activities. The 2020 national lockdown was more stringent than the 2021 Maharashtra lockdown, however, in that the national lockdown involved suspension of nearly all public and private

transportation, except for transporting goods and emergency services (*102–105*). This resulted in prohibition of movement of most people who were not essential workers, while allowing movement of goods. Although the 2021 Maharashtra lockdown did eventually involve a briefer restriction of interstate and interdistrict travel (*106*), some forms of public transportation locally continued at reduced capacity (*19*). A more intangible difference between the 2020 national lockdown and the 2021 Maharashtra lockdown may have been the intensity of enforcement, although this is difficult to measure. The national lockdown outlined stringent punishments for violations of government PHI orders (*103*) and took place early in the COVID-19 pandemic, when there was less public fatigue with PHI and potentially more political will to enforce the order.

(4) *Determination of appropriate threshold ranges for epidemiological indicators that should trigger escalation based on the modeling analysis*:

In the modeling analysis, application of different levels of interventions implemented on specific dates resulted in considerable reductions in COVID-19 cases and death. For example, moderate PHI (non-lockdown) were implemented on February 19, 2021—when the 7-day trailing average for R_t crossed 1—corresponding to the anticipated effects of Tier 2 PHI in our framework. Strengthened PHI (non-lockdown) was implemented on March 13, 2021—when the 7-day trailing average for R_t crossed 1.2—corresponding to the anticipated effects of Tier 3 PHI in our framework. Moderate lockdown was implemented at the earliest in the model on March 19, 2021—when the 7-day trailing average for R_t crossed 1.4—corresponding to the anticipate effects of Tier 4 PHI in our framework. These R_t thresholds therefore directly informed escalation thresholds to escalate to these tiers in our PHI framework. We then compiled test positive rates for both Maharashtra and India around these dates. Based on this information, we determined pragmatic ranges of test positive rates and other indicators, in addition to R_t, that should inform escalation to the next tier of PHI, if the framework were to be used prospectively.

Considering the potential role of real-time epidemiological indicators for triggering escalations in PHI, enhancing the quality of data and information available for surveillance will be critical to improve the future public health response (**Table S15**). Many of the data needs described below directly link to epidemiological indicators in our tiered COVID-19 response framework. Other information—such a regular nationally representative sample of genome sequencing data—have the potential to facilitate early identification of new variants that could fundamentally shift the trajectory of the pandemic.

Table S15. Data and information needs to improve future surveillance

Disaggregated daily case, death, test, and vaccination counts by age and sex at a district level. Date of specimen collection for tests and date of death (not the date of report) will lead to improved estimation of transmission model parameters due to lagged effects
Nationally representative sample of genome sequencing data every week, along with uploading and sharing data to GISAID or other international platform
Number of hospitalizations at a state/district level
Number and % of hospital and ICU beds available at a district level
Seroprevalence results at a state/district level with representative sampling
Percent of population fully vaccinated and partially vaccinated at a district level
Epidemiological metrics: 7-day average test-positive rate, case-fatality rate, R_t, % increase in deaths during the time cases are doubled, projections for the next 2-4 weeks under different scenarios
All-cause and cause-specific mortality data disaggregated by age and sex at a district level
Through linkage of the sequencing, testing, and vaccination databases, provide estimates of re-infection, cluster infection and breakthrough infections (and hospitalizations related to each)
Antibody levels in a representative sample at regular intervals (e.g., every 3 months) post-vaccination

Section 6. Additional material for discussion

Table S16A. Papers estimating the effect of non-pharmaceutical interventions on COVID-19 transmission and/or cases across multiple countries

Citation	Reference number	Data	Location	Date range	Intervention	Estimate	Metric
Banholzer et al. (2021). PLoS One doi: 10.1371/journal.pone.0252827	(*107*)	Johns Hopkins Coronavirus Research Center	US, Canada, Australia, and 15 European countries	February 2020 through May 2020	Bans of large gatherings<br>Venue closures<br>School closures<br>Border closures<br>Bans of small gatherings<br>Stay-at-home orders<br>Work-from-home orders	37% (21%, 50%)<br>18% (-4%, 40%)<br>17% (-2%, 36%)<br>10% (-2%, 36%)<br>9% (-4%, 23%)<br>4% (-6%, 17%)<br>1% (-8%, 12%)	Relative reduction in new infections (95% Credible Interval)
Brauner et al. (2021) Science doi: 10.1126/science.abd9338	(*108*)	COVID-19 cases and deaths from Johns Hopkins Center for Systems Science and Engineering (CSSE) COVID-19 Dataset	41 countries (Malaysia and Singapore only Asian countries)	January 22, 2020 through May 30 2020	Gatherings limited to 1000 people or less<br>Gatherings limited to 100 people or less<br>Gatherings limited to 10 people or less<br>Some businesses closed<br>Most nonessential business closed<br>Schools and universities closed<br>Additional effect of a stay-at-home order on top of other interventions	23% (0%, 40%)<br>34% (12%, 52%)<br>42% (17%, 60%)<br>18$ (-8%, 40%)<br>27% (-3%, 49%)<br>38% (16%, 54%)<br>13% (-5%, 31%)	Relative reduction in time-varying reproduction number R_t (95% prediction interval)
Pei et al. (2020) Science Advances doi: 10.1126/sciadv.abd6370	(*65*)	US county-level case and death data from USAFacts	US (county-level)	March 15, 2020 through May 3, 2020	Observed interventions one week earlier<br>Observed interventions two weeks earlier	53% (41%, 63%)<br>73% (59%, 80%)	Relative reduction in cases through May 3 (95% credible interval)
Talic et al. (2021) BMJ doi: 10.1136/bmj-2021-068302	(*109*)	Meta-analysis of eight studies	Multiple	Varies	Handwashing<br>Mask wearing<br>Physical distancing	53% (-12%, 81%)<br>53% (25%, 71%)<br>25% (5%, 41%)	Relative reduction in incidence of cases (95% credible interval)
Bo et al. (2021) IJID doi: 10.1016/j.ijid.2020.10.066	(*64*)	Johns Hopkins Center for Systems Science and Engineering	190 countries (including India)	January 23, 2020 through April 13, 2020	Mandatory face mask in public<br>Isolation or quarantine<br>Social distancing<br>Traffic restriction	15% (8%, 22%)<br>11% (9%, 14%)<br>43% (42%, 44%)<br>9% (7%, 11%)	Relative reduction in time-varying reproduction number R_t (95% confidence interval)
Barros et al. (2022) medRxiv doi: 10.1101/2022.02.28.22271671	(*110*)	World Health Organization Coronavirus (COVID-19) Dashboard	113 countries (including India)	January 2020 through May 2020	School closures<br>Work-from-home orders<br>Mask wearing	19% (2%, 37%)<br>19% (12%, 27%)<br>16% (7%, 25%)	Relative reduction in time-varying reproduction number R_t (95% confidence interval)
Flaxman et al. (2020) Nature doi: 10.1038/s41586-020-2405-7	(*111*)	European Centre for Disease Prevention and Control	11 European countries	Through May 4, 2020	Lockdown	81% (75%, 87%)	Relative reduction in time-varying reproduction number R_t (95% credible interval)

Table S16B. Papers estimating the effect of non-pharmaceutical interventions on COVID-19 transmission and/or cases in the Indian subcontinent

Citation	Reference number	Data	Location	Date range	Interventions	Estimate	Metric
Abaluck et al. (2021)<br>Science<br>doi:<br>10.1126/science.abi9069	(*112*)	Primary data collection	Bangladesh (rural)	November 2020 through April 2021	Mask promotion intervention (free masks, information on importance of mask wearing, role modeling by community leaders, in-person reminders for 8 weeks)	11% (7%, 17%)<br><br>10% (1%, 18%)	Relative reduction in COVID-19 symptoms<br><br>Relative reduction in symptomatic seropositivity

Table S16C. Papers characterizing impact of non-pharmaceutical interventions on COVID-19 transmission

Citation	Reference number	Data	Location	Date range	Conclusions	Type
Zou et al. (2022)<br>IJID<br>doi: 10.1016/j.ijid.2021.11.024	(*113*)	COVID-19 epidemic data from the Australian Department of Health	Australia	January 25, 2020 through March 12, 2021	A wildtype-dominant epidemic could be contained through combinations of social distancing and face mask use interventions; Alpha-dominant epidemic would require stronger measures that start earlier; Delta-dominant epidemic could not be contained through public health interventions unless vaccination coverage is high	Mathematical modeling study
Mendez-Brito (2021)<br>Journal of Infection<br>doi: 10.1016/j.jinf.2021.06.018	(*66*)	Meta-analysis of thirty-four studies	Multiple	Varies	School closing, followed by workplace closing, business and venue closing and public event bans were the most effective interventions in controlling the spread of COVID-19; early response is important and effective at reducing COVID-19 case and death counts	Meta-analysis
Bajiya et al. (2020)<br>Chaos<br>doi: 10.1063/5.0021353	(*114*)	World Health Organization and Worldometer	India	May 1, 2020 through June 25, 2020 (predicted through July 31, 2020)	Implementation of almost complete isolation and 33% increment in contact-tracing would result in approximately 54% reduction in cumulative cases from June 26, 2020 through July 31, 2020	Mathematical modeling study

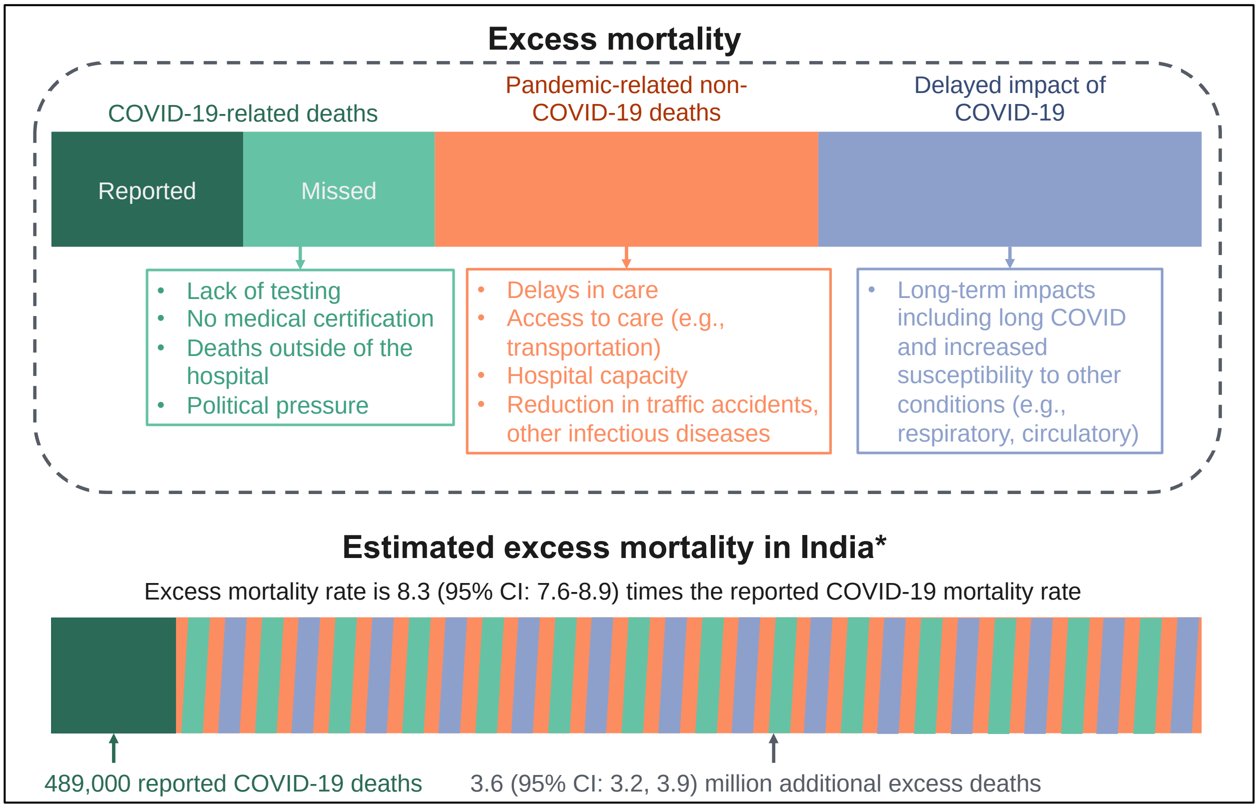

Figure S16. A schematic representing major components captured by the excess mortality metric and excess mortality in India through the COVID-19 pandemic as estimated by the Wang et al. (doi: 10.1016/S0140-6736(21)02796-3) (*24*).

REFERENCES AND NOTES

1. Population, total - India: https://data.worldbank.org/indicator/SP.POP.TOTL?locations=IN.

2. M. A. Andrews, B. Areekal, K. R. Rajesh, J. Krishnan, R. Suryakala, B. Krishnan, C. P. Muraly, P. V. Santhosh, First confirmed case of COVID-19 infection in India: A case report. *Indian J. Med. Res.* **151**, 490–492 (2020).

3. COVID-19 India Timeline, *The Wire*; https://thewire.in/covid-19-india-timeline.

4. WHO Director-General's opening remarks at the media briefing on COVID-19 - 11 March 2020: www.who.int/director-general/speeches/detail/who-director-general-s-opening-remarks-at-the-media-briefing-on-covid-19---11-march-2020.

5. D. Ray, M. Salvatore, R. Bhattacharyya, L. Wang, J. Du, S. Mohammed, S. Purkayastha, A. Halder, A. Rix, D. Barker, M. Kleinsasser, Y. Zhou, D. Bose, P. Song, M. Banerjee, V. Baladandayuthapani, P. Ghosh, B. Mukherjee, Predictions, role of interventions and effects of a historic national lockdown in India's response to the the COVID-19 pandemic: Data science call to arms. *Harv. Data Sci. Rev.* 10.1162/99608f92.60e08ed5 (2020).

6. T. N. Service, Centre extends nationwide lockdown till May 31, new guidelines issued, *Trib. News Serv.*; www.tribuneindia.com/news/nation/centre-extends-nationwide-lockdown-till-may-31-new-guidelines-issued-86042.

7. J. Gettleman, K. Schultz, Modi Orders 3-Week Total Lockdown for All 1.3 Billion Indians, *N. Y. Times*, 2020: www.nytimes.com/2020/03/24/world/asia/india-coronavirus-lockdown.html.

8. U. Bhaskar, India to remain closed till 3 May, economy to open up gradually in lockdown 2.0, *mint*, 2020: www.livemint.com/news/india/pm-modi-announces-extension-of-lockdown-till-3-may-11586839412073.html.

9. G. R. Babu, D. Ray, R. Bhaduri, A. Halder, R. Kundu, G. I. Menon, B. Mukherjee, COVID-19 pandemic in India: Through the lens of modeling. *Glob. Health Sci. Pract.* **9**, 220–228 (2021).

10. "Unlock1": Malls, Restaurants, Places Of Worship To Reopen June 8, *NDTV.com*; www.ndtv.com/india-news/lockdown-extended-till-june-30-malls-restaurants-can-reopen-from-june-8-except-in-containment-zones-2237910.

11. M. Salvatore, D. Basu, D. Ray, M. Kleinsasser, S. Purkayastha, R. Bhattacharyya, B. Mukherjee, Comprehensive public health evaluation of lockdown as a non-pharmaceutical intervention on COVID-19 spread in India: National trends masking state-level variations. *BMJ Open* **10**, e041778 (2020).

12. J. Jesline, J. Romate, E. Rajkumar, A. J. George, The plight of migrants during COVID-19 and the impact of circular migration in India: A systematic review. *Humanit. Soc. Sci. Commun.* **8**, 1–12 (2021).

13. A. K. Enugu, J. Thakkar, S. Ghosh, R. Pollard, A. M. McFall, C. K. Vasudevan, E. Thamburaj, A. Singh, S. H. Mehta, S. S. Solomon, COVID-19 impact on index testing services in 5 high hiv prevalence Indian districts, *CROI Conf.*; www.croiconference.org/abstract/covid-19-impact-on-index-testing-services-in-5-high-hiv-prevalence-indian-districts/.

14. R. Shrinivasan, S. Rane, M. Pai, India's syndemic of tuberculosis and COVID-19. *BMJ Glob. Health* **5**, e003979 (2020).

15. UNICEF, COVID-19 pandemic leads to major backsliding on childhood vaccinations, new WHO, UNICEF data shows; www.unicef.org/press-releases/covid-19-pandemic-leads-major-backsliding-childhood-vaccinations-new-who-unicef-data.

16. COVID-19 India, COVID-19 Tracker Updates for India for State-wise and District-wise data, 2020; www.covid19india.org/.

17. M. V. Murhekar, T. Bhatnagar, J. W. V. Thangaraj, V. Saravanakumar, M. S. Kumar, S. Selvaraju, K. Rade, C. P. G. Kumar, R. Sabarinathan, A. Turuk, S. Asthana, R. Balachandar, S. D. Bangar, A. K. Bansal, V. Chopra, D. Das, A. K. Deb, K. R. Devi, V. Dhikav, G. R. Dwivedi, S. M. S. Khan, M. S. Kumar, A. Laxmaiah, M. Madhukar, A. Mahapatra, C. Rangaraju, J. Turuk, R. Yadav, R. Andhalkar, K. Arunraj, D. K. Bharadwaj, P. Bharti, D. Bhattacharya, J. Bhat, A. S. Chahal, D. Chakraborty, A. Chaudhury, H. Deval, S. Dhatrak, R. Dayal, D. Elantamilan, P. Giridharan, I. Haq, R. K. Hudda, B. Jagjeevan, A. Kalliath, S. Kanungo, N. N. Krishnan, J. S. Kshatri, A. Kumar, N. Kumar, V. G. V. Kumar, G. G. J. N. Lakshmi, G. Mehta, N. K. Mishra, A. Mitra, K. Nagbhushanam, A. Nimmathota, A. R. Nirmala, A. K. Pandey, G. V. Prasad, M. A. Qurieshi, S. D. Reddy, A. Robinson, S. Sahay, R. Saxena, K. Sekar, V. K. Shukla, H. B. Singh, P. K. Singh, P. Singh, R. Singh, N. Srinivasan, D. S. Varma, A. Viramgami, V. C. Wilson, S. Yadav, S. Yadav, K. Zaman, A. Chakrabarti, A. Das, R. S. Dhaliwal, S. Dutta, R. Kant, A. M. Khan, K. Narain, S. Narasimhaiah, C. Padmapriyadarshini, K. Pandey, S. Pati, S. Patil, H. Rajkumar, T. Ramarao, Y. K. Sharma, S. Singh, S. Panda, D. C. S. Reddy, B. Bhargava; ICMR Serosurveillance Group, SARS-CoV-2 seroprevalence among the general population and healthcare workers in India, December 2020-January 2021. *Int. J. Infect. Dis. IJID Off. Publ. Int. Soc. Infect. Dis.* **108**, 145–155 (2021).

18. Coronavirus | World's largest vaccination programme begins in India on January 16, *The Hindu* (2021); www.thehindu.com/news/national/coronavirus-worlds-largest-vaccination-programme-begins-in-india-on-january-16/article33582069.ece.

19. A. Deshpande, Maharashtra imposes ‘mini lockdown’ amid rising coronavirus cases, *The Hindu* (2021); www.thehindu.com/news/national/coronavirus-curfew-in-maharashtra-for-15-days-from-april-14-says-uddhav-thackeray/article34313068.ece.

20. K. Kuppalli, P. Gala, K. Cherabuddi, S. P. Kalantri, M. Mohanan, B. Mukherjee, L. Pinto, M. Prakash, C. S. Pramesh, S. Rathi, N. P. Pai, G. Yamey, M. Pai, India’s COVID-19 crisis: A call for international action. *The Lancet* **397**, 2132–2135 (2021).

21. The RECOVERY Dexamethasone in hospitalized patients with Covid-19. *N. Engl. J. Med.* **384**, 693–704 (2021).

22. V. Pandey, S. Nazmi, Covid-19 in India: Why second coronavirus wave is devastating, *BBC News*, 2021; www.bbc.com/news/world-asia-india-56811315.

23. L. Zimmermann, S. Bhattacharya, S. Purkayastha, R. Kundu, R. Bhaduri, P. Ghosh, B. Mukherjee, SARS-CoV-2 infection fatality rates in India: Systematic review, meta-analysis and model-based estimation. *Stud. Microecon.*, 23210222211054324 (2021).

24. H. Wang, K. R. Paulson, S. A. Pease, S. Watson, H. Comfort, P. Zheng, A. Y. Aravkin, C. Bisignano, R. M. Barber, T. Alam, J. E. Fuller, E. A. May, D. P. Jones, M. E. Frisch, C. Abbafati, C. Adolph, A. Allorant, J. O. Amlag, B. Bang-Jensen, G. J. Bertolacci, S. S. Bloom, A. Carter, E. Castro, S. Chakrabarti, J. Chattopadhyay, R. M. Cogen, J. K. Collins, K. Cooperrider, X. Dai, W. J. Dangel, F. Daoud, C. Dapper, A. Deen, B. B. Duncan, M. Erickson, S. B. Ewald, T. Fedosseeva, A. J. Ferrari, J. J. Frostad, N. Fullman, J. Gallagher, A. Gamkrelidze, G. Guo, J. He, M. Helak, N. J. Henry, E. N. Hulland, B. M. Huntley, M. Kereselidze, A. Lazzar-Atwood, K. E. LeGrand, A. Lindstrom, E. Linebarger, P. A. Lotufo, R. Lozano, B. Magistro, D. C. Malta, J. Månsson, A. M. M. Herrera, F. Marinho, A. H. Mirkuzie, A. T. Misganaw, L. Monasta, P. Naik, S. Nomura, E. G. O’Brien, J. K. O’Halloran, L. T. Olana, S. M. Ostroff, L. Penberthy, R. C. Reiner Jr., G. Reinke, A. L. P. Ribeiro, D. F. Santomauro, M. I. Schmidt, D. H. Shaw, B. S. Sheena, A. Sholokhov, N. Skhvitaridze, R. J. D. Sorensen, E. E. Spurlock, R. Syailendrawati, R. Topor-Madry, C. E. Troeger, R. Walcott, A. Walker, C. S. Wiysonge, N. A. Worku, B. Zigler, D. M. Pigott, M. Naghavi, A. H. Mokdad, S. S. Lim, S. I. Hay, E. Gakidou, C. J. L. Murray, Estimating excess mortality due to the COVID-19 pandemic: A systematic analysis of COVID-19-related mortality, 2020–21. *Lancet*, S0140673621027963 (2022).

25. P. Jha, Y. Deshmukh, C. Tumbe, W. Suraweera, A. Bhowmick, S. Sharma, P. Novosad, S. H. Fu, L. Newcombe, H. Gelband, P. Brown, COVID mortality in India: National survey data and health facility deaths. *Science* **375**, 667–671 (2022).

26. M. S. Dhar, R. Marwal, V. Radhakrishnan, K. Ponnusamy, B. Jolly, R. C. Bhoyar, S. Fatihi, M. Datta, P. Singh, U. Sharma, R. Ujjainia, S. Naushin, N. Bhateja, M. K. Divakar, V. Sardana, M. K. Singh, M. Imran, V. Senthivel, R. Maurya, N. Jha, P. Mehta, M. Rophina, V.

Arvinden, U. Chaudhary, L. Thukral, R. Pandey, D. Dash, M. Faruq, H. Lall, H. Gogia, P. Madan, S. Kulkarni, H. Chauhan, S. Sengupta, S. Kabra; The Indian SARS-CoV-2 Genomics Consortium (INSACOG), S. K. Singh, A. Agrawal, P. Rakshit, "Genomic characterization and epidemiology of an emerging SARS-CoV-2 variant in Delhi, India" (medRxiv, 2021).

27. R. Sarkar, R. Saha, P. Mallick, R. Sharma, A. Kaur, S. Dutta, M. Chawla-Sarkar, "Emergence of a new SARS-CoV-2 variant from GR clade with a novel S glycoprotein mutation V1230L in West Bengal, India" (medRxiv, 2021).

28. World Health Organization, Tracking SARS-CoV-2 variants; www.who.int/en/activities/tracking-SARS-CoV-2-variants/.

29. Genome sequencing by INSACOG shows variants of concern and a Novel variant in India; pib.gov.in/Pressreleaseshare.aspx?PRID=1707177.

30. S. Cherian, V. Potdar, S. Jadhav, P. Yadav, N. Gupta, M. Das, P. Rakshit, S. Singh, P. Abraham, S. Panda, N. Team, SARS-CoV-2 Spike mutations, L452R, T478K, E484Q and P681R, in the second wave of COVID-19 in Maharashtra, India. *Microorganisms*. **9**, 1542 (2021).

31. Maharashtra: Double mutant found in 61% samples tested, *Indian Express*, 2021; https://indianexpress.com/article/cities/mumbai/maharashtra-double-mutant-found-in-61-samples-tested-7272524/.

32. World Health Organization, Weekly epidemiological update on COVID-19 - 11 May 2021 (2021); www.who.int/publications/m/item/weekly-epidemiological-update-on-covid-19---11-may-2021.

33. C. Pattabiraman, P. Prasad, A. K. George, D. Sreenivas, R. Rasheed, N. V. K. Reddy, A. Desai, R. Vasanthapuram, Importation, circulation, and emergence of variants of SARS-CoV-2 in the South Indian state of Karnataka. *Wellcome Open Res.* **6**, 110 (2021).

34. J. Koshy, Coronavirus | New virus lineage found in West Bengal, *The Hindu* (2021); www.thehindu.com/sci-tech/health/new-coronavirus-variant-found-in-west-bengal/article34373083.ece.

35. 81 per cent of Punjab COVID-19 samples sent for genome sequencing show new UK variant, *New Indian Express*, 2021; www.newindianexpress.com/nation/2021/mar/23/81-per-cent-of-punjab-covid-19-samples-sent-for-genome-sequencing-show-new-uk-variant-2280394.html.

36. F. Campbell, B. Archer, H. Laurenson-Schafer, Y. Jinnai, F. Konings, N. Batra, B. Pavlin, K. Vandemaele, M. D. Van Kerkhove, T. Jombart, O. Morgan, O. le Polain de Waroux, Increased transmissibility and global spread of SARS-CoV-2 variants of concern as at June 2021. *Eurosurveill*. **26**, 2100509 (2021).

37. L. Wang, Y. Zhou, J. He, B. Zhu, F. Wang, L. Tang, M. Kleinsasser, D. Barker, M. C. Eisenberg, P. X. K. Song, An epidemiological forecast model and software assessing interventions on the COVID-19 epidemic in China. *J. Data Sci.* **18**, 409–432 (2021).

38. J. Wangping, H. Ke, S. Yang, C. Wenzhe, W. Shengshu, Y. Shanshan, W. Jianwei, K. Fuyin, T. Penggang, L. Jing, L. Miao, H. Yao, Extended SIR prediction of the epidemics trend of covid-19 in italy and compared with Hunan, China. *Front. Med.* **7**, 169 (2020).

39. A. Cori, S. Cauchemez, N. M. Ferguson, C. Fraser, E. Dahlqwist, P. A. Demarsh, T. Jombart, Z. N. Kamvar, J. Lessler, S. Li, J. A. Polonsky, J. Stockwin, R. Thompson, R. van Gaalen, *EpiEstim: Estimate Time Varying Reproduction Numbers from Epidemic Curves* (2021); https://CRAN.R-project.org/package=EpiEstim.

40. A. Cori, N. M. Ferguson, C. Fraser, S. Cauchemez, A new framework and software to estimate time-varying reproduction numbers during epidemics. *Am. J. Epidemiol.* **178**, 1505–1512 (2013).

41. Y. Liu, J. Rocklöv, The reproductive number of the Delta variant of SARS-CoV-2 is far higher compared to the ancestral SARS-CoV-2 virus. *J. Travel Med.* **28**, taab124 (2021).

42. R. Bhaduri, R. Kundu, S. Purkayastha, M. Kleinsasser, L. J. Beesley, B. Mukherjee, J. Datta, Extending the susceptible-exposed-infected-removed (SEIR) model to handle the false negative rate and symptom-based administration of COVID-19 diagnostic tests: SEIR-fansy. *Stat. Med.*, **41** 2317–2337 (2022).

43. A. Ghosh, 4th sero survey finds 2 of 3 Indians with Covid antibodies, but still avoid crowds, ICMR warns, *ThePrint* (2021); https://theprint.in/health/4th-sero-survey-finds-2-of-3-indians-with-covid-antibodies-but-still-avoid-crowds-icmr-warns/699600/.

44. Y. Deshmukh, W. Suraweera, C. Tumbe, A. Bhowmick, S. Sharma, P. Novosad, S. H. Fu, L. Newcombe, H. Gelband, P. Brown, P. Jha, “Excess mortality in India from June 2020 to June 2021 during the COVID pandemic: Death registration, health facility deaths, and survey data” (medRxiv, 2021).

45. G. Kapoor, A. Sriram, J. Joshi, A. Nandi, R. Laxminarayan, COVID-19 in India: State-Wise Estimates of Current Hospital Beds, ICU Beds, and Ventilators, *CDDEP*, 2020; https://cddep.org/publications/covid-19-in-india-state-wise-estimates-of-current-hospital-beds-icu-beds-and-ventilators/.

46. K. Tseng, I. Frost, G. Kapoor, A. Sriram, A. Nandi, R. Laxminarayan, COVID-19 in India: State-level estimates of hospitalization needs, *CDDEP*; https://cddep.org/publications/covid-19-in-india-state-level-estimates-of-hospitalization-needs/.

47. A. Sibal, K. H. Prasad, S. Reddy, P. M. Doraiswamy, Apollo Hospitals and Project Kavach: Insights from How India's Largest Private Health System Is Handling Covid-19, *NEJM Catalyst*, 2021; https://catalyst.nejm.org/doi/full/10.1056/CAT.20.0677.

48. World Health Organization, Considerations for implementing and adjusting public health and social measures in the context of COVID-19 (2021); www.who.int/publications-detail-redirect/considerations-in-adjusting-public-health-and-social-measures-in-the-context-of-covid-19-interim-guidance.

49. Ontario Ministry of Health, "COVID-19 Response Framework: Keeping Ontario Safe and Open—Lockdown Measures" (2020), p. 35.

50. Scottish Government, Coronavirus (COVID-19): Scotland's route map through and out of the crisis (2020); www.gov.scot/publications/coronavirus-covid-19-framework-decision-making-scotlands-route-map-through-out-crisis/.

51. Singapore Ministry of Health, "MOH Pandemic Readiness and Response Plan for Influenza and Other Acute Respiratory Diseases" (2014); www.moh.gov.sg/docs/librariesprovider5/diseases-updates/interim-pandemic-plan-public-ver-_april-2014.pdf.

52. Johns Hopkins Coronavirus Resource Center, India - COVID-19 Overview; https://coronavirus.jhu.edu/region/india.

53. K. A. Twohig, T. Nyberg, A. Zaidi, S. Thelwall, M. A. Sinnathamby, S. Aliabadi, S. R. Seaman, R. J. Harris, R. Hope, J. Lopez-Bernal, E. Gallagher, A. Charlett, D. De Angelis, A. M. Presanis, G. Dabrera; COVID-19 Genomics UK (COG-UK) consortium, Hospital admission and emergency care attendance risk for SARS-CoV-2 delta (B.1.617.2) compared with alpha (B.1.1.7) variants of concern: A cohort study. *Lancet Infect. Dis.* **22**, 35–42 (2022).

54. R. Viana, S. Moyo, D. G. Amoako, H. Tegally, C. Scheepers, C. L. Althaus, U. J. Anyaneji, P. A. Bester, M. F. Boni, M. Chand, W. T. Choga, R. Colquhoun, M. Davids, K. Deforche, D. Doolabh, L. du Plessis, S. Engelbrecht, J. Everatt, J. Giandhari, M. Giovanetti, D. Hardie, V. Hill, N.-Y. Hsiao, A. Iranzadeh, A. Ismail, C. Joseph, R. Joseph, L. Koopile, S. L. K. Pond, M. U. G. Kraemer, L. Kuate-Lere, O. Laguda-Akingba, O. Lesetedi-Mafoko, R. J. Lessells, S. Lockman, A. G. Lucaci, A. Maharaj, B. Mahlangu, T. Maponga, K. Mahlakwane, Z. Makatini, G. Marais, D. Maruapula, K. Masupu, M. Matshaba, S. Mayaphi, N. Mbhele, M. B. Mbulawa, A. Mendes, K. Mlisana, A. Mnguni, T. Mohale, M. Moir, K. Moruisi, M. Mosepele, G. Motsatsi, M. S. Motswaledi, T. Mphoyakgosi, N. Msomi, P. N. Mwangi, Y. Naidoo, N. Ntuli, M. Nyaga, L. Olubayo, S. Pillay, B. Radibe, Y. Ramphal, U. Ramphal, J. E. San, L. Scott, R. Shapiro, L. Singh, P. Smith-Lawrence, W. Stevens, A. Strydom, K. Subramoney, N. Tebeila, D. Tshiabuila, J. Tsui, S. van Wyk, S. Weaver, C. K.

Wibmer, E. Wilkinson, N. Wolter, A. E. Zarebski, B. Zuze, D. Goedhals, W. Preiser, F. Treurnicht, M. Venter, C. Williamson, O. G. Pybus, J. Bhiman, A. Glass, D. P. Martin, A. Rambaut, S. Gaseitsiwe, A. von Gottberg, T. de Oliveira, Rapid epidemic expansion of the SARS-CoV-2 omicron variant in southern Africa. *Nature* **603**, 679–686 (2022).

55. W. Ma, J. Yang, H. Fu, C. Su, C. Yu, Q. Wang, A. T. R. de Vasconcelos, G. A. Bazykin, Y. Bao, M. Li, Genomic perspectives on the emerging SARS-CoV-2 omicron variant.*Genom. Proteom. Bioinform.*, S1672-0229(22)00002-X (2022).

56. H. de Puig, R. A. Lee, D. Najjar, X. Tan, L. R. Soekensen, N. M. Angenent-Mari, N. M. Donghia, N. E. Weckman, A. Ory, C. F. Ng, P. Q. Nguyen, A. S. Mao, T. C. Ferrante, G. Lansberry, H. Sallum, J. Niemi, J. J. Collins, Minimally instrumented SHERLOCK (miSHERLOCK) for CRISPR-based point-of-care diagnosis of SARS-CoV-2 and emerging variants. *Sci. Adv.* **7**, eabh2944 (2021).

57. M. Hemalatha, U. Kiran, S. K. Kuncha, H. Kopperi, C. G. Gokulan, S. V. Mohan, R. K. Mishra, Surveillance of SARS-CoV-2 spread using wastewater-based epidemiology: Comprehensive study. *Sci. Total Environ.* **768**, 144704 (2021).

58. S. Priyadarshini, India's sewage surveillance for SARS-CoV-2 going down the drain, *Nature* (2021).

59. Indian Biological Data Centre, INSACOG Covid-19 Data. Indian Nucleotide Data Arch. – Control, *Access INDA-CA*; http://inda.rcb.ac.in:8080/insacog/indexpage.

60. S. Purkayastha, R. Kundu, R. Bhaduri, D. Barker, M. Kleinsasser, D. Ray, B. Mukherjee, Estimating the wave 1 and wave 2 infection fatality rates from SARS-CoV-2 in India. *BMC. Res. Notes* **14**, 262 (2021).

61. R. Subbaraman, L. Ganapathi, B. Mukherjee, D. E. Bloom, S. S. Solomon, Humane shelter at home: A call to reimagine a core pandemic intervention. *BMJ Glob. Health* **6**, e006614 (2021).

62. G. Meyerowitz-Katz, S. Bhatt, O. Ratmann, J. M. Brauner, S. Flaxman, S. Mishra, M. Sharma, S. Mindermann, V. Bradley, M. Vollmer, L. Merone, G. Yamey, Is the cure really worse than the disease? The health impacts of lockdowns during COVID-19. *BMJ Glob. Health* **6**, e006653 (2021).

63. India Today Web Desk New Delhi, Full list of Red, Yellow, Green Zone districts for Lockdown 3.0, *India Today*, 2020; www.indiatoday.in/india/story/red-orange-green-zones-full-current-update-list-districts-states-india-coronavirus-1673358-2020-05-01.

64. Y. Bo, C. Guo, C. Lin, Y. Zeng, H. B. Li, Y. Zhang, M. S. Hossain, J. W. M. Chan, D. W. Yeung, K. O. Kwok, S. Y. S. Wong, A. K. H. Lau, X. Q. Lao, Effectiveness of non-

pharmaceutical interventions on COVID-19 transmission in 190 countries from 23 January to 13 April 2020. *Int. J. Infect. Dis.* **102**, 247–253 (2021).

65. S. Pei, S. Kandula, J. Shaman, Differential effects of intervention timing on COVID-19 spread in the United States. *Sci. Adv.* **6**, eabd6370 (2020).

66. A. Mendez-Brito, C. El Bcheraoui, F. Pozo-Martin, Systematic review of empirical studies comparing the effectiveness of non-pharmaceutical interventions against COVID-19. *J. Infect.* **83**, 281–293 (2021).

67. Institute for Health Metrics and Evaluation, Causes of Death in India, 2009–2019, *GBD Comp.*; http://vizhub.healthdata.org/gbd-compare.

68. A. I. Middya, S. Roy, Geographically varying relationships of COVID-19 mortality with different factors in India. *Sci. Rep.* **11**, 7890 (2021).

69. N. Arinaminpathy, J. Das, T. H. McCormick, P. Mukhopadhyay, N. Sircar, Quantifying heterogeneity in SARS-CoV-2 transmission during the lockdown in India. *Epidemics* **36**, 100477 (2021).

70. H. Ritchie, E. Mathieu, L. Rodés-Guirao, C. Appel, C. Giattino, E. Ortiz-Ospina, J. Hasell, B. Macdonald, D. Beltekian, M. Roser, Coronavirus (COVID-19) Vaccinations (Our World in Data, 2020); https://ourworldindata.org/covid-vaccinations.

71. S. K C, Covid-19 vaccination drive: Over 10 million administered doses on Tuesday, 500 million given first dose so far, *Hindustan Times*, 2021; www.hindustantimes.com/india-news/covid19-vaccination-drive-over-10-million-administered-doses-on-tuesday-500-million-given-first-dose-so-far-101630417853792.html.

72. Microsoft, Microsoft Bing COVID-19 Tracker; www.bing.com/covid?ref=share.

73. S. S. A. Karim, Q. A. Karim, Omicron SARS-CoV-2 variant: A new chapter in the COVID-19 pandemic. *Lancet* **398**, 2126–2128 (2021).

74. Unique Identification Authority of India, “State/UT wise Aadhar Saturation” (2020); https://uidai.gov.in/images/state-wise-aadhaar-saturation.pdf.

75. M. Salvatore, S. Purkayastha, R. Kundu, *umich-cphds/covid_india_wave2:* (Zenodo, 2022); https://zenodo.org/record/6514992.

76. Y. Zhou, L. Wang, L. Zhang, L. Shi, K. Yang, J. He, B. Zhao, W. Overton, S. Purkayastha, P. Song, A spatiotemporal epidemiological prediction model to inform county-level COVID-19 risk in the United States. *Harv. Data Sci. Rev.*, 10.1162/99608f92.79e1f45e (2020).

77. N. G. Davies, S. Abbott, R. C. Barnard, C. I. Jarvis, A. J. Kucharski, J. D. Munday, C. A. B. Pearson, T. W. Russell, D. C. Tully, A. D. Washburne, T. Wenseleers, A. Gimma, W. Waites, K. L. M. Wong, K. van Zandvoort, J. D. Silverman; CMMID COVID-19 Working Group, COVID-19 Genomics UK (COG-UK) Consortium, K. Diaz-Ordaz, R. Keogh, R. M. Eggo, S. Funk, M. Jit, K. E. Atkins, W. J. Edmunds, Estimated transmissibility and impact of SARS-CoV-2 lineage B.1.1.7 in England. *Science* **372**, eabg3055 (2021).

78. CSIR Institute of Genomics & Integrative Biology, COVID-19 Genomic Surveillance; https://clingen.igib.res.in/covid19genomes/.

79. C. H. Hansen, D. Michlmayr, S. M. Gubbels, K. Mølbak, S. Ethelberg, Assessment of protection against reinfection with SARS-CoV-2 among 4 million PCR-tested individuals in Denmark in 2020: A population-level observational study. *The Lancet* **397**, 1204–1212 (2021).

80. *R package SEIRfansy* (Department of Biostatistics at the University of Michigan, 2021); https://github.com/umich-biostatistics/SEIRfansy.

81. M. Robinson, N. I. Stilianakis, A model for the emergence of drug resistance in the presence of asymptomatic infections. *Math. Biosci.* **243**, 163–177 (2013).

82. T. Mkhatshwa, A. Mummert, Modeling super-spreading events for infectious diseases: Case study SARS. arXiv:1007.0908 [q-bio.PE] (2010); http://arxiv.org/abs/1007.0908.

83. N. Chen, M. Zhou, X. Dong, J. Qu, F. Gong, Y. Han, Y. Qiu, J. Wang, Y. Liu, Y. Wei, J. Xia, T. Yu, X. Zhang, L. Zhang, Epidemiological and clinical characteristics of 99 cases of 2019 novel coronavirus pneumonia in Wuhan, China: A descriptive study. *Lancet* **395**, 507–513 (2020).

84. N. R. Faria, T. A. Mellan, C. Whittaker, I. M. Claro, D. da S. Jesus, S. Mishra, M. A. E. Crispim, F. C. S. Sales, I. Hawryluk, J. T. McCrone, R. J. G. Hulswit, L. A. M. Franco, M. S. Ramundo, J. G. de Jesus, P. S. Andrade, T. M. Coletti, G. M. Ferreira, C. A. M. Silva, E. R. Manuli, R. H. M. Pereira, P. S. Peixoto, M. U. G. Kraemer, N. Gaburo, C. da. C. Camilo, H. Hoeltgebaum, W. M. Souza, E. C. Rocha, L. M. de Souza, M. C. de Pinho, L. J. T. Araujo, F. S. V. Malta, A. B. de Lima, J. do P. Silva, D. A. G. Zauli, A. C. de S. Ferreira, R. P. Schnekenberg, D. J. Laydon, P. G. T. Walker, H. M. Schlüter, A. L. P. dos Santos, M. S. Vidal, V. S. D. Caro, R. M. F. Filho, H. M. dos Santos, R. S. Aguiar, J. L. Proença-Modena, B. Nelson, J. A. Hay, M. Monod, X. Miscouridou, H. Coupland, R. Sonabend, M. Vollmer, A. Gandy, C. A. Prete, V. H. Nascimento, M. A. Suchard, T. A. Bowden, S. L. K. Pond, C.-H. Wu, O. Ratmann, N. M. Ferguson, C. Dye, N. J. Loman, P. Lemey, A. Rambaut, N. A. Fraiji, M. do P. S. S. Carvalho, O. G. Pybus, S. Flaxman, S. Bhatt, E. C. Sabino, Genomics and epidemiology of the P.1 SARS-CoV-2 lineage in Manaus, Brazil. *Science* **372**, 815–821 (2021).

85. S. Das, Prediction of COVID-19 disease progression in India: Under the effect of national lockdown. arXiv:2004.03147 [q-bio.PE] (2020); http://arxiv.org/abs/2004.03147.

86. S. Deb, M. Majumdar, A time series method to analyze incidence pattern and estimate reproduction number of COVID-19. arXiv:2003.10655 [stat.AP] (2020); http://arxiv.org/abs/2003.10655.

87. R. Ranjan, "Predictions for COVID-19 outbreak in India using epidemiological models" (preprint, Epidemiology, 2020).

88. T. Sardar, S. S. Nadim, S. Rana, J. Chattopadhyay, Assessment of lockdown effect in some states and overall India: A predictive mathematical study on COVID-19 outbreak. *Chaos Solitons Fractals* **139**, 110078 (2020).

89. R. Singh, R. Adhikari, Age-structured impact of social distancing on the COVID-19 epidemic in India. arXiv:2003.12055 [q-bio.PE] (2020); http://arxiv.org/abs/2003.12055.

90. Q. Li, X. Guan, P. Wu, X. Wang, L. Zhou, Y. Tong, R. Ren, K. S. M. Leung, E. H. Y. Lau, J. Y. Wong, X. Xing, N. Xiang, Y. Wu, C. Li, Q. Chen, D. Li, T. Liu, J. Zhao, M. Liu, W. Tu, C. Chen, L. Jin, R. Yang, Q. Wang, S. Zhou, R. Wang, H. Liu, Y. Luo, Y. Liu, G. Shao, H. Li, Z. Tao, Y. Yang, Z. Deng, B. Liu, Z. Ma, Y. Zhang, G. Shi, T. T. Y. Lam, J. T. Wu, G. F. Gao, B. J. Cowling, B. Yang, G. M. Leung, Z. Feng, Early transmission dynamics in Wuhan, China, of novel coronavirus–infected pneumonia. *N. Engl. J. Med.* **382**, 1199–1207 (2020).

91. S. Ryu, B. C. Chun, An interim review of the epidemiological characteristics of 2019 novel coronavirus. *Epidemiol. Health*. **42**, e2020006 (2020).

92. M. Murhekar, T. Bhatnagar, S. Selvaraju, K. Rade, V. Saravanakumar, J. Vivian Thangaraj, M. Kumar, N. Shah, R. Sabarinathan, A. Turuk, P. Anand, S. Asthana, R. Balachandar, S. Bangar, A. Bansal, J. Bhat, D. Chakraborty, C. Rangaraju, V. Chopra, D. Das, A. Deb, K. Devi, G. Dwivedi, S. M. S. Khan, I. Haq, M. S. Kumar, A. Laxmaiah, Madhuka, A. Mahapatra, A. Mitra, A. Nirmala, A. Pagdhune, M. Qurieshi, T. Ramarao, S. Sahay, Y. Sharma, M. Shrinivasa, V. Shukla, P. Singh, A. Viramgami, V. Wilson, R. Yadav, C. Girish Kumar, H. Luke, U. Ranganathan, S. Babu, K. Sekar, P. Yadav, G. Sapkal, A. Das, P. Das, S. Dutta, R. Hemalatha, A. Kumar, K. Narain, S. Narasimhaiah, S. Panda, S. Pati, S. Patil, K. Sarkar, S. Singh, R. Kant, S. Tripathy, G. Toteja, G. Babu, S. Kant, J. Muliyil, R. Pandey, S. Sarkar, S. Singh, S. Zodpey, R. Gangakhedkar, D. S. Reddy, B. Bhargava, Prevalence of SARS-CoV-2 infection in India: Findings from the national serosurvey, May-June 2020. *Indian J. Med. Res.* **152**, 48 (2020).

93. M. V. Murhekar, T. Bhatnagar, S. Selvaraju, V. Saravanakumar, J. W. V. Thangaraj, N. Shah, M. S. Kumar, K. Rade, R. Sabarinathan, S. Asthana, R. Balachandar, S. D. Bangar, A. K. Bansal, J. Bhat, V. Chopra, D. Das, A. K. Deb, K. R. Devi, G. R. Dwivedi, S. M. S. Khan,

C. P. G. Kumar, M. S. Kumar, A. Laxmaiah, M. Madhukar, A. Mahapatra, S. S. Mohanty, C. Rangaraju, A. Turuk, D. K. Baradwaj, A. S. Chahal, F. Debnath, I. Haq, A. Kalliath, S. Kanungo, J. S. Kshatri, G. G. J. N. Lakshmi, A. Mitra, A. R. Nirmala, G. V. Prasad, M. A. Qurieshi, S. Sahay, R. K. Sangwan, K. Sekar, V. K. Shukla, P. K. Singh, P. Singh, R. Singh, D. S. Varma, A. Viramgami, S. Panda, D. C. S. Reddy, B. Bhargava, R. Andhalkar, A. Chaudhury, H. Deval, S. Dhatrak, R. R. Gupta, E. Ilayaperumal, B. Jagjeevan, R. C. Jha, K. Kiran, N. N. Krishnan, A. Kumar, V. V. Kumar, K. Nagbhushanam, A. Nimmathota, A. K. Pandey, H. S. Pawar, K. S. Rathore, A. Robinson, H. B. Singh, V. C. Wilson, A. Yadav, R. Yadav, T. Karunakaran, J. Pradhan, T. Sivakumar, A. Jose, K. Kalaiyarasi, S. Dasgupta, R. Anusha, T. Anand, G. R. Babu, H. Chauhan, T. Dikid, R. R. Gangakhedkar, S. Kant, S. Kulkarni, J. P. Muliyil, R. M. Pandey, S. Sarkar, A. Shrivastava, S. K. Singh, S. Zodpey, A. Das, P. Das, S. Dutta, R. Kant, K. Narain, S. Narasimhaiah, S. Pati, S. Patil, H. Rajkumar, T. Ramarao, K. Sarkar, S. Singh, G. S. Toteja, K. Zaman, SARS-CoV-2 antibody seroprevalence in India, August–September, 2020: Findings from the second nationwide household serosurvey. *Lancet Glob. Health* **9**, e257–e266 (2021).

94. S. Goswami, Fourth serosurvey finds 67.6% have antibodies, 40 crore Indians still vulnerable, Hindustan Times (2021); www.hindustantimes.com/india-news/40-cr-indians-don-t-have-covid-anti-bodies-vulnerable-reveals-4th-serosurvey-101626779228818.html.

95. S. A. Lauer, K. H. Grantz, Q. Bi, F. K. Jones, Q. Zheng, H. R. Meredith, A. S. Azman, N. G. Reich, J. Lessler, The incubation period of coronavirus disease 2019 (COVID-19) from publicly reported confirmed cases: Estimation and application. *Ann. Intern. Med.* **172**, 577–582 (2020).

96. R. Verity, L. C. Okell, I. Dorigatti, P. Winskill, C. Whittaker, N. Imai, G. Cuomo-Dannenburg, H. Thompson, P. G. T. Walker, H. Fu, A. Dighe, J. T. Griffin, M. Baguelin, S. Bhatia, A. Boonyasiri, A. Cori, Z. Cucunubá, R. FitzJohn, K. Gaythorpe, W. Green, A. Hamlet, W. Hinsley, D. Laydon, G. Nedjati-Gilani, S. Riley, S. van Elsland, E. Volz, H. Wang, Y. Wang, X. Xi, C. A. Donnelly, A. C. Ghani, N. M. Ferguson, Estimates of the severity of coronavirus disease 2019: A model-based analysis. *Lancet Infect. Dis.* **20**, 669–677 (2020).

97. India Statistics and Maps, *Atlas Big* (1970); www.atlasbig.com/en-us/india.

98. S. Bhaumik, D. Beri, M. Seerat, N. Gudi, Roopani, R. Dhurjati, N. Dumka, J. Tyagi, "Preparedness for crises response to the second wave of COVID-19 in India: Policy brief" (The George Institute for Global Health, India, National Health Systems Resource Centre, 2021).

99. T. C. Bulfone, M. Malekinejad, G. W. Rutherford, N. Razani, Outdoor transmission of SARS-CoV-2 and other respiratory viruses: A systematic review. *J Infect Dis* **223**, 550–561 (2021).

100. N. Razani, M. Malekinejad, G. W. Rutherford, Clarification regarding outdoor transmission of SARS-CoV-2 and other respiratory viruses, a systematic review. *J. Infect Dis* **224**, 925–926 (2021).

101. K. A. Fisher, M. W. Tenforde, L. R. Feldstein, C. J. Lindsell, N. I. Shapiro, D. C. Files, K. W. Gibbs, H. L. Erickson, M. E. Prekker, J. S. Steingrub, M. C. Exline, D. J. Henning, J. G. Wilson, S. M. Brown, I. D. Peltan, T. W. Rice, D. N. Hager, A. A. Ginde, H. K. Talbot, J. D. Casey, C. G. Grijalva, B. Flannery, M. M. Patel, W. H. Self; IVY Network Investigators, CDC COVID-19 Response Team, IVY Network Investigators, K. W. Hart, R. McClellan, H. Tan, A. Baughman; CDC COVID-19 Response Team, N. A. Hennesy, B. Grear, M. Wu, K. Mlynarczyk, L. Marzano, Z. Plata, A. Caplan, S. M. Olson, C. E. Ogokeh, E. R. Smith, S. S. Kim, E. P. Griggs, B. Richards, S. Robinson, K. Kim, A. M. Kassem, C. N. Sciarratta, P. L. Marcet, Community and close contact exposures associated with COVID-19 among symptomatic adults ≥18 years in 11 outpatient health care facilities — United States, July 2020. *MMWR Morb. Mortal. Wkly Rep.* **69**, 1258–1264 (2020).

102. What is a Lockdown? *Bus. Stand. India*; www.business-standard.com/about/what-is-lockdown#collapse.

103. Ministry of Home Affairs, "Annexure to Ministry of Home Affairs Order No .40-3/2020-D dated ()24.03.2020" (40-3/2020-D, 2020); www.mha.gov.in/sites/default/files/Guidelines.pdf.

104. L. Mathew, A. Ghosh, PM Modi declares lockdown: 'Crossing your home's lakshmanrekha will invite COVID-19 inside,' *Indian Express*, 2020; https://indianexpress.com/article/india/pm-modi-declares-lockdown-crossing-your-homes-lakshmanrekha-will-invite-covid-19-inside-6330017/.

105. Covid-19 India timeline: Looking back at pandemic-induced lockdown and how the country is coping with the crisis, *Indian Express*, 2021; https://indianexpress.com/article/india/covid-19-india-timeline-looking-back-at-pandemic-induced-lockdown-7241583/.

106. Maharashtra brings back Covid-19 e-pass for inter-state, inter-district travel: Here's how to apply, *Hindustan Times*, 2021; www.hindustantimes.com/cities/mumbai-news/maharashtra-brings-back-covid-19-e-pass-for-inter-state-inter-district-travel-here-s-how-to-apply-101619161303652.html.

107. N. Banholzer, E. van Weenen, A. Lison, A. Cenedese, A. Seeliger, B. Kratzwald, D. Tschernutter, J. P. Salles, P. Bottrighi, S. Lehtinen, S. Feuerriegel, W. Vach, Estimating the effects of non-pharmaceutical interventions on the number of new infections with COVID-19 during the first epidemic wave. *PLOS ONE* **16**, e0252827 (2021).

108. J. M. Brauner, S. Mindermann, M. Sharma, D. Johnston, J. Salvatier, T. Gavenčiak, A. B. Stephenson, G. Leech, G. Altman, V. Mikulik, A. J. Norman, J. T. Monrad, T. Besiroglu, H. Ge, M. A. Hartwick, Y. W. Teh, L. Chindelevitch, Y. Gal, J. Kulveit, Inferring the effectiveness of government interventions against COVID-19. *Science* **371**, eabd9338 (2021).

109. S. Talic, S. Shah, H. Wild, D. Gasevic, A. Maharaj, Z. Ademi, X. Li, W. Xu, I. Mesa-Eguiagaray, J. Rostron, E. Theodoratou, X. Zhang, A. Motee, D. Liew, D. Ilic, Effectiveness of public health measures in reducing the incidence of covid-19, SARS-CoV-2 transmission, and covid-19 mortality: Systematic review and meta-analysis. *BMJ* **375**, e068302 (2021).

110. V. Barros, I. Manes, V. Akinwande, C. Cintas, O. Bar-Shira, M. Ozery-Flato, Y. Shimoni, M. Rosen-Zvi, A causal inference approach for estimating effects of non-pharmaceutical interventions during Covid-19 pandemic (2022), p. 2022.02.28.22271671.

111. S. Flaxman, S. Mishra, A. Gandy, H. J. T. Unwin, T. A. Mellan, H. Coupland, C. Whittaker, H. Zhu, T. Berah, J. W. Eaton, M. Monod; Imperial College COVID-19 Response Team, P. N. Perez-Guzman, N. Schmit, L. Cilloni, K. E. C. Ainslie, M. Baguelin, A. Boonyasiri, O. Boyd, L. Cattarino, L. V. Cooper, Z. Cucunubá, G. Cuomo-Dannenburg, A. Dighe, B. Djaafara, I. Dorigatti, S. L. van Elsland, R. G. FitzJohn, K. A. M. Gaythorpe, L. Geidelberg, N. C. Grassly, W. D. Green, T. Hallett, A. Hamlet, W. Hinsley, B. Jeffrey, E. Knock, D. J. Laydon, G. Nedjati-Gilani, P. Nouvellet, K. V. Parag, I. Siveroni, H. A. Thompson, R. Verity, E. Volz, C. E. Walters, H. Wang, Y. Wang, O. J. Watson, P. Winskill, X. Xi, P. G. T. Walker, A. C. Ghani, C. A. Donnelly, S. Riley, M. A. C. Vollmer, N. M. Ferguson, L. C. Okell, S. Bhatt, Estimating the effects of non-pharmaceutical interventions on COVID-19 in Europe. *Nature* **584**, 257–261 (2020).

112. J. Abaluck, L. H. Kwong, A. Styczynski, A. Haque, Md. A. Kabir, E. Bates-Jefferys, E. Crawford, J. Benjamin-Chung, S. Raihan, S. Rahman, S. Benhachmi, N. Z. Bintee, P. J. Winch, M. Hossain, H. M. Reza, A. A. Jaber, S. G. Momen, A. Rahman, F. L. Banti, T. S. Huq, S. P. Luby, A. M. Mobarak, Impact of community masking on COVID-19: A cluster-randomized trial in Bangladesh. *Science* **375**, eabi9069 (2022).

113. Z. Zou, C. K. Fairley, M. Shen, N. Scott, X. Xu, Z. Li, R. Li, G. Zhuang, L. Zhang, Critical timing and extent of public health interventions to control outbreaks dominated by SARS-CoV-2 variants in Australia: A mathematical modelling study. *Int. J. Infect. Dis.* **115**, 154–165 (2022).

114. V. P. Bajiya, S. Bugalia, J. P. Tripathi, Mathematical modeling of COVID-19: Impact of non-pharmaceutical interventions in India. *Chaos Interdiscip. J. Nonlinear Sci.* **30**, 113143 (2020).
